# Molecular Characterization of *Tomato Brown Rugose Fruit Virus* in Portugal and Its Global Phylogenetic Context

**DOI:** 10.3390/plants15081240

**Published:** 2026-04-17

**Authors:** Joana Amaro Ribeiro, André Albuquerque, Cinthia Nunes, Maria Doroteia Campos, Margarida Basaloco, Mariana Patanita, Filipa Santos, Carla Varanda, Patrick Materatski, Maria do Rosário Félix

**Affiliations:** 1 MED Mediterranean Institute for Agriculture, Environment and Development & CHANGE Global Change and Sustainability Institute, Institute for Advanced Studies and Research, Universidade de Évora, Largo dos Colegiais 2, 7004-516 Évora, Portugal; andrealb@uevora.pt (A.A.); margarida.fonseca@uevora.pt (M.B.); mpatanita@uevora.pt (M.P.); carla.varanda@esa.ipsantarem.pt (C.V.); patrick.materatski@esa.ipsantarem.pt (P.M.); 2 Research Centre for Natural Resources, Environment and Society (CERNAS), School of Agriculture, Santarém Polytechnic University, Quinta do Galinheiro—S. Pedro, 2001-904 Santarém, Portugal; 3 Departamento de Agronomia, Universidade Federal Rural de Pernambuco, Recife 52171-900, PE, Brazil; cinthiaclaudino1@gmail.com; 4 MED Mediterranean Institute for Agriculture, Environment and Development & CHANGE Global Change and Sustainability Institute, Departamento de Fitotecnia, Escola de Ciências e Tecnologia, Universidade de Évora, Largo dos Colegiais 2, 7004-516 Évora, Portugal; mdcc@uevora.pt (M.D.C.); fs@uevora.pt (F.S.); mrff@uevora.pt (M.d.R.F.)

**Keywords:** ToBRFV, *Tobamovirus fructirugosum*, *Solanum lycopersicum*, genome sequencing, phylogenetic analysis, Portuguese isolates

## Abstract

Plant viruses pose serious threats to global crop production, and members of the genus *Tobamovirus* are particularly problematic due to their environmental stability, efficient mechanical transmission and rapid global spread. *Tomato brown rugose fruit virus* (ToBRFV) has emerged as one of the most damaging tobamovirus affecting tomato, a crop of major economic importance worldwide. ToBRFV has been reported in more than 45 countries, including Portugal. However, to date, no peer-reviewed molecular characterization of local isolates has been published, and official records classify its presence in Portugal as transient. This study confirms the occurrence of ToBRFV and provides the first comprehensive genomic and phylogenetic characterization of local virus isolates in Portugal. RNA-seq generated 192,852,438 reads, of which 103,882,115 (58.9%) mapped to ToBRFV, allowing reconstruction of a complete 6393 nt viral genome. A second full-length consensus sequence was independently obtained from the same composite sample using an overlapping Sanger sequencing strategy, differing by only two SNPs. Comparative genomic, functional, structural, and phylogenetic analysis revealed low diversity, with most variation located in replicase-coding regions, while movement and coat protein genes remained highly conserved. Nucleotide-based phylogenies resolved geographically structured clades, although the Portuguese sequences formed a strongly supported subclade with a Chinese isolate. These findings support recent global dissemination of ToBRFV and reinforce the importance of integrated surveillance and genomic monitoring for effective virus management.

## 1. Introduction

Long before the domestication of wild plants for food, fiber, medicine and ornamental purposes over the past 10,000–15,000 years, plant pathogens were already co-evolving with their wild plant hosts [1]. The transition from wild ecosystems to agricultural systems greatly altered plant–pathogen interactions. Following domestication, cultivated plants growing as genetically uniform monocultures were vulnerable to pathogens that evolved from their wild ancestors. This evolutionary process has shaped both hosts and pathogens, including plant viruses, which have undergone continuous adaptation and expansion overtime [2,3,4].

Plant viruses represent a serious threat to global agriculture production, causing substantial yield losses, reducing fruit quality and marketability, and leading to significant economic losses for producers. Viral diseases affect not only food production for humankind and livestock, with direct consequences for food security, but also fiber production, ornamental horticulture and medicinal plant industries [5,6]. As the demand for plant products continues to increase in an increasingly populated world, the consequences of plant viral diseases are expected to intensify. Since viruses account for 47% of the pathogens that cause emerging and re-emerging plant disease epidemics worldwide, there is an urgent need to study these diseases using holistic approaches to understand the main factors triggering these epidemics and develop effective control strategies [7,8].

Among plant viruses, members of the genus *Tobamovirus* have long been recognized as particularly problematic due to their exceptional environmental stability, ease of mechanical transmission, and capacity for rapid spread. Several tobamoviruses have historically caused severe outbreaks in economically important crops, leading to major yield reductions and losses in fruit quality. Well-known examples include *Tobacco mosaic virus* (TMV), *Tomato mosaic virus* (ToMV), *Tomato mottle mosaic virus* (ToMMV), *Pepper mild mottle virus* (PMMoV), and *Cucumber green mottle mosaic virus* (CGMMV) [6,9,10].

*Tomato brown rugose fruit virus* (ToBRFV), officially recognized by the International Committee on Taxonomy of Viruses (ICTV) as *Tobamovirus fructirugosum*, belongs to the genus *Tobamovirus* within the family *Virgaviridae* [10,11,12]. The ToBRFV genome consists of a single-stranded, positive-sense RNA molecule of approximately 6.4 kb, encapsidated within rigid, rod-shaped virions [6,13,14]. The genome contains four open reading frames (ORFs), capped at the 5′ terminus with a 7-methylguanosine triphosphate, and terminates at the 3′ untranslated region with three pseudoknot structures and a total RNA-like structure, a feature conserved across the genus. ORF1 and ORF2 encode the replication-associated proteins, 126 kDa and 183 kDa, respectively, which are translated directly from the genomic RNA, whereas OFR3 and ORF4 encode the 30 kDa movement protein (MP) and 17.5 kDa coat protein (CP) which are expressed from subgenomic RNAs [6,10,14,15,16]. ORF1 and ORF2 are involved in viral genome replication, with ORF2 being expressed via readthrough of an amber stop codon (UAG) downstream of ORF1. The 126 kDa replicase harbours the methyltransferase and helicase domains involved in RNA capping and unwinding, play a role in the assembly of the replication complex (replication initiation), and can also act as an RNA silencing suppressor, promoting viral accumulation by counteracting antiviral defence mechanisms. Additionally, the 183 kDa replicase contains a C-terminal RNA-dependent RNA polymerase (RdRp) domain required for viral RNA synthesis and the production of genomic and subgenomic RNAs. The MP plays a central role in cell-to-cell movement and is also one of the key determinants underlying the biological uniqueness of ToBRFV. Unlike several other tobamoviruses affecting tomato, ToBRFV is able to overcome the durable *Tm*-2^2^ resistance gene widely deployed in commercial cultivars. This resistance-breaking phenotype has been linked to specific molecular features of the movement protein, which distinguish ToBRFV from related tobamoviruses such as TMV and ToMV. The CP is essential for virion assembly, systemic movement, and the stability and transmissibility of the virus [5,6,12,17,18,19].

ToBRFV was first identified in 2015 in Jordan in greenhouse-grown tomato plants (cv. Candela) exhibiting mild foliar symptoms and severe brown rugose symptoms on fruits, with disease incidence approaching 100%. Based on the symptomatology and disease spread patterns, a viral aetiology was initially suggested [6,19,20]. Subsequent isolation, full-length genome sequencing (6393 nt; isolate Tom1-Jo; GenBank accession no.: KT383474) and comprehensive biological, molecular, and phylogenetic analyses confirmed the virus as a novel species within the genus *Tobamovirus*, which was subsequently recognized and officially named by the ICTV as *Tomato brown rugose fruit virus* [5,10,19].

Afterwards, a closely related isolate (TBRFV-IL; GenBank accession no.: KX619418), with high sequence identity to Tom1-Jo, was identified from samples collected from a severe outbreak in southern Israel in 2014, even before the formal identification of the virus. This outbreak rapidly spread across multiple tomato-growing regions, mainly because the tomato cultivars carrying the *Tm*-2^2^ resistance gene (previously effective against TMV- and ToMV-resistant tomato cultivars) were unable to confer resistance against ToBRFV. This resistance breakdown has been attributed to specific molecular determinants within the viral genome, or recombinant events that broke tomato resistance, highlighting the adaptive potential of this virus [12,21].

Following the initial detection, ToBRFV has been reported worldwide and is currently present on all five continents, in more than 45 countries, according to the European and Mediterranean Plant Protection Organization (EPPO) Global Database. Its rapid global spread reflects both efficient dissemination pathways and improved diagnostic capacity; however, discrepancies between official records and peer-reviewed reports highlight the need for cautious interpretation of country status classifications [6,11]. Such discrepancies may arise from differences in surveillance intensity, reporting timelines, delays between official detection and publication, and differences in how national authorities classify pest status. These inconsistencies can influence the perceived global distribution of the virus and may affect risk assessment, surveillance priorities, and disease management strategies.

Overall, ToBRFV occurrence has been predominantly associated with greenhouses cultivating tomato fruits, although detections have also occurred in seed production facilities, nurseries, and research institutions [20]. ToBRFV was first included in the EPPO Alert List in 2019, due to its rapid spread and potential economic impact. This list is used to draw attention to newly emerging plant pests and diseases that may present a risk and therefore warrant further evaluation. In 2020, ToBRFV was transferred to the EPPO A2 List, indicating that it was present but not widely distributed within the EPPO region and subject to official control measures. More recently, according to the EPPO Reporting Service (2025/015), ToBRFV has been reclassified as a Regulated Non-Quarantine Pest (RNQP), reflecting its widespread distribution and the limited feasibility of eradication, while maintaining regulatory oversight focused on containment and management [11].

In Portugal, ToBRFV has been officially detected in two nurseries producing tomato plantlets in Tavira and Faro (Algarve region), associated with tomato seeds imported from China and Israel, respectively. Later, the virus was detected in an industrial tomato production field in Benavente (Lisboa e Vale do Tejo region). Eradication measures were applied, and trace-forward studies were conducted to identify and retrieve potential infected plant material. No further official detections have been reported. Therefore, ToBRFV is currently classified in Portugal as transient, actionable, and under eradication, indicating that while the virus has been detected, it is not considered established and remains subject to strict phytosanitary measures [11,20].

Considering the foregoing, ToBRFV represents a serious and ongoing threat to global tomato production, which highlights the urgency for detailed molecular and evolutionary studies. The main aim of the present study is to characterize ToBRFV isolates detected in Portugal at the molecular and phylogenetic levels. This includes the generation and analysis of full-length viral genome sequences to identify genetic variation, assess functional and structural implications, and determine phylogenetic relationships with global isolates.

The significance of this work lies in providing the first peer-reviewed molecular confirmation and comprehensive genomic characterization of ToBRFV in Portugal. By contributing with novel sequence data and global phylogenetic insights, this study enhances understanding of the virus’s genetic diversity, evolution, and spread. The insights gained from this study will contribute to risk assessments and diagnostic improvements, and facilitate the development of more effective management and containment strategies. Ultimately, this will support the protection of tomato crops and the sustainability of the tomato industry in Portugal and beyond.

## 2. Results

### 2.1. ToBRFV Sampling and Identification

Processing tomato field samples showing typical symptoms of *Tomato brown rugose fruit virus* (ToBRFV), such as brown and wrinkled patches on fruit surface, leaf deformation and narrowing, mosaic or mottling, and reduced vigor and yield, were collected. Total RNA extraction from the composite sample was highly efficient, yielding a concentration of 1372.88 ng/μL with purity ratios of 2.215 (A260/280) and 2.176 (A260/230). Following cDNA synthesis, the sample was screened for the presence of ToBRFV, and other common viruses in tomato, such as *Tomato mosaic virus* (ToMV), *Tobacco mosaic virus* (TMV) and *Tomato spotted wilt virus* (TSWV).

**Figure 1:**
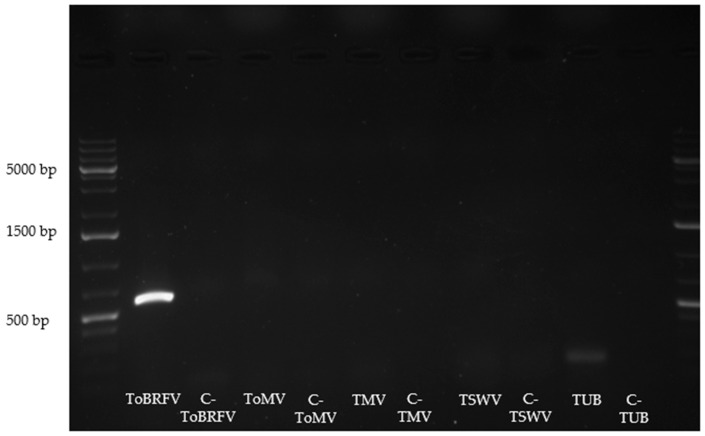
Agarose gel showing the RT-PCR results to test the presence of *Tomato brown rugose fruit virus* (ToBRFV). Fragment sizes, according to the Thermo Scientific™ GeneRuler 1 kb Plus DNA Ladder (Thermo Fisher Scientific, Waltham, MA, USA), are shown on the left side of the figure for the three most intense DNA bands, and each target is identified at the bottom.

Reverse transcription PCR (RT-PCR) confirmed the presence of ToBRFV in the tested sample. No amplification was observed for the other viral targets included in the assay, supporting the specificity of the ToBRFV detection. Amplification of the tubulin gene (*TUB*) served as an internal reference, and no amplification was obtained in the negative controls included for each target (Figure 1).

### 2.2. Molecular Characterization of ToBRFV Portuguese Isolates

#### 2.2.1. Genome Sequencing, Sequence Analysis and Gene Annotation

Genome sequencing of the Portuguese ToBRFV-positive composite sample was performed using RNA sequencing (RNA-seq), which generated a total of 192,852,438 reads. After filtering out sequences matching the tomato host genome and retaining only those matching the plant virus database, based on NCBI Reference Sequences, multiple plant viruses were detected. Among these, ToBRFV was the most prevalent, exhibiting a markedly higher number of reads compared to all other detected viruses. Additional plant viruses were also identified, including *Pepino mosaic virus*, *Potato aucuba mosaic virus*, *Malva mosaic virus*, *White clover mosaic virus*, *Plantain virus X*, *Indian citrus ringspot virus*, and *Tomato chlorosis virus*, among others, in decreasing order of read abundance.

**Figure 2:**
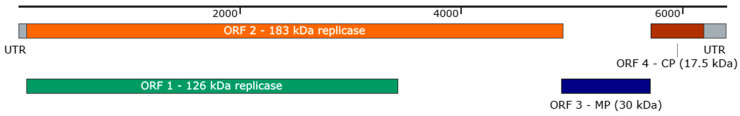
Diagram representing the organization of ORFs throughout the *Tomato brown rugose fruit virus* (ToBRFV) genome. Figure generated on SnapGene Viewer (version 8.0.3).

Using the ToBRFV reference sequence NC_028478.1 (corresponding to Tom1-Jo; GenBank accession no.: KT383474), a total of 103,882,115 reads were retained (58.9% of the total reads). Although high, this proportion is consistent with the symptomatic nature of the analysed field material and suggests a high viral load in the composite sample. According to the RNA-seq results, the total covered region was 6393 nucleotides (nt), corresponding to the full length of the reference genome, indicating that no regions of the viral genome were left unsequenced. A consensus genome sequence was generated using all reads matching the ToBRFV reference sequence. This sequence was designated ToBRFV_PT1 and deposited in the NCBI database under GenBank accession number PV978367.1. The full sequence is provided in Supplementary Materials Data S1. Classical nucleotide analysis identified four ORFs for gene annotation (Figure 2). ORF1 corresponds to the 126 kDa replicase with a total of 3351 nt and is located between nt 73 and 3423. ORF2 is located between nt 73 and 4920, spanning 4848 nt, and encodes the 183 kDa replicase. The 30 kDa movement protein, ORF3, with a length of 801 nt, extends from nt 4907 to nt 5707. ORF 4 encodes the 17.5 kDa coat protein with a total of 480 nt, and spans nt 5710 to 6189. From the 5′ end to nt 73 is the first untranslated region (UTR) and from nt 6189 to the 3′ end is the second UTR.

**Figure 3:**
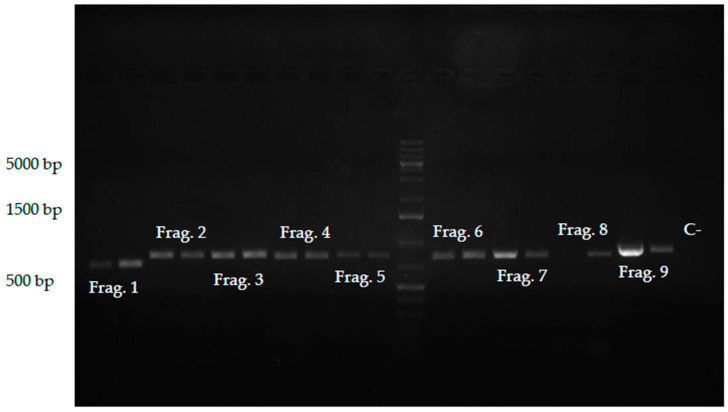
Agarose gel showing the RT-PCR results for *Tomato brown rugose fruit virus* (ToBRFV) fragment amplification. Fragment sizes, according to the Thermo Scientific™ GeneRuler 1 kb Plus DNA Ladder, are shown on the left side of the figure for the three most intense DNA bands, and each target is labeled accordingly.

Sanger sequencing was performed as an additional and independent sequencing approach on the same composite sample, using different overlapping fragments amplified and sequenced, in both sense and anti-sense directions, which allowed the generation of a second full-length consensus sequence. Amplification of the fragments was accomplished using a gradient PCR, with the specific primer pairs designed, with expected product sizes between 720 and 889 bp (Figure 3). Sanger sequencing results for each fragment and sequence alignment are shown in Supplementary Materials Data S1. The resulting consensus sequence was designated ToBRFV_PT2 and deposited in the NCBI database under GenBank accession number PV978368.1. The full sequence is available in Supplementary Materials Data S1.

#### 2.2.2. Sequence Alignment and Comparative Analysis

**Figure 4:**
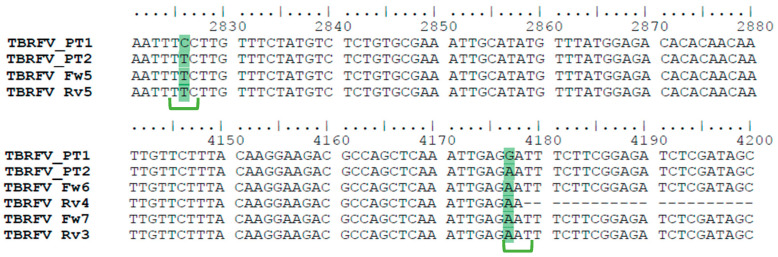
Sequence alignment highlighting (in green) differences between consensus sequences from RNA-seq analysis (ToBRFV_PT1) and Sanger sequencing analysis (ToBRFV_PT2). Triplet codons encoding the amino acids of interest are marked with braces.

**Figure 5:**
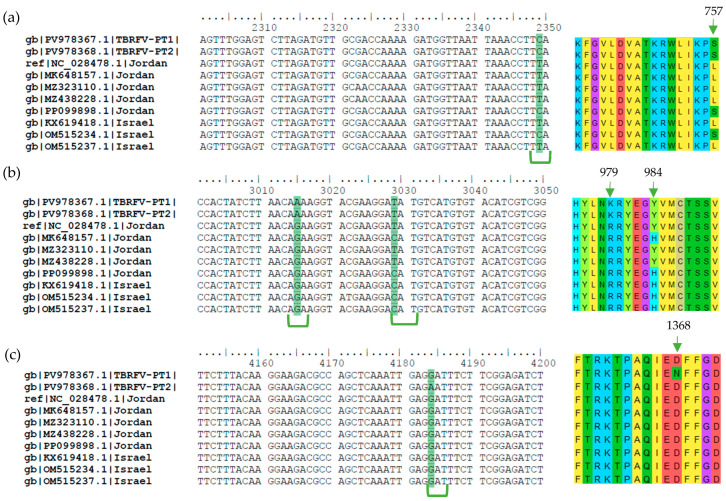
Multiple-sequence alignment showing the first few isolates of the complete alignment with the positions of interest. GenBank accession numbers and country of origin of the isolates are listed on the left side of the figure. Non-synonymous SNPs are highlighted (in green) in the nt sequences and with a green arrow in the corresponding amino acid sequence, on the right side of the figure. The numbers above the arrows represent the position of the mutation in amino acid sequences. Triplet codons encoding the amino acids of interest are marked with braces. (**a**) Positions from nt 2300 to 2350. (**b**) Positions from nt 3000 to 3050. (**c**) Positions from nt 4150 to 4200.

Consensus sequences from RNA-seq (ToBRFV_PT1) and Sanger sequencing (ToBRFV_PT2) were aligned and compared. The alignment revealed two single nucleotide polymorphisms (SNPs) between the two consensus sequences (Figure 4, full alignment in Supplementary Materials Data S1), although both were obtained from the same composite sample. Therefore, these sequences are interpreted as two independently reconstructed consensus genomes from the same infected material, rather than as two independently sampled biological isolates. The first SNP occurs at nt 2826 and represents a transition where a pyrimidine is replaced with another pyrimidine (C ↔ T), resulting in a synonymous substitution (TTC ↔ TTT, both encoding Phenylalanine (Phe, F)). The second SNP occurs at nt 4177 and represents a transition where a purine is replaced with another purine (A ↔ G), however, in this case resulting in a non-synonymous substitution (GAT ↔ AAT) which results in two different amino acids, Aspartic acid (Asp, D) in ToBRFV_PT1 and Asparagine (Asn, N) in ToBRFV_PT2. This mutation, located at position 1368 of the amino acid sequence, is hereafter referred to as D1368N (Figure 5c).

To achieve a more complete and detailed understanding of the molecular characteristics of the ToBRFV Portuguese sequences, these were aligned and compared with 298 complete genomes available on the NCBI Virus platform, including the reference sequence Tom1-Jo (NC_028478.1).

Nucleotide comparisons between the Portuguese sequences, the reference sequence, and the 298 additional isolates revealed 13 SNPs where ToBRFV_PT1 and ToBRFV_PT2 shared the same nt, differing from all or most of the other sequences. It should be noted that all nucleotide positions are referenced according to the ToBRFV_PT1 annotation (GenBank accession no.: PV978367.1), which does not exactly correspond to positions observed in the multiple-sequence alignment, as the UTRs contain deletions or additional nt across isolates. For clarity, the corresponding alignment positions are indicated in parentheses throughout the text.

Nine of these SNPs were located within the region encoding the 183 kDa and 126 kDa replicases. Only three resulted in non-synonymous substitutions: at nt 2342 (nt 2349), nt 3008 (nt 3015), and nt 3022 (nt 3029). The SNP at nt 2342 (nt 2349) is a transition between pyrimidines (C ↔ T), resulting in a codon change from TTA to TCA, which alters the encoded amino acid from Leucine (Leu, L), as found in the reference and most other sequences, to Serine (Ser, S), present in both Portuguese sequences (Figure 5a). This mutation, located at position 757 of the amino acid sequence, is hereafter referred to as L757S (Figure 5a). The SNP at nt 3008 (nt 3015) is a transition between purines (A ↔ G), resulting in a codon change from AGA to AAA, and an amino acid substitution from Arginine (Arg, R), as found in the reference and most other sequences, to Lysine (Lys, K) in ToBRFV_PT1 and ToBRFV_PT2 (Figure 5b). This mutation, located at position 979 of the amino acid sequence, is hereafter referred to as R979K (Figure 5b). The SNP at nt 3022 (nt 3029) is a transition between pyrimidines (C ↔ T) that shows a codon change from CAT to TAT, which alters the encoded amino acid from Histidine (His, H) to Tyrosine (Tyr, Y). In this case, both Portuguese consensus sequences encoded Tyrosine (Tyr, Y), matching the reference sequence Tom1-Jo and a small subset of global sequences, whereas the majority of the remaining sequences encoded Histidine (His, H) at this position (Figure 5b). This mutation, located at position 984 of the amino acid sequence, is hereafter referred to as H984Y (Figure 5b). Two of the 13 initially identified SNPs were located within the 183 kDa specific C-terminal region and were synonymous. The remaining two SNPs were located within the MP coding region and were also synonymous. No SNPs distinguishing the Portuguese sequences from most other sequences were detected within the CP coding region.

Regarding the two SNPs distinguishing ToBRFV_PT1 and ToBRFV_PT2, the synonymous SNP at nt 2826 showed that ToBRFV_PT2 carried the nucleotide state found in the majority of global sequences (297 out of 298 sequences; 99.7%), whereas ToBRFV_PT1 differed at this position. In contrast, the non-synonymous SNP at nt 4177 (nt 4184 in the alignment), resulting in the D1368N substitution, was unique to ToBRFV_PT2, with all other analyzed sequences (298 out of 298; 100%), including ToBRFV_PT1 and the reference sequence, carrying Aspartate (Asp, D) at this position (Figure 5c). The first SNP is located within the region encoding both the 183 kDa and 126 kDa replicases, while the second SNP is located exclusively within the coding region of the 183 kDa replicase.

#### 2.2.3. Functional and Structural Implications of Non-Synonymous Substitutions

Non-synonymous SNPs were further investigated to evaluate their potential functional and structural consequences for replicase proteins. Predictions of functional impact allowed complementation and cross-validation of the results. The analysis was conducted using both the reference sequence Tom1-Jo (NC_028478.1) and an isolate from Israel (GenBank accession no.: OM_515237.1), selected because it differs from the reference only at the H984Y position.

Overall, none of the identified substitutions were predicted to have major functional consequences in either 183 kDa or 126 kDa replicases, regardless of the input sequence used for the analysis (reference Tom1-Jo or Israeli isolate sequences). Across all analysis, the mutations L757S, R979K, H984Y, and D1368N were consistently classified as “Tolerated” by SIFT and “Benign” by PolyPhen-2 and exhibited MutPred2 scores below functional impact thresholds (Supplementary Materials Table S1).

**Figure 6:**
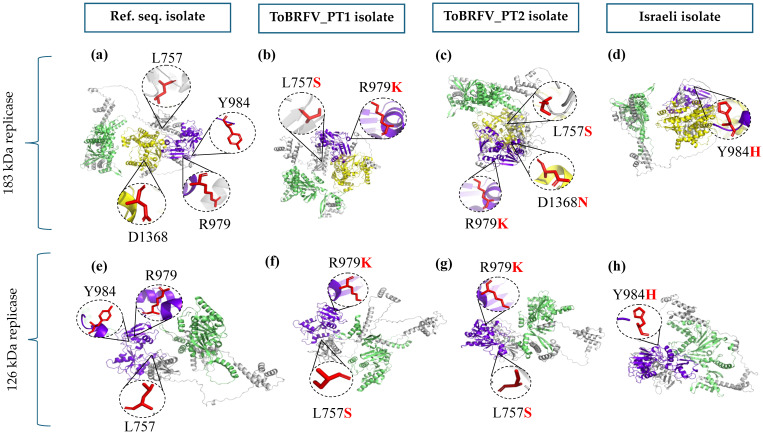
Comparative structural modeling of the 186 kDa (**a**–**d**) and 126 kDa (**e**–**h**) proteins highlighting the mutated residues across the different analyzed variants. The residues are shown in red and enlarged in circular insets for improved visualization. Structural domain colors were kept consistent across models to facilitate visual comparison among variants (Methyltransferase domain in green, Helicase domain in purple, and RdRp domain in yellow). (**a**) 183 kDa replicase protein model of the reference sequence, highlighting residues L757, R979, Y984 and D1368 in their reference conformation. (**b**) 183 kDa replicase protein model of the ToBRFV_PT1 isolate, highlighting mutations L757S, R979K. (**c**) 183 kDa replicase protein model of the ToBRFV_PT2 isolate, highlighting mutations L757S, R979K and D1368N. (**d**) 183 kDa replicase protein model of the Israeli isolate, highlighting mutation Y984H. (**e**) 126 kDa replicase protein model of the reference sequence, highlighting residues L757, R979 and Y984 in their reference conformation. (**f**) 126 kDa replicase protein model of the ToBRFV_PT1 isolate, highlighting mutations L757S, R979K. (**g**) 126 kDa replicase protein model of the ToBRFV_PT2 isolate, highlighting mutations L757S and R979K. (**h**) 126 kDa replicase protein model of the Israeli isolate, highlighting mutation Y984H.

Structural effects were evaluated using three-dimensional protein models generated with AlphaFold3. High-confidence models (“model_0”) were produced for both replicase proteins from the reference sequence, the Portuguese sequences, and the Israeli isolate. The comparative structural analysis revealed no major conformational differences attributable to the identified substitutions. Three-dimensional models for the 183 kDa and 126 kDa replicases across the different analyzed variants are presented in Figure 6, highlighting the mutated residues and protein domains. Structural modelling validation indicated acceptable structural quality, with QMEAN scores ranging from 0.58 to 0.63, 90.2–93.5% of residues located in favored regions of Ramachandran plots, and ProSA Z-scores between −11.64 and −15.05 (Supplementary Materials Table S2 and Figures S1–S8).

### 2.3. Phylogenetic Analysis

**Figure 7:**
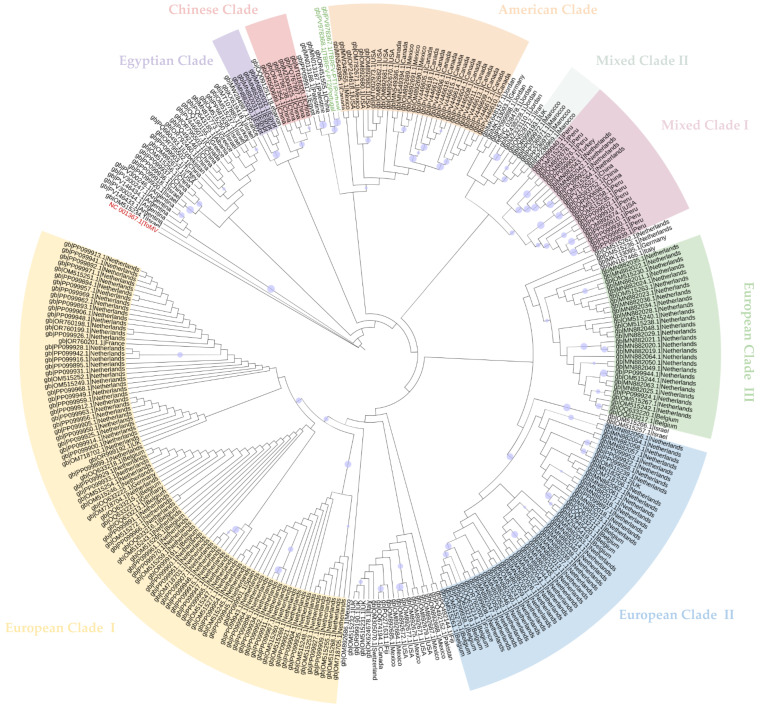
Maximum Likelihood phylogenetic tree inferred from the coding-complete nucleotide sequences of *Tomato brown rugose fruit virus* (ToBRFV) isolates. The analysis was performed under the General Time Reversible model with gamma-distributed rate variation (GTR + G) with 1000 bootstrap replicates. The tree was rooted using ToMV as an outgroup (red label). Bootstrap support values ≥ 65% are shown at the nodes, and indicated by purple circles, with larger circles representing higher support. Portuguese isolates are highlighted in green labels. Branch lengths are not scaled to improve visualization. Major clades are highlighted in different colors.

Maximum Likelihood (ML) phylogenetic analysis of coding-complete nucleotide sequences revealed patterns of genetic relatedness, geographic clustering, lineage diversification, and possible transmission or spread. The General Time Reversible model [22] with gamma-distributed rate variation (GTR + G) was selected as the best-fitting nucleotide substitution model. The resulting ML phylogenetic tree for coding-complete nucleotide sequences is shown in Figure 7.

The ToMV outgroup was clearly separated from the ToBRFV isolates, with a branch length of 0.218 substitutions per site. Overall, branch lengths values among ToBRFV isolates were very short (<0.002), indicating low genetic diversity. Considering bootstrap support values ≥65% and clades comprising more than three isolates, eight strongly supported ToBRFV clades were identified, with isolates within each clade sharing a common ancestor. Most clades exhibited clear geographic clustering; however, two clades showed geographically mixed compositions and were designated Mixed Clade I and Mixed Clade II. Mixed Clade I is predominantly composed of isolates from Peru, but also includes isolates from the USA, China, The Netherlands, and Turkey. Mixed Clade II consists mainly of isolates from Morocco, with one isolate from the UK. Three large and well-supported European clades were identified, largely composed of isolates from the Netherlands, which is highly overrepresented in the dataset. European Clade I includes mainly isolates from the Netherlands as well as from France, the UK, Belgium, and Spain. European Clade II shows a similar geographic distribution, with most isolates from the Netherlands, but also from France, Belgium, and the UK. European Clade III is composed almost exclusively of isolates from the Netherlands, with only two isolates from Belgium. An American Clade was also identified, comprising mostly Canadian isolates together with isolates from Mexico and the USA. In addition, one monophyletic clade containing only Chinese isolates and another containing only Egyptian isolates were identified. The remaining isolates could not be confidently assigned to any clade due to bootstrap support values below 65%.

The Portuguese consensus sequences clustered together and formed a strongly supported subclade with a Chinese isolate (bootstrap > 90%), rather than grouping within any of the major European clades. However, although this Portuguese–Chinese subclade was well supported, the deeper node connecting this subclade to the rest of the tree showed insufficient bootstrap support to confidently assign it to a broader major lineage. Accordingly, these sequences remained phylogenetically distinct but unresolved at deeper levels. Consistently, lineage assignment using the Nextstrain/Nextclade platform [23,24] was inconclusive, and these sequences were classified as “unassigned”.

Pairwise distance analysis based on coding-complete nucleotide sequences confirmed low intraspecific diversity (mean p-distance = 0.0034). Several pairs of distinct ToBRFV isolates exhibited zero pairwise distance. In contrast, substantially higher genetic distances were observed between ToBRFV and the ToMV outgroup (mean p-distance = 0.1862).

For phylogenetic analysis based on amino acid sequences, the Jones–Taylor–Thornton model [25] with gamma-distributed rate variation (JTT + G) was selected for the 183 kDa and 126 kDa replicase and movement protein, whereas a model with uniform rates (JTT) was applied to the coat protein, as these were identified as the best-fitting amino acid substitution models for each case.

ML phylogenetic trees inferred from the 183 kDa replicase, 126 kDa replicase, MP and CP coding regions (Supplementary Materials Figures S9–S12) revealed a consistent separation between ToBRFV and the ToMV outgroup. Across all proteins, branch lengths within ToBRFV were short and most internal nodes showed low bootstrap support. The replicase-based trees exhibited similar topologies and limited but detectable sub-structuring, as a few supported clusters (bootstrap > 65%) were identified, including a small Netherlands-specific sub-clade comprising only three isolates despite strong overrepresentation of that country, two distinct Canadian sub-clades, and a clade of four Egyptian isolates (Supplementary Materials Figures S9 and S10). The MP tree showed a different grouping pattern, with most internal nodes weakly supported. A small number of supported clusters (bootstrap > 65%) were detected, including a USA-specific sub-clade, a Netherlands sub-clade containing only four isolates, and a Canadian sub-clade resembling one of the Canadian clusters observed in the replicase trees (Supplementary Materials Figure S11). In contrast, the CP tree displayed a highly dispersed topology with little internal structure. No supported clusters were recovered with bootstrap values above 65%, providing almost no phylogenetic resolution due to extreme sequence conservation (Supplementary Materials Figure S12).

The Portuguese genomes clustered together with bootstrap values above 65% in the replicase-based phylogenies; however, they could not be confidently placed within a broader lineage due to weak support for surrounding nodes. In the MP tree, the Portuguese genomes did not appear as a distinct pair, although no amino acid differences were detected between them. Instead, they were embedded within a large cluster in which branch lengths among isolates were zero. A similar pattern was observed in the CP tree, where the Portuguese genomes appeared together but with bootstrap support below 65%.

## 3. Discussion

### 3.1. Detection and Molecular Confirmation of ToBRFV in Portugal

*Tomato brown rugose fruit virus* (ToBRFV) is a relatively recent member of the genus *Tobamovirus* that has rapidly emerged as a major threat to tomato production worldwide. Disease outbreaks can be extremely severe, with incidence levels approaching 100%, raising major concerns for growers and, given the importance of tomato as a staple food crop, for the broader population [6,9,19]. Tomato is among the most economically significant and widely cultivated crops globally, valued for its nutritional content [13]. This highlights the importance of studying this virus, including its control and management strategies as well as its true global distribution.

Despite extensive efforts to limit the spread of ToBRFV, particularly within the EPPO region, the virus has proven extremely difficult to eradicate. This is largely due to its high environmental stability and highly efficient mechanical transmission that may occur through direct plant-to-plant contact, contaminated tools, soil or substrates, and infected seeds. Viral particles may be present in the seed coat, and, in some cases, in the endosperm, although they have not been detected in the embryo [5,13]. Consequently, according to the EPPO Reporting Service (2025/015), ToBRFV has been reclassified as a Regulated Non-Quarantine Pest (RNQP). This designation applies to pests that are already widely established and no longer feasible to eradicate, yet continue to cause economic damage and therefore remain subject to official regulation, with emphasis on containment and management rather than elimination [11].

However, current control and management strategies for ToBRFV have very limited long-term effectiveness because they depend largely on strict phytosanitary measures, such as the use of certified virus-free seeds [26,27,28], disinfection of tools and substrates, crop rotation, elimination of alternative hosts, and rapid removal of infected plants [11,29]. Nevertheless, due to the high environmental stability of the virus and efficiency of mechanical transmission, these practices frequently fail to prevent reinfection and new outbreaks, particularly under intensive production systems [12,18].

Therefore, the development of resistant or tolerant tomato varieties is regarded as the most promising long-term strategy for ToBRFV management. Although resistance mediated by the *Tm*-2^2^ gene has been effective against several tobamoviruses, ToBRFV is able to overcome this resistance, rendering many commercial cultivars susceptible [17]. Recent breeding efforts have identified novel sources of partial resistance in *Solanum* species [30,31,32], and several commercial hybrids with improved tolerance are currently under evaluation; however, fully resistant cultivars remain unavailable. Notably, De Ruiter^®^ (Vegetables by Bayer) has already available tomato varieties exhibiting intermediate resistance to ToBRFV and continues to investigate additional resistance sources [33]. In parallel, advanced breeding and biotechnological approaches, including CRISPR/Cas-based strategies, are being explored to achieve more durable resistance [34,35]. Nevertheless, the long-term effectiveness of these approaches under high viral pressure remains uncertain, reinforcing the need for further studies on ToBRFV epidemiology and management.

In this context, the present study provides the first peer-reviewed scientific confirmation of ToBRFV in Portugal. Molecular detection of ToBRFV in symptomatic processing tomato plants collected from a field in Torres Vedras unequivocally demonstrates its presence in the country. The absence of amplification for other common tomato viruses, together with the successful amplification of the internal control, supports the reliability of the diagnostic approach and strongly indicates ToBRFV as the causal agent of the observed symptoms.

To the best of our knowledge, no previous peer-reviewed studies have documented ToBRFV in Portugal. This finding, combined with unofficial reports from growers describing symptoms consistent with ToBRFV infection, suggests that the virus may be more widely established than currently recognized. Although Portugal is officially classified by EPPO as having a “transient” ToBRFV status, indicating recent detection without confirmed establishment [11], our results suggest that this status may warrant reevaluation. Nonetheless, broader surveys and expanded sampling across different tomato growing-regions are required to accurately assess the distribution, prevalence, and epidemiological status of ToBRFV within the country.

Similar discrepancies between official classifications and actual virus prevalence may also occur elsewhere, particularly in regions with limited surveillance, restricted access to diagnostic tools, or delays in scientific reporting. These gaps highlight the importance of harmonized monitoring programs, improved diagnostic capacity, and timely dissemination of scientific data to accurately assess the global distribution and impact of ToBRFV. Future studies including a larger number of isolates, broader geographic sampling, and integration with epidemiological and trade data will be essential to better understand the introduction pathways and local transmission dynamics.

To further characterise the Portuguese isolate, RNA-seq-based analysis was performed, yielding complete coverage of the viral genome with high sequencing depth and confirming the integrity and reliability of the obtained consensus sequence (ToBRFV_PT1). The high proportion of reads mapping to the viral genome indicates a high viral load in the analysed composite sample, supporting the suitability of RNA-seq for comprehensive viral genome characterization [36,37]. The observed genomic organization of the Portuguese isolate, comprising four ORFs encoding the two replicase proteins, the movement protein, and the coat protein, was fully consistent with the canonical genome structure reported for ToBRFV and other members of the genus *Tobamovirus* [12,19].

To independently validate the RNA-seq results, a complementary Sanger sequencing strategy was used and further strengthened these findings. This approach allowed the generation of an independent full-length consensus sequence (ToBRFV_PT2) and provided an additional layer of validation for the viral genome obtained by high-throughput sequencing. The use of multiple complementary primers minimizes the risk of sequencing artefacts and primer-specific bias, and represents a robust and cost-effective approach for virus genome confirmation, particularly in diagnostic and surveillance contexts where next-generation sequencing may not be routinely available [38,39].

Notably, two consistent SNPs were identified between the RNA-seq and Sanger consensus sequences (ToBRFV_PT1 and ToBRFV_PT2, respectively). These differences are unlikely to result from sequencing errors, as the RNA-seq consensus was derived from a very large number of reads, while the Sanger-based consensus was independently confirmed using multiple genomic overlapping genomic fragments that were amplified, sequenced and aligned in both directions (5′ and 3′) with different primer pairs.

Several biologically plausible explanations may account for the observed differences between the two sequences. One possibility is that different cDNAs, although synthesized from the same total RNA, were used as templates for the two sequencing approaches, thereby capturing distinct viral variants present in the total RNA sample. The difference may also reflect intra-host genetic diversity, with multiple viral genomic variants coexisting within a single plant or, in this case, single composite sample that included more than one plant. Such heterogeneity is a well-documented feature of RNA viruses and results from the error-prone nature of RdRp, leading to the formation of closely related viral variants (quasispecies) [40,41].

From an integrated perspective, the combined use of RNA-seq and the developed method for Sanger sequencing with multiple complementary primers proved highly effective not only for confirming the presence of ToBRFV in Portugal, but also for revealing underlying genomic variability within infected plants. This integrative approach strengthens the reliability of molecular characterization and highlights the importance of employing complementary sequencing methodologies when studying emerging plant viruses and documenting new geographical occurrences.

### 3.2. Genomic Conservation and Limited Genetic Variability of ToBRFV

Comparative genomic analysis of the Portuguese sequences against a large number of complete ToBRFV genomes revealed remarkably low genetic variability across the species. This finding is consistent with previous reports describing ToBRFV as a recently emerged virus characterized by rapid global dissemination and limited evolutionary diversification [42]. Low nucleotide diversity and strong purifying selection are well-documented features among the genus *Tobamovirus*, reflecting strong functional constraints on essential proteins involved in replication, movement, and encapsidation [43,44].

Such genomic stability in tobamoviruses has been attributed to these strong functional constraints acting on key viral proteins, such that mutations affecting core functions tend to be eliminated, thereby contributing to the relative stability of the viral genome [43,44]. In the present study, most nucleotide variability distinguishing the Portuguese sequences from the reference sequence and the majority of other isolates was located within the replicase-coding regions. In contrast, the MP and CP were highly conserved, with only synonymous substitutions detected in MP and none in the CP. This pattern suggests strong purifying selection acting on these proteins, likely reflecting their critical roles in cell-to-cell movement, virion assembly, and long-term environmental stability, key traits underpinning the epidemiological success of ToBRFV [42,45].

Although replicase-encoding regions showed relatively higher variability, most detected SNPs were synonymous, and only a small number resulted in amino acid substitutions. The predominance of mutations in replicase-encoding regions has also been reported for other members of the genus *Tobamovirus* and RNA plant viruses in general, and may indicate that replicase proteins tend to tolerate a greater degree of sequence variation while maintaining essential functions [44,46].

The apparent genetic uniformity of ToBRFV across continents supports the hypothesis of recent and repeated introductions, most likely via contaminated plant material, particularly seeds, rather than long-term regional evolution. Similar observations of low diversity and high sequence similarity in global populations have been reported, with very low nucleotide divergence observed among isolates from diverse geographic regions, consistent with dissemination through seed trade and recent spread dynamics [47]. Over the past few decades, the global situation of plant viral diseases has deteriorated significantly, driven by a combination of ecological, agricultural, and economic factors. The key factors for the worsening situation are the rapid expansion of international trade in plants and the rise of multinational seed companies, the movement of crop plants away from their domestication centers to distant countries, and increasing climate instability [9]. Together, these factors have facilitated the introduction of damaging viral diseases in parts of the world where they were previously absent. As a result, such diseases are emerging at an accelerating rate and becoming increasingly difficult to manage under unstable agricultural conditions [2,8,9].

### 3.3. Biological Relevance of the Identified Non-Synonymous Substitutions

Within the highly conserved genomic background of ToBRFV, non-synonymous SNPs were rare and restricted to a small number of positions within replicase-coding regions. Among all analysed genomes, only a limited set of amino acid changes distinguished the Portuguese sequences from the reference sequence and the majority of global isolates, and no unique non-synonymous SNPs were detected in the MP or CP. This low level of amino acid variation is consistent with previous large-scale genomic analysis, which have reported that most polymorphisms occur at low frequency and are predominantly synonymous, reflecting the strong purifying selection acting on functionally constrained viral proteins [21,24,48].

Functional impact predictions performed using multiple independent tools consistently indicated that the identified amino acid substitutions are unlikely to cause major functional alterations in either the 183 kDa or 126 kDa replicases. These predictions remained stable across different reference inputs, supporting their robustness. This observation is also consistent with the previously mentioned tendency for a predominance of mutations in replicase-encoding regions reported for the genus *Tobamovirus* and other RNA plant viruses, indicating that these proteins may tolerate a greater degree of sequence variation while maintaining functionality [44]. Such tolerance to minor amino acid changes reflects the structural and functional robustness of the replication complex, allowing minor substitutions without comprising essential enzymatic activities [49].

Structural modelling further supported these findings, revealing no major perturbations in protein folding or domain organization associated with the identified substitutions. The mutated residues were spatially dispersed and were not associated with major structural rearrangements, suggesting a low likelihood of substantial biological effects. Similar observations have been reported in structural and evolutionary analysis of tobamovirus replicases, where rare non-synonymous substitutions generally show limited structural impact and are not associated with phenotypic shifts [43,46].

Taken together, the rarity, predicted functional neutrality, and limited structural impact of these non-synonymous substitutions suggest that they represent neutral variation rather than adaptative changes. While subtle effects on viral fitness or host interactions under specific environmental conditions cannot be excluded, the available evidence indicates that these mutations are unlikely to confer major biological or epidemiological advantages [44,50].

### 3.4. Phylogenetic Relationships and Genetic Diversity of ToBRFV Sequences

Phylogenetic analysis based on both nucleotide and amino acid sequences of each viral protein provided complementary aspects of ToBRFV genetic diversity, population structure, and evolutionary history. Nucleotide-based phylogenies, which incorporate both synonymous and non-synonymous substitutions, are particularly informative for assessing fine-scale genetic variation, recent evolutionary events, and patterns of geographic dispersion, whereas amino acid-based phylogenies reduce noise introduced by silent mutations and are more suitable for evaluating deeper evolutionary relationships, functional conservation, and long-term divergence at the protein level [51,52]. The combined use of these approaches allows a more comprehensive interpretation of ToBRFV population structure while accounting for the overall low genetic variability of the virus.

The nucleotide-based ML phylogeny revealed extremely short branch lengths among ToBRFV sequences, consistent with the low pairwise genetic distances observed and supporting the limited global genetic diversity of the virus. At first, this low overall diversity may appear inconsistent with the presence of several geographically structured clades. However, these patterns are not contradictory and are consistent with a recently emerged virus that has undergone rapid global dissemination from a limited number of closely related founding variants. Under such a scenario, founder effects, repeated introductions, local amplification, and human-mediated dissemination through infected seeds, seedlings, fruits, or contaminated materials may generate partial geographic clustering despite minimal overall sequence divergence [42,43,47].

Several well-supported clades showed a degree of geographic structuring, largely in agreement with previously described phylogenetic studies [5,6,13]. In particular, the major nucleotide-based clades identified in this study are broadly consistent with those defined by the Nextstrain platform [23]. However, the presence of mixed clades comprising isolates from multiple regions highlights the role of recent, human-mediated dissemination events and underscores the challenges of inferring transmission routes solely from phylogenetic data.

The Portuguese consensus sequences clustered together as expected and formed a strongly supported subclade with a Chinese isolate, rather than grouping with other European sequences. Although this Portuguese–Chinese subclade was strongly supported, deeper nodes lacked sufficient bootstrap support to confidently place it within a broader lineage. Consistently, lineage assignment using the Nextstrain/Nextclade platform [23,24] was inconclusive. Similar patterns of unexpected geographic clustering among closely related ToBRFV sequences have been reported in other studies and are generally interpreted in the context of the recent and rapid global dissemination of the virus, likely driven by international trade of contaminated plant material or seeds. Such patterns are consistent with a scenario in which many outbreaks trace back to a limited number of closely related ancestral variants disseminated through shared seed lots or common sources of inoculum [5,6,26]. Importantly, although the phylogenetic proximity between the Portuguese sequences and a Chinese sequence is epidemiologically suggestive, it should not be interpreted as evidence of a direct introduction route without direct epidemiological or trade-related data. Nevertheless, this pattern highlights the importance of targeted future surveillance integrating genomic data with epidemiological, phytosanitary, and trade-related information. In particular, future genomic surveillance in Portugal would benefit from increased sampling across production systems and from systematic comparison with sequences from regions and supply chains potentially connected to previous introductions.

Pairwise distance analysis further supported this interpretation revealing identical nucleotide sequences among samples from distinct geographic regions. Such findings emphasize that phylogenetic proximity does not necessarily imply direct epidemiological linkage and should therefore be interpreted cautiously [47].

Amino acid-based phylogenetic analysis provided limited additional resolution due to the extreme conservation of ToBRFV proteins, particularly the movement and coat proteins. Across all viral proteins, internal branches were short and bootstrap support was generally low, reflecting the high degree of protein sequence conservation and low diversity. This result is consistent with the strong purifying selection expected for essential viral proteins involved in replication, movement, encapsidation, and host interaction, and reinforces the notion that most recent ToBRFV diversification is best captured at the nucleotide rather than amino acid level.

Comparison of phylogenetic trees inferred from the 183 kDa replicase, 126 kDa replicase, MP and CP coding regions revealed a consistent separation between ToBRFV and the ToMV outgroup, confirming the robustness of the analysis and supporting a strong interspecific divergence relative to intraspecific variation [24,26]. However, while replicase-based trees exhibited similar topologies and limited but detectable sub-structuring, phylogenies inferred from the MP and, especially, the CP provided little resolution beyond species-level separation. This lack of resolution is consistent with the extreme conservation of these proteins and strong purifying selection acting on structural and movement-related functions in tobamoviruses [6,21,48].

The two Portuguese sequences clustered together in the replicase-based amino acid phylogenies, forming a well-supported subclade and further supporting their close evolutionary relationship, but they could not be robustly assigned to a broader lineage, as happened for the nucleotide-based tree. In the MP and CP trees, their placement within large groups of identical sequences highlights the limited phylogenetic signal available in these regions. Overall, these findings indicate that while replicase regions provide modest resolution for investigating ToBRFV population structure and evolutionary relationships, the extreme conservation of MP and CP constrains their usefulness for fine-scale evolutionary inference [6,26,43].

Altogether, the combined phylogenetic analysis highlights the remarkably low genetic diversity of ToBRFV at a global scale. While nucleotide data provide evidence for recent dissemination with some geographic structuring, the high level of protein conservation constrains deeper evolutionary inference. These results support the characterization of ToBRFV as a recently emerged virus undergoing rapid global spread from a restricted genetic pool and highlight the need for caution when inferring transmission routes or geographic origins.

## 4. Materials and Methods

### 4.1. ToBRFV Sampling and Identification

#### 4.1.1. Sample Collection

Tomato plant samples used in this study to identify *Tomato brown rugose fruit virus* (ToBRFV) Portuguese isolates were collected by the end of 2023 growing season from a processing tomato field located in Torres Vedras, in the Oeste region of Portugal (39°08′10″ N, 8°33′00″ W, approximate coordinates provided to preserve field confidentiality). In this region, the growing season extends from March to October. The 2023 season was characterized by exceptionally hot and dry conditions, particularly in some regions such as Oeste (39°08′10″ N, 8°33′00″ W) [53]. Whole tomato plants showing typical symptoms of ToBRFV were collected and immediately frozen in liquid nitrogen, and transported to the laboratory, where they were promptly processed for downstream analysis.

#### 4.1.2. Total RNA Extraction

Three leaves per plant were detached from six symptomatic tomato plants and pooled to form a composite sample. Samples were ground to a fine powder using a sterile mortar and pestle, aiding the process with liquid nitrogen, and were stored at −80 °C until further analysis. Ground plant material was used for total RNA extraction, using the RNeasy Plant Mini Kit (Qiagen, Hilden, Germany), following the manufacturer’s instructions. The quantification of RNA and the evaluation of its purity were determined using a Quawell Q9000 micro spectrophotometer (Quawell Technology, Beijing, China), and RNA integrity was evaluated by denaturing agarose gel electrophoresis.

#### 4.1.3. Reverse Transcription PCR-Based Identification of ToBRFV

ToBRFV detection was carried out by RT-PCR using a set of primers targeting the small replicase subunit region, previously reported as specific for ToBRFV [48]. To exclude mixed infections, the composite symptomatic sample was tested for other common viruses in tomato, namely *Tomato mosaic virus* (ToMV), *Tobacco mosaic virus* (TMV) and *Tomato spotted wilt virus* (TSWV). *TUB* gene amplification was included as an internal positive control for cDNA quality and RT-PCR performance. Negative controls were added for all targets. Primer sequences are shown in Table 1.

For cDNA synthesis, 5000 ng of total RNA was reverse transcribed with the NZY First-Strand cDNA Synthesis Kit (NZYtech, Lisbon, Portugal), in 20 μL volume reactions, according to the manufacturer’s instructions.

The RT-PCR reaction was carried out in a 50 μL volume, containing 0.2 mM of dNTPs, 0.1 μM of each primer, 1 U of DreamTaq DNA polymerase (Thermo Fisher Scientific, Waltham, MA, USA), 5 μL of 10× Dream Taq buffer, and 3 μL of cDNA (≈750 ng). Cycling conditions consisted of an initial denaturation at 94 °C for 5 min, followed by 40 cycles of 30 s at 94 °C, 30 s at 53 °C and 1 min at 72 °C, and a final extension step for 10 min at 72 °C (MyCycler Thermal Cycler, Bio-Rad, Hercules, CA, USA).

The PCR products were analysed by electrophoresis on a 1.5% agarose gel stained with GreenSafe Premium (NZYtech, Lisboa, Portugal) and observed under a UV transilluminator and Gel Documentation System (SMART5, VWR International, Radnor, PA, USA). The GeneRuler 1 kb Plus DNA Ladder (Thermo Fisher Scientific, Waltham, MA, USA) was used to estimate the size of the amplified cDNA fragments after agarose gel electrophoresis.

### 4.2. Molecular Characterization of ToBRFV Portuguese Sequences

#### 4.2.1. Genome Sequencing, Sequence Analysis and Gene Annotation

Genome sequencing of the Portuguese ToBRFV-positive composite sample was performed using RNA-seq analysis. This analysis was outsourced to a commercial sequencing service (STAB Vida, Caparica, Portugal). Raw transcriptome sequence data were generated from total RNA through Next-generation Sequencing (lllumina Novaseq platform) of the previously synthesized cDNA molecules.

Primary processing of the raw sequence data, including quality control, trimming, and initial read mapping, was performed by STAB Vida (Caparica, Portugal) using the CLC Genomics Workbench 22.02 (Qiagen, Hilden, Germany). High-quality reads were filtered to remove those matching the tomato host genome, allowing the analysis to focus on non-host sequences potentially corresponding to plant-associated viruses. The filtered reads were subsequently mapped against a comprehensive plant virus database, based on NCBI Reference Sequences. The reference sequence used for ToBRFV was NC_028478.1, corresponding to Tom1-Jo (GenBank accession no.: KT383474).

Subsequently, the RNA-seq data provided by STAB Vida, specifically the reads matching the ToBRFV genome, were further analyzed using the CLC Genomics Workbench 24.0 (Qiagen, Hilden, Germany) to generate a consensus sequence and evaluate genome coverage. Nucleotide sequence analysis was then performed to identify the ORFs for gene annotation. All downstream analyses were performed using the high-quality filtered reads provided after host subtraction and viral mapping.

**Figure 8:**
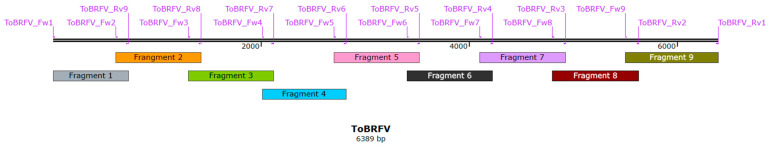
Diagram representing the location of each fragment and primers used along the ToBRFV genome. Figure generated in SnapGene Viewer (version 8.0.3).

Sanger sequencing was performed as an additional sequencing method on the same composite sample in order to independently validate RNA-seq-derived genome sequences. For amplification of the viral cDNA, nine pairs of primers were designed using SnapGene Viewer (version 8.0.3, GSL Biotech, San Diego, CA, USA), to generate overlapping amplicons spanning the complete ToBRFV genome (Figure 8). Primer binding sites were selected in conserved regions based on the reference sequence and aligned ToBRFV genomes, with the aim of maximizing amplification robustness and ensuring overlap between adjacent fragments to avoid loss of terminal sequence information and to facilitate accurate consensus reconstruction. Expected product sizes ranged between 720 and 889 bp. Primers were selected to minimize secondary structures, primer-dimer formation, and non-specific annealing. Primer sequences, expected product sizes, and annealing temperatures (Ta) used for RT-PCR are shown in Table 2. For clarity, primers are presented according to the overlapping amplicons generated across the genome, rather than by their original individual numbering. The RT-PCR reaction was carried out in a 50 μL volume, containing 25 μL of Q5^®^ High Fidelity 2× Master Mix, 0.5 μM of each primer, and 2 μL of cDNA. Primer sequences, expected product sizes, and annealing temperatures (Ta) used for RT-PCR are shown in Table 2. RT-PCR was performed with an initial denaturation step of 30 s at 98 °C, followed by 35 cycles of 10 s at 98 °C, 30 s at each Ta, and 30 s at 72 °C, with a final extension step for 2 min at 72 °C. A gradient PCR was employed to accommodate the different optimal Ta of each primer pair in a single run, as specified in Table 2. A negative control without template was included to monitor potential contamination.

PCR products were analysed by electrophoresis on a 1.5% agarose gel as described above. PCR products were purified using NZYGelpure (NZYtech, Lisbon, Portugal) according to the manufacturer’s instructions. The quantification and evaluation of PCR products’ purity were performed in a Quawell Q9000 micro spectrophotometer (Quawell Technology, Beijing, China). Sanger sequencing was outsourced to the commercial sequencing service of STAB Vida (Caparica, Portugal).

The results from Sanger sequencing were analyzed in the BioEdit Sequence Alignment Editor (version 7.2.5) [57], where the obtained fragments were used to generate a consensus sequence.

#### 4.2.2. Sequence Alignment and Comparative Analysis

In order to compare consensus sequences from RNA-seq analysis and Sanger sequencing analysis, both sequences were aligned using the BioEdit Sequence Alignment Editor (version 7.2.5) [57] and SNPs were noted. Amino acid sequences for each gene were generated and compared using MEGA (version 12) [58], to check for synonymous and non-synonymous SNPs.

The comparative analysis among sequences was performed using the NCBI Virus database (NCBI, Bethesda, MD, USA) [59]. All complete sequences of ToBRFV (taxid: 1761477) annotated as “nucleotide completeness” and available in the database as of 5 June 2025 were included. Sequence alignment was carried out using the MAFFT algorithm available within the NCBI Virus platform. Nucleotide differences between the Portuguese sequences, the reference sequence (NC_028478.1) and all other isolates were assessed, to check for synonymous and non-synonymous SNPs. Amino acid sequences for each protein gene were generated and compared using MEGA (version 12) [58], to determine if the viral proteins were affected.

#### 4.2.3. Functional and Structural Impact of Non-Synonymous Substitutions

Non-synonymous SNPs were further investigated to evaluate their potential functional and structural consequences. Preliminary predictions of functional impact were performed using SIFT [60], PolyPhen-2 [61] and MutPred2 [62], which were used to complement and cross-validate each other’s results. Three-dimensional protein modelling was performed using the Batch CD-Search tool (NCBI), which relies on the Conserved Domain Database (CDD) for annotation, to identify conserved domains, allowing the removal of non-domain regions prior to structure prediction [63,64]. Subsequently, the proteins of interest were subjected to three-dimensional structure prediction using AlphaFold3 server [65]. For each protein, the highest-confidence model was selected for further analysis. The predicted structures were visualized and edited using the PyMOL Molecular Graphics System (version 3.1; Schrödinger, LLC, New York, NY, USA). Structural validation was carried out using complementary approaches, including quality assessment with ProSA-web [66] (https://prosa.services.came.sbg.ac.at/prosa.php (accessed on 27 December 2025)), evaluation of structural consistency using QMEANDisCo [67] (https://swissmodel.expasy.org/qmean/ (accessed on 27 December 2025)), and stereochemical verification through Ramachandran plot analysis generated by PROCHECK (SAVES v6.1 Structure Validation Sever) [68] (https://www.ebi.ac.uk/thornton-srv/software/PROCHECK/ (accessed on 27 December 2025)).

### 4.3. Phylogenetic Analysis

Phylogenetic analysis was conducted using both nucleotide and amino acid sequences from all 300 ToBRFV isolates included in the study. For nucleotide-based phylogenetic inference, coding-complete genome sequences were used rather than full-length genomes including untranslated regions (UTRs). This approach was adopted because the UTRs showed insertion/deletion variation and length heterogeneity across isolates, which complicated positional homology and reduced the robustness of large-scale multiple-sequence alignment. The coding-complete dataset therefore provided a more reliable framework for comparative phylogenetic analysis across all retrieved sequences. For nucleotide-based analysis, the NCBI reference sequence of ToMV (GenBank accession no.: NC_002692.1) was added to the previously generated MAFFT alignment of ToBRFV isolates using the “add sequence to existing alignment” option in the advanced settings of MAFFT online service [69], in order to avoid any disruption of the original alignment. This analysis was performed considering only coding regions.

For amino acid-based analysis, the previously generated amino acid alignments for each protein-coding gene used in the comparative analysis among isolates were employed. Each protein was therefore analysed separately, and the ToMV reference sequence was also added to each corresponding MAFFT alignment using the “add sequence to existing alignment” option in the advanced settings of MAFFT online service [69], in order to avoid any disruption of the original alignment.

Phylogenetic relationships were inferred using the Maximum Likelihood method [22] implemented in MEGA (version 12) [58], under the best-fitting nucleotide and/or amino acid substitution models, with 1000 bootstrap replicates. Trees were rooted using ToMV as the outgroup. For better visualization and annotation, trees were edited using the Interactive Tree Of Life (iTOL) online tool (version 7.4.1) [70]. Only bootstrap support values higher than 65% were displayed at the nodes. Branch lengths were omitted to improve visualization, and major clades were identified and highlighted. Clade determination was restricted to groups supported by bootstrap values >65% and comprising more than three isolates.

Additionally, pairwise genetic distances were calculated in MEGA (version 12) [58], using the p-distance method with pairwise deletion of gaps, based on coding-complete nucleotide sequences. Distance values are summarized as mean, minimum, and maximum.

## 5. Conclusions

The present study provides the first molecularly validated confirmation and characterization of *Tomato brown rugose fruit virus* (ToBRFV) in Portugal and places the Portuguese sequences within a broader global context of ToBRFV genetic diversity. By combining RNA-seq with the cost-effective full-length genome sequencing Sanger technology, we generated independently validated consensus sequences and revealed intra-sample genomic variability, highlighting the added value of complementary sequencing approaches. Comprehensive genomic, functional, structural, and phylogenetic analysis revealed a low genetic variability, supporting the view that ToBRFV is a recently emerged virus that has spread rapidly across the globe. Notably, SNPs were mostly confined to the replicase-encoding proteins, which appear to tolerate these substitutions with no functional or structural effects, whereas the MP and CP were extremely conserved, reflecting strong purifying selection. The close phylogenetic relationship between Portuguese and non-European isolates, together with the inconclusive lineage assignment, underscores the limitations of phylogenetic inference for epidemiological tracing in highly conserved viruses and highlights the role of international trade in shaping current ToBRFV distribution patterns. Overall, these findings reinforce the importance of continuous surveillance, genomic monitoring, and integrative analytical approaches to accurately assess the spread, evolution, and impact of ToBRFV and to support informed risk assessment and effective management and control strategies. A limitation of the present study is that the genomic characterization was based on a single symptomatic composite field sample, which restricts conclusions regarding the broader diversity and prevalence of ToBRFV genotypes circulating in Portugal. Nevertheless, the use of two independent sequencing approaches provided technical support for the recovered genome sequence. Future work should include larger-scale sampling across regions, production systems, and epidemiological contexts to better capture the genomic diversity and transmission dynamics of ToBRFV in Portugal.

## Figures and Tables

**Table 1:** List of primers used for virus identification.

Primer Name/Target	Primer Sequence (5′-3′)	Product Size (bp)	Reference
ToBRFV1	F: AATGTCCATGTTTGTTACGCC R: CGAATGTGATTTAAAACTGTGAAT	560	[48]
ToMV	F: GTTTCATTGTGCTGTTGAGTAC R: AGAAGTACCCATATTGCTTCTTG	362	[54]
TMV	F: TGTGCAACACTCCGCRAAT R: CGGTTCGAGATCGAAAC	999	[54]
TSWV	F: ATGTCTAAGGTTAAGCTCACTA R: TTAAGCAAGTTCTGTGAGTTTT	777	[55]
*TUB*	F: CCTGGTGGTGACCTTGCTAAG R: CTCACCGACATACCAATGCAC	143	[56]

**Table 2:** List of primers used for total genome amplification in fragments.

Primer Pairs	Primer Sequence (5′-3′)	Product Size (bp)	Ta
ToBRFV Fw1 ToBRFV Rv9	GTATTTTTTACAACATATACCAAC TGCCGCTCCGAATTCATCAG	720	56 °C
ToBRFV Fw2 ToBRFV Rv8	GTTGTCTGTCACAGTACCTT ATTGCAAAAGAGATTTGTCAA	820	58.5 °C
ToBRFV Fw3 ToBRFV Rv7	AAGCCAAGGCACTTACATAC GTTGCCATGTGGAATTGCTCTA	820	62.4 °C
ToBRFV Fw4 ToBRFV Rv6	TTCAATGGCAAGAGGAGAGT CAACCAGTGTGCAACATCAG	807	63.8 °C
ToBRFV Fw5 ToBRFV Rv5	GCCACAAGAGATAATGTTCG ACTTATCATAGTAAAATTGC	820	52.2 °C
ToBRFV Fw6 ToBRFV Rv4	AAGTAGATGCAGGGACCCAA TCCAAGATATCCATAGGGAC	823	60.7 °C
ToBRFV Fw7 ToBRFV Rv3	CACAAGGCAAATGCTCGAAA TTACCCTTAACAAGAGCCAT	826	60.7 °C
ToBRFV Fw8 ToBRFV Rv2	GACGCTGTGAGTGAGGTCCA AACCCTTTTCCTTCCTTTGG	830	63.8 °C
ToBRFV Fw9 ToBRFV Rv1	GAAGTCCCGATGTCTGTAAG GGGCCCCTACCGGGGGTTC	889	62.4 °C

## Data Availability

DNA sequences generated in the present research were deposited in the NCBI GenBank database under the following accession numbers: PV978367.1 and PV978368.1.

## Supplementary Materials

The following supporting information can be downloaded at: https://www.mdpi.com/article/10.3390/plants15081240/s1, Table S1: Functional prediction of amino acid substitutions in the 183 kDa and 126 kDa replicase proteins; Table S2. Structural modelling validation results for the 183 kDa and 126 kDa replicases across four interest isolates; Data S1: Full alignment showing the consensus sequences resulting from RNA-seq analysis and Sanger sequencing; Figure S1: Ramachandran plot analysis for the 183 kDa replicase of the reference sequence; Figure S2: Ramachandran plot analysis for the 183 kDa replicase of the ToBRFV_PT1 isolate; Figure S3: Ramachandran plot analysis for the 183 kDa replicase of the ToBRFV_PT2 isolate; Figure S4: Ramachandran plot analysis for the 183 kDa replicase of the Israeli isolate; Figure S5: Ramachandran plot analysis for the 126 kDa replicase of the reference sequence; Figure S6: Ramachandran plot analysis for the 126 kDa replicase of the ToBRFV_PT1; Figure S7: Ramachandran plot analysis for the 126 kDa replicase of the ToBRFV_PT2; Figure S8: Ramachandran plot analysis for the 126 kDa replicase of the Israeli isolate. Figure S9: Maximum Likelihood phylogenetic tree inferred from the amino acid sequences of the 183 kDa replicase of ToBRFV isolates. Figure S10: Maximum Likelihood phylogenetic tree inferred from the amino acid sequences of the 126 kDa replicase of ToBRFV isolates. Figure S11: Maximum Likelihood phylogenetic tree inferred from the amino acid sequences of the Movement Protein of ToBRFV isolates. Figure S12: Maximum Likelihood phylogenetic tree inferred from the amino acid sequences of the Coat Protein of ToBRFV isolates.

## Abbreviations

The following abbreviations are used in this manuscript:

cDNA	Complementary DNA
CGMMV	*Cucumber green mottle mosaic virus*
CP	Coat protein
EPPO	European and Mediterranean Plant Protection Organization
GTR	General Time Reversible model
ICTV	International Committee on Taxonomy of Viruses
JTT	Jones–Taylor–Thornton model
ML	Maximum Likelihood
MP	Movement protein
NCBI	National Center for Biotechnology Information
NPPO	National Plant Protection Organization
nt	Nucleotide
ORFs	Open reading frames
PMMoV	*Pepper mild mottle virus*
RdRp	RNA-dependent RNA polymerase
RNA-seq	RNA sequencing
RT-PCR	Reverse Transcription Polymerase Chain Reaction
SNPs	Single nucleotide polymorphisms
ToBRFV	*Tomato brown rugose fruit virus*
TMV	*Tobacco mosaic virus*
ToMMV	*Tomato mottle mosaic virus*
ToMV	*Tomato mosaic virus*
TSWV	*Tomato spotted wilt virus*
UTR	Untranslated region
