# Supplementary material for: Molecular Characterization of Tomato Brown Rugose Fruit Virus in Portugal and Its Global Phylogenetic Context
Source: Plants (Basel). 2026 Apr 17;15(8):1240. doi: 10.3390/plants15081240 (PMC13120029; doi:10.3390/plants15081240)
Supplement: Supplementary file 1 [file plants-15-01240-s001.zip › plants-4222411-supplementary.pdf]

Supplementary Materials

Table S1. Functional prediction of amino acid substitutions in the 183 kDa and 126 kDa replicase proteins.

Protein	Input seq.	Mutation	SIFT			PolyPhen-2			MutPred score
			score	median	nr seq.	score	sensitivity	specificity	
183 kDa replicase	NC_028478.1 (NCBI ref. seq. Tom1-Jo)	L757S	0.74	3.05	12	0.000	1.00	0.00	0.374
		R979K	1.00	3.05	12	0.000	1.00	0.00	0.139
		D1368N	0.09	3.05	12	0.005	0.97	0.74	0.320
	OM_515237.1 (Israeli isolate)	L757S	0.74	3.05	12	0.000	1.00	0.00	0.385
		R979K	1.00	3.05	12	0.000	1.00	0.00	0.135
		H984Y	0.05	3.05	12	0.001	0.99	0.15	0.304
		D1368N	0.09	3.05	12	0.003	0.98	0.44	0.275
126 kDa replicase	NC_028478.1 (NCBI ref. seq. Tom1-Jo)	L757S	0.74	3.13	11	0.000	1.00	0.00	0.432
		R979K	0.77	3.13	11	0.000	1.00	0.00	0.125
	OM_515237.1 (Israeli isolate)	L757S	0.74	3.13	11	0.000	1.00	0.00	0.404
		R979K	0.77	3.13	11	0.000	1.00	0.00	0.137
		H984Y	0.08	3.13	11	0.0001	0.99	0.15	0.228

*SIFT scores are the normalized probability that the amino acid change is tolerated, scores ≤0.05 indicate potentially deleterious substitutions; SIFT median reflects the quality of the multiple sequence alignment and median values >3.00 indicate highly informative alignments and reliable predictions; SIFT number of sequences indicates the number of homologous sequences used and a higher number improves confidence in the predictions, usually >10 sequences are considered sufficient. PolyPhen-2 scores closer to 1 indicate a higher probability of damaging effects; PolyPhen-2 sensitivity is the ability of the model to correctly identify truly damaging mutations, values closer to 1 indicate very high sensitivity; PolyPhen-2 specificity is the ability to correctly identify truly benign mutations, values closer to 1 indicate very high specificity. MutPred2 scores ≥0.5 suggest potential functional disruption.

Table S2. Structural modelling validation results showing QMEANDisCo values, PROCHECK percentages and ProSA Z-scores for the 183 kDa and 126 kDa replicases across four interest isolates.

Protein	Isolate/Variant	QMEAN	PROCHECK (%)	ProSA (Z-scores)
183 kDa replicase	Ref. Seq.	0.58	92.3	-14.39
	ToBRFV_PT1	0.58	93.5	-13.72
	ToBRFV_PT2	0.60	93.1	-15.05
	Israeli	0.58	93.1	-14.42
126 kDa replicase	Ref. Seq.	0.63	92.6	-12.63
	ToBRFV_PT1	0.63	91.9	-12.13
	ToBRFV_PT2	0.63	90.2	-12.32
	Israeli	0.62	91.8	-11.64

Data S1. Full alignment showing the consensus sequences resulting from RNA-seq analysis (ToBRFV_PT1; GenBank accession number PV978367.1) and Sanger sequencing (ToBRFV_PT2; GenBank accession number PV978368.1). Sequences of ToBRFV fragments amplified, for each primer, to generate the consensus sequence are also presented. Nucleotide differences are highlighted in green.

```
             ....|....| ....|....| ....|....| ....|....| ....|....| ....|....|
                    10         20         30         40         50         60
TBRFV_PT1    GTATTTTTTA CAACATATAC CAACAACAAC AAACAACAAA CAACAACATT ACAATTACTA
TBRFV_PT2    -T-TTTTTTA CAACATATAC CAACAACAAC AAACAACAAA CAACAACATT ACAATTACTA
TBRFV Fw1    ---------- ---------- ---------- ---------- --ACAACATT ACAATTACTA
TBRFV Rv9    -T-TTTTTTA CAACATATAC CAACAACAAC AAACAACAAA CAACAACATT ACAATTACTA
TBRFV Fw2    ---------- ---------- ---------- ---------- ---------- ----------
TBRFV Rv8    ---------- ---------- ---------- ---------- ---------- ----------
TBRFV Fw3    ---------- ---------- ---------- ---------- ---------- ----------
TBRFV Rv7    ---------- ---------- ---------- ---------- ---------- ----------
TBRFV Fw4    ---------- ---------- ---------- ---------- ---------- ----------
TBRFV Rv6    ---------- ---------- ---------- ---------- ---------- ----------
TBRFV Fw5    ---------- ---------- ---------- ---------- ---------- ----------
TBRFV Rv5    ---------- ---------- ---------- ---------- ---------- ----------
TBRFV Fw6    ---------- ---------- ---------- ---------- ---------- ----------
TBRFV Rv4    ---------- ---------- ---------- ---------- ---------- ----------
TBRFV Fw7    ---------- ---------- ---------- ---------- ---------- ----------
TBRFV Rv3    ---------- ---------- ---------- ---------- ---------- ----------
TBRFV Fw8    ---------- ---------- ---------- ---------- ---------- ----------
TBRFV Rv2    ---------- ---------- ---------- ---------- ---------- ----------
TBRFV Fw9    ---------- ---------- ---------- ---------- ---------- ----------
TBRFV Rv1    ---------- ---------- ---------- ---------- ---------- ----------

             ....|....| ....|....| ....|....| ....|....| ....|....| ....|....|
                    70         80         90        100        110        120
TBRFV_PT1    TTTACAACTA CAATGGCATA CACACAGACA GCTACCACAT CCGCTTTGCT CGACACTGTC
TBRFV_PT2    TTTACAACTA CAATGGCATA CACACAGACA GCTACCACAT CCGCTTTGCT CGACACTGTC
TBRFV Fw1    TTTACAACTA CAATGGCATA CACACAGACA GCTACCACAT CCGCTTTGCT CGACACTGTC
TBRFV Rv9    TTTACAACTA CAATGGCATA CACACAGACA GCTACCACAT CCGCTTTGCT CGACACTGTC
TBRFV Fw2    ---------- ---------- ---------- ---------- ---------- ----------
TBRFV Rv8    ---------- ---------- ---------- ---------- ---------- ----------
TBRFV Fw3    ---------- ---------- ---------- ---------- ---------- ----------
TBRFV Rv7    ---------- ---------- ---------- ---------- ---------- ----------
TBRFV Fw4    ---------- ---------- ---------- ---------- ---------- ----------
TBRFV Rv6    ---------- ---------- ---------- ---------- ---------- ----------
TBRFV Fw5    ---------- ---------- ---------- ---------- ---------- ----------
TBRFV Rv5    ---------- ---------- ---------- ---------- ---------- ----------
TBRFV Fw6    ---------- ---------- ---------- ---------- ---------- ----------
TBRFV Rv4    ---------- ---------- ---------- ---------- ---------- ----------
TBRFV Fw7    ---------- ---------- ---------- ---------- ---------- ----------
TBRFV Rv3    ---------- ---------- ---------- ---------- ---------- ----------
TBRFV Fw8    ---------- ---------- ---------- ---------- ---------- ----------
TBRFV Rv2    ---------- ---------- ---------- ---------- ---------- ----------
TBRFV Fw9    ---------- ---------- ---------- ---------- ---------- ----------
TBRFV Rv1    ---------- ---------- ---------- ---------- ---------- ----------
```

```
               ....|....| ....|....| ....|....| ....|....| ....|....| ....|....|
                     130        140        150        160        170        180
TBRFV_PT1      CGAGGTAACA ATACCTTGGT CAACGATCTT GCGAAGCGGC GTCTTTATGA CACAGCGGTC
TBRFV_PT2      CGAGGTAACA ATACCTTGGT CAACGATCTT GCGAAGCGGC GTCTTTATGA CACAGCGGTC
TBRFV Fw1      CGAGGTAACA ATACCTTGGT CAACGATCTT GCGAAGCGGC GTCTTTATGA CACAGCGGTC
TBRFV Rv9      CGAGGTAACA ATACCTTGGT CAACGATCTT GCGAAGCGGC GTCTTTATGA CACAGCGGTC
TBRFV Fw2      ---------- ---------- ---------- ---------- ---------- ----------
TBRFV Rv8      ---------- ---------- ---------- ---------- ---------- ----------
TBRFV Fw3      ---------- ---------- ---------- ---------- ---------- ----------
TBRFV Rv7      ---------- ---------- ---------- ---------- ---------- ----------
TBRFV Fw4      ---------- ---------- ---------- ---------- ---------- ----------
TBRFV Rv6      ---------- ---------- ---------- ---------- ---------- ----------
TBRFV Fw5      ---------- ---------- ---------- ---------- ---------- ----------
TBRFV Rv5      ---------- ---------- ---------- ---------- ---------- ----------
TBRFV Fw6      ---------- ---------- ---------- ---------- ---------- ----------
TBRFV Rv4      ---------- ---------- ---------- ---------- ---------- ----------
TBRFV Fw7      ---------- ---------- ---------- ---------- ---------- ----------
TBRFV Rv3      ---------- ---------- ---------- ---------- ---------- ----------
TBRFV Fw8      ---------- ---------- ---------- ---------- ---------- ----------
TBRFV Rv2      ---------- ---------- ---------- ---------- ---------- ----------
TBRFV Fw9      ---------- ---------- ---------- ---------- ---------- ----------
TBRFV Rv1      ---------- ---------- ---------- ---------- ---------- ----------

               ....|....| ....|....| ....|....| ....|....| ....|....| ....|....|
                     190        200        210        220        230        240
TBRFV_PT1      GACGAGTTCA ACGCTCGTGA TCGCAGGCCC AAAGTAAATT TTTCCAAAGT AATAAGTGAG
TBRFV_PT2      GACGAGTTCA ACGCTCGTGA TCGCAGGCCC AAAGTAAATT TTTCCAAAGT AATAAGTGAG
TBRFV Fw1      GACGAGTTCA ACGCTCGTGA TCGCAGGCCC AAAGTAAATT TTTCCAAAGT AATAAGTGAG
TBRFV Rv9      GACGAGTTCA ACGCTCGTGA TCGCAGGCCC AAAGTAAATT TTTCCAAAGT AATAAGTGAG
TBRFV Fw2      ---------- ---------- ---------- ---------- ---------- ----------
TBRFV Rv8      ---------- ---------- ---------- ---------- ---------- ----------
TBRFV Fw3      ---------- ---------- ---------- ---------- ---------- ----------
TBRFV Rv7      ---------- ---------- ---------- ---------- ---------- ----------
TBRFV Fw4      ---------- ---------- ---------- ---------- ---------- ----------
TBRFV Rv6      ---------- ---------- ---------- ---------- ---------- ----------
TBRFV Fw5      ---------- ---------- ---------- ---------- ---------- ----------
TBRFV Rv5      ---------- ---------- ---------- ---------- ---------- ----------
TBRFV Fw6      ---------- ---------- ---------- ---------- ---------- ----------
TBRFV Rv4      ---------- ---------- ---------- ---------- ---------- ----------
TBRFV Fw7      ---------- ---------- ---------- ---------- ---------- ----------
TBRFV Rv3      ---------- ---------- ---------- ---------- ---------- ----------
TBRFV Fw8      ---------- ---------- ---------- ---------- ---------- ----------
TBRFV Rv2      ---------- ---------- ---------- ---------- ---------- ----------
TBRFV Fw9      ---------- ---------- ---------- ---------- ---------- ----------
TBRFV Rv1      ---------- ---------- ---------- ---------- ---------- ----------
```

```
               ....|....| ....|....| ....|....| ....|....| ....|....| ....|....|
                      250        260        270        280        290        300
TBRFV_PT1      GAACAGACGC TTATTGCTAC TAGGGCATAT CCAGAATTCC AGATAACCTT CTATAATACG
TBRFV_PT2      GAACAGACGC TTATTGCTAC TAGGGCATAT CCAGAATTCC AGATAACCTT CTATAATACG
TBRFV_Fw1      GAACAGACGC TTATTGCTAC TAGGGCATAT CCAGAATTCC AGATAACCTT CTATAATACG
TBRFV Rv9      GAACAGACGC TTATTGCTAC TAGGGCATAT CCAGAATTCC AGATAACCTT CTATAATACG
TBRFV Fw2      ---------- ---------- ---------- ---------- ---------- ----------
TBRFV Rv8      ---------- ---------- ---------- ---------- ---------- ----------
TBRFV Fw3      ---------- ---------- ---------- ---------- ---------- ----------
TBRFV Rv7      ---------- ---------- ---------- ---------- ---------- ----------
TBRFV Fw4      ---------- ---------- ---------- ---------- ---------- ----------
TBRFV Rv6      ---------- ---------- ---------- ---------- ---------- ----------
TBRFV Fw5      ---------- ---------- ---------- ---------- ---------- ----------
TBRFV Rv5      ---------- ---------- ---------- ---------- ---------- ----------
TBRFV Fw6      ---------- ---------- ---------- ---------- ---------- ----------
TBRFV Rv4      ---------- ---------- ---------- ---------- ---------- ----------
TBRFV Fw7      ---------- ---------- ---------- ---------- ---------- ----------
TBRFV Rv3      ---------- ---------- ---------- ---------- ---------- ----------
TBRFV Fw8      ---------- ---------- ---------- ---------- ---------- ----------
TBRFV Rv2      ---------- ---------- ---------- ---------- ---------- ----------
TBRFV Fw9      ---------- ---------- ---------- ---------- ---------- ----------
TBRFV Rv1      ---------- ---------- ---------- ---------- ---------- ----------

               ....|....| ....|....| ....|....| ....|....| ....|....| ....|....|
                      310        320        330        340        350        360
TBRFV_PT1      CAGAACGCCG TGCATTCGCT TGCCGGTGGA CTACGATCCT TAGAACTGGA ATATCTAATG
TBRFV_PT2      CAGAACGCCG TGCATTCGCT TGCCGGTGGA CTACGATCCT TAGAACTGGA ATATCTAATG
TBRFV_Fw1      CAGAACGCCG TGCATTCGCT TGCCGGTGGA CTACGATCCT TAGAACTGGA ATATCTAATG
TBRFV Rv9      CAGAACGCCG TGCATTCGCT TGCCGGTGGA CTACGATCCT TAGAACTGGA ATATCTAATG
TBRFV Fw2      ---------- ---------- ---------- ---------- ---------- ----------
TBRFV Rv8      ---------- ---------- ---------- ---------- ---------- ----------
TBRFV Fw3      ---------- ---------- ---------- ---------- ---------- ----------
TBRFV Rv7      ---------- ---------- ---------- ---------- ---------- ----------
TBRFV Fw4      ---------- ---------- ---------- ---------- ---------- ----------
TBRFV Rv6      ---------- ---------- ---------- ---------- ---------- ----------
TBRFV Fw5      ---------- ---------- ---------- ---------- ---------- ----------
TBRFV Rv5      ---------- ---------- ---------- ---------- ---------- ----------
TBRFV Fw6      ---------- ---------- ---------- ---------- ---------- ----------
TBRFV Rv4      ---------- ---------- ---------- ---------- ---------- ----------
TBRFV Fw7      ---------- ---------- ---------- ---------- ---------- ----------
TBRFV Rv3      ---------- ---------- ---------- ---------- ---------- ----------
TBRFV Fw8      ---------- ---------- ---------- ---------- ---------- ----------
TBRFV Rv2      ---------- ---------- ---------- ---------- ---------- ----------
TBRFV Fw9      ---------- ---------- ---------- ---------- ---------- ----------
TBRFV Rv1      ---------- ---------- ---------- ---------- ---------- ----------
```

```
               ....|....| ....|....| ....|....| ....|....| ....|....| ....|....|
                      370        380        390        400        410        420
TBRFV_PT1      ATGCAGATCC CGTACGGATC ACTCACATAT GATATAGGTG GGAATTTTGC ATCTCATCTG
TBRFV_PT2      ATGCAGATCC CGTACGGATC ACTCACATAT GATATAGGTG GGAATTTTGC ATCTCATCTG
TBRFV Fw1      ATGCAGATCC CGTACGGATC ACTCACATAT GATATAGGTG GGAATTTTGC ATCTCATCTG
TBRFV Rv9      ATGCAGATCC CGTACGGATC ACTCACATAT GATATAGGTG GGAATTTTGC ATCTCATCTG
TBRFV Fw2      ---------- ---------- ---------- ---------- ---------- ----------
TBRFV Rv8      ---------- ---------- ---------- ---------- ---------- ----------
TBRFV Fw3      ---------- ---------- ---------- ---------- ---------- ----------
TBRFV Rv7      ---------- ---------- ---------- ---------- ---------- ----------
TBRFV Fw4      ---------- ---------- ---------- ---------- ---------- ----------
TBRFV Rv6      ---------- ---------- ---------- ---------- ---------- ----------
TBRFV Fw5      ---------- ---------- ---------- ---------- ---------- ----------
TBRFV Rv5      ---------- ---------- ---------- ---------- ---------- ----------
TBRFV Fw6      ---------- ---------- ---------- ---------- ---------- ----------
TBRFV Rv4      ---------- ---------- ---------- ---------- ---------- ----------
TBRFV Fw7      ---------- ---------- ---------- ---------- ---------- ----------
TBRFV Rv3      ---------- ---------- ---------- ---------- ---------- ----------
TBRFV Fw8      ---------- ---------- ---------- ---------- ---------- ----------
TBRFV Rv2      ---------- ---------- ---------- ---------- ---------- ----------
TBRFV Fw9      ---------- ---------- ---------- ---------- ---------- ----------
TBRFV Rv1      ---------- ---------- ---------- ---------- ---------- ----------

               ....|....| ....|....| ....|....| ....|....| ....|....| ....|....|
                      430        440        450        460        470        480
TBRFV_PT1      TTCAAAGGAC GGGCATATGT TCACTGCTGT ATGCCCAATC TTGATGTCCG CGACATAATG
TBRFV_PT2      TTCAAAGGAC GGGCATATGT TCACTGCTGT ATGCCCAATC TTGATGTCCG CGACATAATG
TBRFV Fw1      TTCAAAGGAC GGGCATATGT TCACTGCTGT ATGCCCAATC TTGATGTCCG CGACATAATG
TBRFV Rv9      TTCAAAGGAC GGGCATATGT TCACTGCTGT ATGCCCAATC TTGATGTCCG CGACATAATG
TBRFV Fw2      ---------- ---------- ---------- ---------- ---------- ----------
TBRFV Rv8      ---------- ---------- ---------- ---------- ---------- ----------
TBRFV Fw3      ---------- ---------- ---------- ---------- ---------- ----------
TBRFV Rv7      ---------- ---------- ---------- ---------- ---------- ----------
TBRFV Fw4      ---------- ---------- ---------- ---------- ---------- ----------
TBRFV Rv6      ---------- ---------- ---------- ---------- ---------- ----------
TBRFV Fw5      ---------- ---------- ---------- ---------- ---------- ----------
TBRFV Rv5      ---------- ---------- ---------- ---------- ---------- ----------
TBRFV Fw6      ---------- ---------- ---------- ---------- ---------- ----------
TBRFV Rv4      ---------- ---------- ---------- ---------- ---------- ----------
TBRFV Fw7      ---------- ---------- ---------- ---------- ---------- ----------
TBRFV Rv3      ---------- ---------- ---------- ---------- ---------- ----------
TBRFV Fw8      ---------- ---------- ---------- ---------- ---------- ----------
TBRFV Rv2      ---------- ---------- ---------- ---------- ---------- ----------
TBRFV Fw9      ---------- ---------- ---------- ---------- ---------- ----------
TBRFV Rv1      ---------- ---------- ---------- ---------- ---------- ----------
```

```
                ....|....| ....|....| ....|....| ....|....| ....|....| ....|....|
                       490        500        510        520        530        540
TBRFV_PT1       CGGCACGAAG GCCAGAAAGA CAGTATAGAA TTATACCTTT CCAGGCTTGA GCGGGGCAAC
TBRFV_PT2       CGGCACGAAG GCCAGAAAGA CAGTATAGAA TTATACCTTT CCAGGCTTGA GCGGGGCAAC
TBRFV Fw1       CGGCACGAAG GCCAGAAAGA CAGTATAGAA TTATACCTTT CCAGGCTTGA GCGGGGCAAC
TBRFV Rv9       CGGCACGAAG GCCAGAAAGA CAGTATAGAA TTATACCTTT CCAGGCTTGA GCGGGGCAAC
TBRFV Fw2       ---------- ---------- ---------- ---------- ---------- ----------
TBRFV Rv8       ---------- ---------- ---------- ---------- ---------- ----------
TBRFV Fw3       ---------- ---------- ---------- ---------- ---------- ----------
TBRFV Rv7       ---------- ---------- ---------- ---------- ---------- ----------
TBRFV Fw4       ---------- ---------- ---------- ---------- ---------- ----------
TBRFV Rv6       ---------- ---------- ---------- ---------- ---------- ----------
TBRFV Fw5       ---------- ---------- ---------- ---------- ---------- ----------
TBRFV Rv5       ---------- ---------- ---------- ---------- ---------- ----------
TBRFV Fw6       ---------- ---------- ---------- ---------- ---------- ----------
TBRFV Rv4       ---------- ---------- ---------- ---------- ---------- ----------
TBRFV Fw7       ---------- ---------- ---------- ---------- ---------- ----------
TBRFV Rv3       ---------- ---------- ---------- ---------- ---------- ----------
TBRFV Fw8       ---------- ---------- ---------- ---------- ---------- ----------
TBRFV Rv2       ---------- ---------- ---------- ---------- ---------- ----------
TBRFV Fw9       ---------- ---------- ---------- ---------- ---------- ----------
TBRFV Rv1       ---------- ---------- ---------- ---------- ---------- ----------

                ....|....| ....|....| ....|....| ....|....| ....|....| ....|....|
                       550        560        570        580        590        600
TBRFV_PT1       AAAGTTGTCC CAAATTTCCA AAAGGAAGCT TTCGACAGAT ACGCTGAAAC GCCAGACGAA
TBRFV_PT2       AAAGTTGTCC CAAATTTCCA AAAGGAAGCT TTCGACAGAT ACGCTGAAAC GCCAGACGAA
TBRFV Fw1       AAAGTTGTCC CAAATTTCCA AAAGGAAGCT TTCGACAGAT ACGCTGAAAC GCCAGACGAA
TBRFV Rv9       AAAGTTGTCC CAAATTTCCA AAAGGAAGCT TTCGACAGAT ACGCTGAAAC GCCAGACGAA
TBRFV Fw2       ---------- ---------- ---------- ---------- ---------- ----------
TBRFV Rv8       ---------- ---------- ---------- ---------- ---------- ----------
TBRFV Fw3       ---------- ---------- ---------- ---------- ---------- ----------
TBRFV Rv7       ---------- ---------- ---------- ---------- ---------- ----------
TBRFV Fw4       ---------- ---------- ---------- ---------- ---------- ----------
TBRFV Rv6       ---------- ---------- ---------- ---------- ---------- ----------
TBRFV Fw5       ---------- ---------- ---------- ---------- ---------- ----------
TBRFV Rv5       ---------- ---------- ---------- ---------- ---------- ----------
TBRFV Fw6       ---------- ---------- ---------- ---------- ---------- ----------
TBRFV Rv4       ---------- ---------- ---------- ---------- ---------- ----------
TBRFV Fw7       ---------- ---------- ---------- ---------- ---------- ----------
TBRFV Rv3       ---------- ---------- ---------- ---------- ---------- ----------
TBRFV Fw8       ---------- ---------- ---------- ---------- ---------- ----------
TBRFV Rv2       ---------- ---------- ---------- ---------- ---------- ----------
TBRFV Fw9       ---------- ---------- ---------- ---------- ---------- ----------
TBRFV Rv1       ---------- ---------- ---------- ---------- ---------- ----------
```

```
               ....|....| ....|....| ....|....| ....|....| ....|....| ....|....|
                       610        620        630        640        650        660
TBRFV_PT1      GTTGTCTGTC ACAGTACCTT CCAAACGTGT ACGCACCAGC AGGTGGAAAA CACAGGCAGG
TBRFV_PT2      GTTGTCTGTC ACAGTACCTT CCAAACGTGT ACGCACCAGC AGGTGGAAAA CACAGGCAGG
TBRFV Fw1      GTTGTCTGTC ACAGTACCTT CCAAACGTGT ACGCACCAGC AGGTGGAAAA CACAGGCAGG
TBRFV Rv9      GTTGTCTGTC ACAGTACCTT CCAAACGTGT ACGCACCAGC AGGTGGAAAA CACAGGCAGG
TBRFV Fw2      ---------- ---------- ---------- ---------- ---------- ----------
TBRFV Rv8      ---------- ACAGTACCTT CCAAACGTGT ACGCACCAGC AGGTGGAAAA CACAGGCAGG
TBRFV Fw3      ---------- ---------- ---------- ---------- ---------- ----------
TBRFV Rv7      ---------- ---------- ---------- ---------- ---------- ----------
TBRFV Fw4      ---------- ---------- ---------- ---------- ---------- ----------
TBRFV Rv6      ---------- ---------- ---------- ---------- ---------- ----------
TBRFV Fw5      ---------- ---------- ---------- ---------- ---------- ----------
TBRFV Rv5      ---------- ---------- ---------- ---------- ---------- ----------
TBRFV Fw6      ---------- ---------- ---------- ---------- ---------- ----------
TBRFV Rv4      ---------- ---------- ---------- ---------- ---------- ----------
TBRFV Fw7      ---------- ---------- ---------- ---------- ---------- ----------
TBRFV Rv3      ---------- ---------- ---------- ---------- ---------- ----------
TBRFV Fw8      ---------- ---------- ---------- ---------- ---------- ----------
TBRFV Rv2      ---------- ---------- ---------- ---------- ---------- ----------
TBRFV Fw9      ---------- ---------- ---------- ---------- ---------- ----------
TBRFV Rv1      ---------- ---------- ---------- ---------- ---------- ----------

               ....|....| ....|....| ....|....| ....|....| ....|....| ....|....|
                       670        680        690        700        710        720
TBRFV_PT1      GTGTATGCTA TTGCATTGCA CAGTATATAC GATATACCTG CTGATGAATT CGGAGCGGCA
TBRFV_PT2      GTGTATGCTA TTGCATTGCA CAGTATATAC GATATACCTG CTGATGAATT CGGAGCGGCA
TBRFV Fw1      GTGTATGCTA TTGCATTGCA CAGTATATAC GATATACCTG CTGATGAATT CGGAGCGGCA
TBRFV Rv9      GTGTATGCTA T--------- ---------- ---------- ---------- ----------
TBRFV Fw2      -TGTATGCTA TTGCATTGCA CAGTATATAC GATATACCTG CTGATGAATT CGGAGCGGCA
TBRFV Rv8      GTGTATGCTA TTGCATTGCA CAGTATATAC GATATACCTG CTGATGAATT CGGAGCGGCA
TBRFV Fw3      ---------- ---------- ---------- ---------- ---------- ----------
TBRFV Rv7      ---------- ---------- ---------- ---------- ---------- ----------
TBRFV Fw4      ---------- ---------- ---------- ---------- ---------- ----------
TBRFV Rv6      ---------- ---------- ---------- ---------- ---------- ----------
TBRFV Fw5      ---------- ---------- ---------- ---------- ---------- ----------
TBRFV Rv5      ---------- ---------- ---------- ---------- ---------- ----------
TBRFV Fw6      ---------- ---------- ---------- ---------- ---------- ----------
TBRFV Rv4      ---------- ---------- ---------- ---------- ---------- ----------
TBRFV Fw7      ---------- ---------- ---------- ---------- ---------- ----------
TBRFV Rv3      ---------- ---------- ---------- ---------- ---------- ----------
TBRFV Fw8      ---------- ---------- ---------- ---------- ---------- ----------
TBRFV Rv2      ---------- ---------- ---------- ---------- ---------- ----------
TBRFV Fw9      ---------- ---------- ---------- ---------- ---------- ----------
TBRFV Rv1      ---------- ---------- ---------- ---------- ---------- ----------
```

```
                ....|....| ....|....| ....|....| ....|....| ....|....| ....|....|
                       730        740        750        760        770        780
TBRFV_PT1       CTTTTAAGGA AAAATGTCCA TGTTTGTTAC GCCGCCTTCC ACTTTTCCGA GAATTTACTT
TBRFV_PT2       CTTTTAAGGA AAAATGTCCA TGTTTGTTAC GCCGCCTTCC ACTTTTCCGA GAATTTACTT
TBRFV_Fw1       ---------- ---------- ---------- ---------- ---------- ----------
TBRFV Rv9       ---------- ---------- ---------- ---------- ---------- ----------
TBRFV Fw2       CTTTTAAGGA AAAATGTCCA TGTTTGTTAC GCCGCCTTCC ACTTTTCCGA GAATTTACTT
TBRFV Rv8       CTTTTAAGGA AAAATGTCCA TGTTTGTTAC GCCGCCTTCC ACTTTTCCGA GAATTTACTT
TBRFV Fw3       ---------- ---------- ---------- ---------- ---------- ----------
TBRFV Rv7       ---------- ---------- ---------- ---------- ---------- ----------
TBRFV Fw4       ---------- ---------- ---------- ---------- ---------- ----------
TBRFV Rv6       ---------- ---------- ---------- ---------- ---------- ----------
TBRFV Fw5       ---------- ---------- ---------- ---------- ---------- ----------
TBRFV Rv5       ---------- ---------- ---------- ---------- ---------- ----------
TBRFV Fw6       ---------- ---------- ---------- ---------- ---------- ----------
TBRFV Rv4       ---------- ---------- ---------- ---------- ---------- ----------
TBRFV Fw7       ---------- ---------- ---------- ---------- ---------- ----------
TBRFV Rv3       ---------- ---------- ---------- ---------- ---------- ----------
TBRFV Fw8       ---------- ---------- ---------- ---------- ---------- ----------
TBRFV Rv2       ---------- ---------- ---------- ---------- ---------- ----------
TBRFV Fw9       ---------- ---------- ---------- ---------- ---------- ----------
TBRFV Rv1       ---------- ---------- ---------- ---------- ---------- ----------

                ....|....| ....|....| ....|....| ....|....| ....|....| ....|....|
                       790        800        810        820        830        840
TBRFV_PT1       CTCGAAGATT CACACGTCAA CCTTGACGAA ATCAACGCGT GTTTTTCGCG TGATGGAGAC
TBRFV_PT2       CTCGAAGATT CACACGTCAA CCTTGACGAA ATCAACGCGT GTTTTTCGCG TGATGGAGAC
TBRFV_Fw1       ---------- ---------- ---------- ---------- ---------- ----------
TBRFV Rv9       ---------- ---------- ---------- ---------- ---------- ----------
TBRFV Fw2       CTCGAAGATT CACACGTCAA CCTTGACGAA ATCAACGCGT GTTTTTCGCG TGATGGAGAC
TBRFV Rv8       CTCGAAGATT CACACGTCAA CCTTGACGAA ATCAACGCGT GTTTTTCGCG TGATGGAGAC
TBRFV Fw3       ---------- ---------- ---------- ---------- ---------- ----------
TBRFV Rv7       ---------- ---------- ---------- ---------- ---------- ----------
TBRFV Fw4       ---------- ---------- ---------- ---------- ---------- ----------
TBRFV Rv6       ---------- ---------- ---------- ---------- ---------- ----------
TBRFV Fw5       ---------- ---------- ---------- ---------- ---------- ----------
TBRFV Rv5       ---------- ---------- ---------- ---------- ---------- ----------
TBRFV Fw6       ---------- ---------- ---------- ---------- ---------- ----------
TBRFV Rv4       ---------- ---------- ---------- ---------- ---------- ----------
TBRFV Fw7       ---------- ---------- ---------- ---------- ---------- ----------
TBRFV Rv3       ---------- ---------- ---------- ---------- ---------- ----------
TBRFV Fw8       ---------- ---------- ---------- ---------- ---------- ----------
TBRFV Rv2       ---------- ---------- ---------- ---------- ---------- ----------
TBRFV Fw9       ---------- ---------- ---------- ---------- ---------- ----------
TBRFV Rv1       ---------- ---------- ---------- ---------- ---------- ----------
```

```
                ....|....| ....|....| ....|....| ....|....| ....|....| ....|....|
                       850        860        870        880        890        900
TBRFV_PT1       AAGCTGACTT TTTCTTTCGC ATCTGAGAGC ACTTTAAATT ATTGTCATAG TTATTCTAAT
TBRFV_PT2       AAGCTGACTT TTTCTTTCGC ATCTGAGAGC ACTTTAAATT ATTGTCATAG TTATTCTAAT
TBRFV Fw1       ---------- ---------- ---------- ---------- ---------- ----------
TBRFV Rv9       ---------- ---------- ---------- ---------- ---------- ----------
TBRFV Fw2       AAGCTGACTT TTTCTTTCGC ATCTGAGAGC ACTTTAAATT ATTGTCATAG TTATTCTAAT
TBRFV Rv8       AAGCTGACTT TTTCTTTCGC ATCTGAGAGC ACTTTAAATT ATTGTCATAG TTATTCTAAT
TBRFV Fw3       ---------- ---------- ---------- ---------- ---------- ----------
TBRFV Rv7       ---------- ---------- ---------- ---------- ---------- ----------
TBRFV Fw4       ---------- ---------- ---------- ---------- ---------- ----------
TBRFV Rv6       ---------- ---------- ---------- ---------- ---------- ----------
TBRFV Fw5       ---------- ---------- ---------- ---------- ---------- ----------
TBRFV Rv5       ---------- ---------- ---------- ---------- ---------- ----------
TBRFV Fw6       ---------- ---------- ---------- ---------- ---------- ----------
TBRFV Rv4       ---------- ---------- ---------- ---------- ---------- ----------
TBRFV Fw7       ---------- ---------- ---------- ---------- ---------- ----------
TBRFV Rv3       ---------- ---------- ---------- ---------- ---------- ----------
TBRFV Fw8       ---------- ---------- ---------- ---------- ---------- ----------
TBRFV Rv2       ---------- ---------- ---------- ---------- ---------- ----------
TBRFV Fw9       ---------- ---------- ---------- ---------- ---------- ----------
TBRFV Rv1       ---------- ---------- ---------- ---------- ---------- ----------

                ....|....| ....|....| ....|....| ....|....| ....|....| ....|....|
                       910        920        930        940        950        960
TBRFV_PT1       ATTTTAAAAT ACGTGTGCAA AACTTACTTC CCGGCATCTA ATAGAGAGGT CTACATGAAG
TBRFV_PT2       ATTTTAAAAT ACGTGTGCAA AACTTACTTC CCGGCATCTA ATAGAGAGGT CTACATGAAG
TBRFV Fw1       ---------- ---------- ---------- ---------- ---------- ----------
TBRFV Rv9       ---------- ---------- ---------- ---------- ---------- ----------
TBRFV Fw2       ATTTTAAAAT ACGTGTGCAA AACTTACTTC CCGGCATCTA ATAGAGAGGT CTACATGAAG
TBRFV Rv8       ATTTTAAAAT ACGTGTGCAA AACTTACTTC CCGGCATCTA ATAGAGAGGT CTACATGAAG
TBRFV Fw3       ---------- ---------- ---------- ---------- ---------- ----------
TBRFV Rv7       ---------- ---------- ---------- ---------- ---------- ----------
TBRFV Fw4       ---------- ---------- ---------- ---------- ---------- ----------
TBRFV Rv6       ---------- ---------- ---------- ---------- ---------- ----------
TBRFV Fw5       ---------- ---------- ---------- ---------- ---------- ----------
TBRFV Rv5       ---------- ---------- ---------- ---------- ---------- ----------
TBRFV Fw6       ---------- ---------- ---------- ---------- ---------- ----------
TBRFV Rv4       ---------- ---------- ---------- ---------- ---------- ----------
TBRFV Fw7       ---------- ---------- ---------- ---------- ---------- ----------
TBRFV Rv3       ---------- ---------- ---------- ---------- ---------- ----------
TBRFV Fw8       ---------- ---------- ---------- ---------- ---------- ----------
TBRFV Rv2       ---------- ---------- ---------- ---------- ---------- ----------
TBRFV Fw9       ---------- ---------- ---------- ---------- ---------- ----------
TBRFV Rv1       ---------- ---------- ---------- ---------- ---------- ----------
```

```
                ....|....| ....|....| ....|....| ....|....| ....|....| ....|....|
                       970        980        990       1000       1010       1020
TBRFV_PT1       GAGTTTTTGG TCACCAGGGT TAACACCTGG TTTTGTAAGT TTTCTAGGAT AGATACTTTT
TBRFV_PT2       GAGTTTTTGG TCACCAGGGT TAACACCTGG TTTTGTAAGT TTTCTAGGAT AGATACTTTT
TBRFV Fw1       ---------- ---------- ---------- ---------- ---------- ----------
TBRFV Rv9       ---------- ---------- ---------- ---------- ---------- ----------
TBRFV Fw2       GAGTTTTTGG TCACCAGGGT TAACACCTGG TTTTGTAAGT TTTCTAGGAT AGATACTTTT
TBRFV Rv8       GAGTTTTTGG TCACCAGGGT TAACACCTGG TTTTGTAAGT TTTCTAGGAT AGATACTTTT
TBRFV Fw3       ---------- ---------- ---------- ---------- ---------- ----------
TBRFV Rv7       ---------- ---------- ---------- ---------- ---------- ----------
TBRFV Fw4       ---------- ---------- ---------- ---------- ---------- ----------
TBRFV Rv6       ---------- ---------- ---------- ---------- ---------- ----------
TBRFV Fw5       ---------- ---------- ---------- ---------- ---------- ----------
TBRFV Rv5       ---------- ---------- ---------- ---------- ---------- ----------
TBRFV Fw6       ---------- ---------- ---------- ---------- ---------- ----------
TBRFV Rv4       ---------- ---------- ---------- ---------- ---------- ----------
TBRFV Fw7       ---------- ---------- ---------- ---------- ---------- ----------
TBRFV Rv3       ---------- ---------- ---------- ---------- ---------- ----------
TBRFV Fw8       ---------- ---------- ---------- ---------- ---------- ----------
TBRFV Rv2       ---------- ---------- ---------- ---------- ---------- ----------
TBRFV Fw9       ---------- ---------- ---------- ---------- ---------- ----------
TBRFV Rv1       ---------- ---------- ---------- ---------- ---------- ----------

                ....|....| ....|....| ....|....| ....|....| ....|....| ....|....|
                      1030       1040       1050       1060       1070       1080
TBRFV_PT1       TTATTATACA AGGGGGTAGC CCACAAAGGT GTAAATAGTG AGCAATTTTA CAGCGCAATG
TBRFV_PT2       TTATTATACA AGGGGGTAGC CCACAAAGGT GTAAATAGTG AGCAATTTTA CAGCGCAATG
TBRFV Fw1       ---------- ---------- ---------- ---------- ---------- ----------
TBRFV Rv9       ---------- ---------- ---------- ---------- ---------- ----------
TBRFV Fw2       TTATTATACA AGGGGGTAGC CCACAAAGGT GTAAATAGTG AGCAATTTTA CAGCGCAATG
TBRFV Rv8       TTATTATACA AGGGGGTAGC CCACAAAGGT GTAAATAGTG AGCAATTTTA CAGCGCAATG
TBRFV Fw3       ---------- ---------- ---------- ---------- ---------- ----------
TBRFV Rv7       ---------- ---------- ---------- ---------- ---------- ----------
TBRFV Fw4       ---------- ---------- ---------- ---------- ---------- ----------
TBRFV Rv6       ---------- ---------- ---------- ---------- ---------- ----------
TBRFV Fw5       ---------- ---------- ---------- ---------- ---------- ----------
TBRFV Rv5       ---------- ---------- ---------- ---------- ---------- ----------
TBRFV Fw6       ---------- ---------- ---------- ---------- ---------- ----------
TBRFV Rv4       ---------- ---------- ---------- ---------- ---------- ----------
TBRFV Fw7       ---------- ---------- ---------- ---------- ---------- ----------
TBRFV Rv3       ---------- ---------- ---------- ---------- ---------- ----------
TBRFV Fw8       ---------- ---------- ---------- ---------- ---------- ----------
TBRFV Rv2       ---------- ---------- ---------- ---------- ---------- ----------
TBRFV Fw9       ---------- ---------- ---------- ---------- ---------- ----------
TBRFV Rv1       ---------- ---------- ---------- ---------- ---------- ----------
```

```
               ....|....| ....|....| ....|....| ....|....| ....|....| ....|....|
                      1090       1100       1110       1120       1130       1140
TBRFV_PT1      GAAGATGCAT GGCACTACAA AAAGACTCTT GCAATGTGTA ACAGCGAGAG GATTCTTCTC
TBRFV_PT2      GAAGATGCAT GGCACTACAA AAAGACTCTT GCAATGTGTA ACAGCGAGAG GATTCTTCTC
TBRFV Fw1      ---------- ---------- ---------- ---------- ---------- ----------
TBRFV Rv9      ---------- ---------- ---------- ---------- ---------- ----------
TBRFV Fw2      GAAGATGCAT GGCACTACAA AAAGACTCTT GCAATGTGTA ACAGCGAGAG GATTCTTCTC
TBRFV Rv8      GAAGATGCAT GGCACTACAA AAAGACTCTT GCAATGTGTA ACAGCGAGAG GATTCTTCTC
TBRFV Fw3      ---------- ---------- ---------- ---------- ---------- ----------
TBRFV Rv7      ---------- ---------- ---------- ---------- ---------- ----------
TBRFV Fw4      ---------- ---------- ---------- ---------- ---------- ----------
TBRFV Rv6      ---------- ---------- ---------- ---------- ---------- ----------
TBRFV Fw5      ---------- ---------- ---------- ---------- ---------- ----------
TBRFV Rv5      ---------- ---------- ---------- ---------- ---------- ----------
TBRFV Fw6      ---------- ---------- ---------- ---------- ---------- ----------
TBRFV Rv4      ---------- ---------- ---------- ---------- ---------- ----------
TBRFV Fw7      ---------- ---------- ---------- ---------- ---------- ----------
TBRFV Rv3      ---------- ---------- ---------- ---------- ---------- ----------
TBRFV Fw8      ---------- ---------- ---------- ---------- ---------- ----------
TBRFV Rv2      ---------- ---------- ---------- ---------- ---------- ----------
TBRFV Fw9      ---------- ---------- ---------- ---------- ---------- ----------
TBRFV Rv1      ---------- ---------- ---------- ---------- ---------- ----------

               ....|....| ....|....| ....|....| ....|....| ....|....| ....|....|
                      1150       1160       1170       1180       1190       1200
TBRFV_PT1      GAAGATTCCT CATCGGTCAA TTACTGGTTC CCAAAAATGA GAGATATGGT CATAGTTCCT
TBRFV_PT2      GAAGATTCCT CATCGGTCAA TTACTGGTTC CCAAAAATGA GAGATATGGT CATAGTTCCT
TBRFV Fw1      ---------- ---------- ---------- ---------- ---------- ----------
TBRFV Rv9      ---------- ---------- ---------- ---------- ---------- ----------
TBRFV Fw2      GAAGATTCCT CATCGGTCAA TTACTGGTTC CCAAAAATGA GAGATATGGT CATAGTTCCT
TBRFV Rv8      GAAGATTCCT CATCGGTCAA TTACTGGTTC CCAAAAATGA GAGATATGGT CATAGTTCCT
TBRFV Fw3      ---------- ---------- ---------- ---------- ---------- ----------
TBRFV Rv7      ---------- ---------- ---------- ---------- ---------- ----------
TBRFV Fw4      ---------- ---------- ---------- ---------- ---------- ----------
TBRFV Rv6      ---------- ---------- ---------- ---------- ---------- ----------
TBRFV Fw5      ---------- ---------- ---------- ---------- ---------- ----------
TBRFV Rv5      ---------- ---------- ---------- ---------- ---------- ----------
TBRFV Fw6      ---------- ---------- ---------- ---------- ---------- ----------
TBRFV Rv4      ---------- ---------- ---------- ---------- ---------- ----------
TBRFV Fw7      ---------- ---------- ---------- ---------- ---------- ----------
TBRFV Rv3      ---------- ---------- ---------- ---------- ---------- ----------
TBRFV Fw8      ---------- ---------- ---------- ---------- ---------- ----------
TBRFV Rv2      ---------- ---------- ---------- ---------- ---------- ----------
TBRFV Fw9      ---------- ---------- ---------- ---------- ---------- ----------
TBRFV Rv1      ---------- ---------- ---------- ---------- ---------- ----------
```

```
             ....|....| ....|....| ....|....| ....|....| ....|....| ....|....|
                     1210       1220       1230       1240       1250       126
TBRFV_PT1    CTATTCGACA TATCTCTCGA CACCAGTAAA AGGACCCGCA AAGAAGTCTT AGTGTCAAAG
TBRFV_PT2    CTATTCGACA TATCTCTCGA CACCAGTAAA AGGACCCGCA AAGAAGTCTT AGTGTCAAAG
TBRFV Fw1    ---------- ---------- ---------- ---------- ---------- ----------
TBRFV Rv9    ---------- ---------- ---------- ---------- ---------- ----------
TBRFV Fw2    CTATTCGACA TATCTCTCGA CACCAGTAAA AGGACCCGCA AAGAAGTCTT AGTGTCAAAG
TBRFV Rv8    CTATTCGACA TATCTCTCGA CACCAGTAAA AGGACCCGCA AAGAAGTCTT AGTGTCAAAG
TBRFV Fw3    ---------- ---------- ---------- ---------- ---------- ----------
TBRFV Rv7    ---------- ---------- ---------- ---------- ---------- ----------
TBRFV Fw4    ---------- ---------- ---------- ---------- ---------- ----------
TBRFV Rv6    ---------- ---------- ---------- ---------- ---------- ----------
TBRFV Fw5    ---------- ---------- ---------- ---------- ---------- ----------
TBRFV Rv5    ---------- ---------- ---------- ---------- ---------- ----------
TBRFV Fw6    ---------- ---------- ---------- ---------- ---------- ----------
TBRFV Rv4    ---------- ---------- ---------- ---------- ---------- ----------
TBRFV Fw7    ---------- ---------- ---------- ---------- ---------- ----------
TBRFV Rv3    ---------- ---------- ---------- ---------- ---------- ----------
TBRFV Fw8    ---------- ---------- ---------- ---------- ---------- ----------
TBRFV Rv2    ---------- ---------- ---------- ---------- ---------- ----------
TBRFV Fw9    ---------- ---------- ---------- ---------- ---------- ----------
TBRFV Rv1    ---------- ---------- ---------- ---------- ---------- ----------
```

```
             ....|....| ....|....| ....|....| ....|....| ....|....| ....|....|
                     1270       1280       1290       1300       1310       1320
TBRFV_PT1    GATTTTGTAT TCACAGTTTT AAATCACATT CGCACTTATC AAGCCAAGGC ACTTACATAC
TBRFV_PT2    GATTTTGTAT TCACAGTTTT AAATCACATT CGCACTTATC AAGCCAAGGC ACTTACATAC
TBRFV Fw1    ---------- ---------- ---------- ---------- ---------- ----------
TBRFV Rv9    ---------- ---------- ---------- ---------- ---------- ----------
TBRFV Fw2    GATTTTGTAT TCACAGTTTT AAATCACATT CGCACTTATC AAGCCAAGGC ACTTACATAC
TBRFV Rv8    GATTTTGTAT TCACAGTTTT AAATCACATT CGCACTTATC AAGCCAAGGC ACTTACATAC
TBRFV Fw3    ---------- ---------- ---------- ---------- ---------- ----------
TBRFV Rv7    ---------- ---------- ---------- ---------- ---------- ----------
TBRFV Fw4    ---------- ---------- ---------- ---------- ---------- ----------
TBRFV Rv6    ---------- ---------- ---------- ---------- ---------- ----------
TBRFV Fw5    ---------- ---------- ---------- ---------- ---------- ----------
TBRFV Rv5    ---------- ---------- ---------- ---------- ---------- ----------
TBRFV Fw6    ---------- ---------- ---------- ---------- ---------- ----------
TBRFV Rv4    ---------- ---------- ---------- ---------- ---------- ----------
TBRFV Fw7    ---------- ---------- ---------- ---------- ---------- ----------
TBRFV Rv3    ---------- ---------- ---------- ---------- ---------- ----------
TBRFV Fw8    ---------- ---------- ---------- ---------- ---------- ----------
TBRFV Rv2    ---------- ---------- ---------- ---------- ---------- ----------
TBRFV Fw9    ---------- ---------- ---------- ---------- ---------- ----------
TBRFV Rv1    ---------- ---------- ---------- ---------- ---------- ----------
```

```
             ....|....| ....|....| ....|....| ....|....| ....|....| ....|....|
                   1330       1340       1350       1360       1370       1380
TBRFV_PT1    TCCAATGTTT TATCCTTTGT CGAATCAATT CGTTCAAGGG TAATTATCAA CGGAGTGACT
TBRFV_PT2    TCCAATGTTT TATCCTTTGT CGAATCAATT CGTTCAAGGG TAATTATCAA CGGAGTGACT
TBRFV_Fw1    ---------- ---------- ---------- ---------- ---------- ----------
TBRFV Rv9    ---------- ---------- ---------- ---------- ---------- ----------
TBRFV Fw2    TCCAATGTTT TATCCTTTGT CGAATCAATT CGTTCAAGGG TAATTATCAA CGGAGTGACT
TBRFV Rv8    TCCAATGTTT TATCCTTTGT CGAATCAATT CGTTCAAGGG TAATTATCA- ----------
TBRFV Fw3    ---------- ---------- --------TT CGTTCAAGGG TAATTATCAA CGGAGTGACT
TBRFV Rv7    TCCAATGTTT TATCCTTTGT CGAATCAATT CGTTCAAGGG TAATTATCAA CGGAGTGACT
TBRFV Fw4    ---------- ---------- ---------- ---------- ---------- ----------
TBRFV Rv6    ---------- ---------- ---------- ---------- ---------- ----------
TBRFV Fw5    ---------- ---------- ---------- ---------- ---------- ----------
TBRFV Rv5    ---------- ---------- ---------- ---------- ---------- ----------
TBRFV Fw6    ---------- ---------- ---------- ---------- ---------- ----------
TBRFV Rv4    ---------- ---------- ---------- ---------- ---------- ----------
TBRFV Fw7    ---------- ---------- ---------- ---------- ---------- ----------
TBRFV Rv3    ---------- ---------- ---------- ---------- ---------- ----------
TBRFV Fw8    ---------- ---------- ---------- ---------- ---------- ----------
TBRFV Rv2    ---------- ---------- ---------- ---------- ---------- ----------
TBRFV Fw9    ---------- ---------- ---------- ---------- ---------- ----------
TBRFV Rv1    ---------- ---------- ---------- ---------- ---------- ----------
```

```
             ....|....| ....|....| ....|....| ....|....| ....|....| ....|....|
                   1390       1400       1410       1420       1430       1440
TBRFV_PT1    GCCAGGTCTG AGTGGGATGT TGACAAATCT CTTTTGCAAT CCTTGTCCAT GACATTTTTC
TBRFV_PT2    GCCAGGTCTG AGTGGGATGT TGACAAATCT CTTTTGCAAT CCTTGTCCAT GACATTTTTC
TBRFV_Fw1    ---------- ---------- ---------- ---------- ---------- ----------
TBRFV Rv9    ---------- ---------- ---------- ---------- ---------- ----------
TBRFV Fw2    GCCAGGTCTG AGTGGGATGT TGAC------ ---------- ---------- ----------
TBRFV Rv8    ---------- ---------- ---------- ---------- ---------- ----------
TBRFV Fw3    GCCAGGTCTG AGTGGGATGT TGACAAATCT CTTTTGCAAT CCTTGTCCAT GACATTTTTC
TBRFV Rv7    GCCAGGTCTG AGTGGGATGT TGACAAATCT CTTTTGCAAT CCTTGTCCAT GACATTTTTC
TBRFV Fw4    ---------- ---------- ---------- ---------- ---------- ----------
TBRFV Rv6    ---------- ---------- ---------- ---------- ---------- ----------
TBRFV Fw5    ---------- ---------- ---------- ---------- ---------- ----------
TBRFV Rv5    ---------- ---------- ---------- ---------- ---------- ----------
TBRFV Fw6    ---------- ---------- ---------- ---------- ---------- ----------
TBRFV Rv4    ---------- ---------- ---------- ---------- ---------- ----------
TBRFV Fw7    ---------- ---------- ---------- ---------- ---------- ----------
TBRFV Rv3    ---------- ---------- ---------- ---------- ---------- ----------
TBRFV Fw8    ---------- ---------- ---------- ---------- ---------- ----------
TBRFV Rv2    ---------- ---------- ---------- ---------- ---------- ----------
TBRFV Fw9    ---------- ---------- ---------- ---------- ---------- ----------
TBRFV Rv1    ---------- ---------- ---------- ---------- ---------- ----------
```

```
               ....|....| ....|....| ....|....| ....|....| ....|....| ....|....|
                       1450       1460       1470       1480       1490       1500
TBRFV_PT1      TTGCATACTA AGCTTGCCGT TTTAAAAGAC GAATTGTTAA TCAGCAAGTT TAGTTTGGGG
TBRFV_PT2      TTGCATACTA AGCTTGCCGT TTTAAAAGAC GAATTGTTAA TCAGCAAGTT TAGTTTGGGG
TBRFV Fw1      ---------- ---------- ---------- ---------- ---------- ----------
TBRFV Rv9      ---------- ---------- ---------- ---------- ---------- ----------
TBRFV Fw2      ---------- ---------- ---------- ---------- ---------- ----------
TBRFV Rv8      ---------- ---------- ---------- ---------- ---------- ----------
TBRFV Fw3      TTGCATACTA AGCTTGCCGT TTTAAAAGAC GAATTGTTAA TCAGCAAGTT TAGTTTGGGG
TBRFV Rv7      TTGCATACTA AGCTTGCCGT TTTAAAAGAC GAATTGTTAA TCAGCAAGTT TAGTTTGGGG
TBRFV Fw4      ---------- ---------- ---------- ---------- ---------- ----------
TBRFV Rv6      ---------- ---------- ---------- ---------- ---------- ----------
TBRFV Fw5      ---------- ---------- ---------- ---------- ---------- ----------
TBRFV Rv5      ---------- ---------- ---------- ---------- ---------- ----------
TBRFV Fw6      ---------- ---------- ---------- ---------- ---------- ----------
TBRFV Rv4      ---------- ---------- ---------- ---------- ---------- ----------
TBRFV Fw7      ---------- ---------- ---------- ---------- ---------- ----------
TBRFV Rv3      ---------- ---------- ---------- ---------- ---------- ----------
TBRFV Fw8      ---------- ---------- ---------- ---------- ---------- ----------
TBRFV Rv2      ---------- ---------- ---------- ---------- ---------- ----------
TBRFV Fw9      ---------- ---------- ---------- ---------- ---------- ----------
TBRFV Rv1      ---------- ---------- ---------- ---------- ---------- ----------
```

```
               ....|....| ....|....| ....|....| ....|....| ....|....| ....|....|
                       1510       1520       1530       1540       1550       1560
TBRFV_PT1      CCAAAATCAG TAAGCCAGCA TGTATGGGAT GAGATTTCCC TGGCTTTTGG AAACGCATTT
TBRFV_PT2      CCAAAATCAG TAAGCCAGCA TGTATGGGAT GAGATTTCCC TGGCTTTTGG AAACGCATTT
TBRFV Fw1      ---------- ---------- ---------- ---------- ---------- ----------
TBRFV Rv9      ---------- ---------- ---------- ---------- ---------- ----------
TBRFV Fw2      ---------- ---------- ---------- ---------- ---------- ----------
TBRFV Rv8      ---------- ---------- ---------- ---------- ---------- ----------
TBRFV Fw3      CCAAAATCAG TAAGCCAGCA TGTATGGGAT GAGATTTCCC TGGCTTTTGG AAACGCATTT
TBRFV Rv7      CCAAAATCAG TAAGCCAGCA TGTATGGGAT GAGATTTCCC TGGCTTTTGG AAACGCATTT
TBRFV Fw4      ---------- ---------- ---------- ---------- ---------- ----------
TBRFV Rv6      ---------- ---------- ---------- ---------- ---------- ----------
TBRFV Fw5      ---------- ---------- ---------- ---------- ---------- ----------
TBRFV Rv5      ---------- ---------- ---------- ---------- ---------- ----------
TBRFV Fw6      ---------- ---------- ---------- ---------- ---------- ----------
TBRFV Rv4      ---------- ---------- ---------- ---------- ---------- ----------
TBRFV Fw7      ---------- ---------- ---------- ---------- ---------- ----------
TBRFV Rv3      ---------- ---------- ---------- ---------- ---------- ----------
TBRFV Fw8      ---------- ---------- ---------- ---------- ---------- ----------
TBRFV Rv2      ---------- ---------- ---------- ---------- ---------- ----------
TBRFV Fw9      ---------- ---------- ---------- ---------- ---------- ----------
TBRFV Rv1      ---------- ---------- ---------- ---------- ---------- ----------
```

```
               ....|....| ....|....| ....|....| ....|....| ....|....| ....|....|
                       1570       1580       1590       1600       1610       1620
TBRFV_PT1      CCATCGATCA AGGAGAGACT GCTAAATCGG AAACTAATTA AAGTGTCGGG AGACGCATTA
TBRFV_PT2      CCATCGATCA AGGAGAGACT GCTAAATCGG AAACTAATTA AAGTGTCGGG AGACGCATTA
TBRFV Fw1      ---------- ---------- ---------- ---------- ---------- ----------
TBRFV Rv9      ---------- ---------- ---------- ---------- ---------- ----------
TBRFV Fw2      ---------- ---------- ---------- ---------- ---------- ----------
TBRFV Rv8      ---------- ---------- ---------- ---------- ---------- ----------
TBRFV Fw3      CCATCGATCA AGGAGAGACT GCTAAATCGG AAACTAATTA AAGTGTCGGG AGACGCATTA
TBRFV Rv7      CCATCGATCA AGGAGAGACT GCTAAATCGG AAACTAATTA AAGTGTCGGG AGACGCATTA
```

```
TBRFV Fw4      ---------- ---------- ---------- ---------- ---------- ----------
TBRFV Rv6      ---------- ---------- ---------- ---------- ---------- ----------
TBRFV Fw5      ---------- ---------- ---------- ---------- ---------- ----------
TBRFV Rv5      ---------- ---------- ---------- ---------- ---------- ----------
TBRFV Fw6      ---------- ---------- ---------- ---------- ---------- ----------
TBRFV Rv4      ---------- ---------- ---------- ---------- ---------- ----------
TBRFV Fw7      ---------- ---------- ---------- ---------- ---------- ----------
TBRFV Rv3      ---------- ---------- ---------- ---------- ---------- ----------
TBRFV Fw8      ---------- ---------- ---------- ---------- ---------- ----------
TBRFV Rv2      ---------- ---------- ---------- ---------- ---------- ----------
TBRFV Fw9      ---------- ---------- ---------- ---------- ---------- ----------
TBRFV Rv1      ---------- ---------- ---------- ---------- ---------- ----------

               ....|....| ....|....| ....|....| ....|....| ....|....| ....|....|
                     1630       1640       1650       1660       1670       1680
TBRFV_PT1      GAAATCAGGG TGCCTGATTT ATATGTGACT TTTCACGATA GATTAGTGAC TGAGTACAAA
TBRFV_PT2      GAAATCAGGG TGCCTGATTT ATATGTGACT TTTCACGATA GATTAGTGAC TGAGTACAAA
TBRFV Fw1      ---------- ---------- ---------- ---------- ---------- ----------
TBRFV Rv9      ---------- ---------- ---------- ---------- ---------- ----------
TBRFV Fw2      ---------- ---------- ---------- ---------- ---------- ----------
TBRFV Rv8      ---------- ---------- ---------- ---------- ---------- ----------
TBRFV Fw3      GAAATCAGGG TGCCTGATTT ATATGTGACT TTTCACGATA GATTAGTGAC TGAGTACAAA
TBRFV Rv7      GAAATCAGGG TGCCTGATTT ATATGTGACT TTTCACGATA GATTAGTGAC TGAGTACAAA
TBRFV Fw4      ---------- ---------- ---------- ---------- ---------- ----------
TBRFV Rv6      ---------- ---------- ---------- ---------- ---------- ----------
TBRFV Fw5      ---------- ---------- ---------- ---------- ---------- ----------
TBRFV Rv5      ---------- ---------- ---------- ---------- ---------- ----------
TBRFV Fw6      ---------- ---------- ---------- ---------- ---------- ----------
TBRFV Rv4      ---------- ---------- ---------- ---------- ---------- ----------
TBRFV Fw7      ---------- ---------- ---------- ---------- ---------- ----------
TBRFV Rv3      ---------- ---------- ---------- ---------- ---------- ----------
TBRFV Fw8      ---------- ---------- ---------- ---------- ---------- ----------
TBRFV Rv2      ---------- ---------- ---------- ---------- ---------- ----------
TBRFV Fw9      ---------- ---------- ---------- ---------- ---------- ----------
TBRFV Rv1      ---------- ---------- ---------- ---------- ---------- ----------
```

```
               ....|....| ....|....| ....|....| ....|....| ....|....| ....|....|
                      1690       1700       1710       1720       1730       1740
TBRFV_PT1      ACATCGGTGG ATATGCCAGT GCTTGATATC AGAAAGAGAA TGGAGGAGAC TGAGGTTATG
TBRFV_PT2      ACATCGGTGG ATATGCCAGT GCTTGATATC AGAAAGAGAA TGGAGGAGAC TGAGGTTATG
TBRFV Fw1      ---------- ---------- ---------- ---------- ---------- ----------
TBRFV Rv9      ---------- ---------- ---------- ---------- ---------- ----------
TBRFV Fw2      ---------- ---------- ---------- ---------- ---------- ----------
TBRFV Rv8      ---------- ---------- ---------- ---------- ---------- ----------
TBRFV Fw3      ACATCGGTGG ATATGCCAGT GCTTGATATC AGAAAGAGAA TGGAGGAGAC TGAGGTTATG
TBRFV Rv7      ACATCGGTGG ATATGCCAGT GCTTGATATC AGAAAGAGAA TGGAGGAGAC TGAGGTTATG
TBRFV Fw4      ---------- ---------- ---------- ---------- ---------- ----------
TBRFV Rv6      ---------- ---------- ---------- ---------- ---------- ----------
TBRFV Fw5      ---------- ---------- ---------- ---------- ---------- ----------
TBRFV Rv5      ---------- ---------- ---------- ---------- ---------- ----------
TBRFV Fw6      ---------- ---------- ---------- ---------- ---------- ----------
TBRFV Rv4      ---------- ---------- ---------- ---------- ---------- ----------
TBRFV Fw7      ---------- ---------- ---------- ---------- ---------- ----------
TBRFV Rv3      ---------- ---------- ---------- ---------- ---------- ----------
TBRFV Fw8      ---------- ---------- ---------- ---------- ---------- ----------
TBRFV Rv2      ---------- ---------- ---------- ---------- ---------- ----------
TBRFV Fw9      ---------- ---------- ---------- ---------- ---------- ----------
TBRFV Rv1      ---------- ---------- ---------- ---------- ---------- ----------
```

```
               ....|....| ....|....| ....|....| ....|....| ....|....| ....|....|
                      1750       1760       1770       1780       1790       1800
TBRFV_PT1      TACAATGCAT TGTCTGAGCT ATCTGTGCTC AAGGAGTCGG ACAAGTTCGA CGTTGATGTT
TBRFV_PT2      TACAATGCAT TGTCTGAGCT ATCTGTGCTC AAGGAGTCGG ACAAGTTCGA CGTTGATGTT
TBRFV Fw1      ---------- ---------- ---------- ---------- ---------- ----------
TBRFV Rv9      ---------- ---------- ---------- ---------- ---------- ----------
TBRFV Fw2      ---------- ---------- ---------- ---------- ---------- ----------
TBRFV Rv8      ---------- ---------- ---------- ---------- ---------- ----------
TBRFV Fw3      TACAATGCAT TGTCTGAGCT ATCTGTGCTC AAGGAGTCGG ACAAGTTCGA CGTTGATGTT
TBRFV Rv7      TACAATGCAT TGTCTGAGCT ATCTGTGCTC AAGGAGTCGG ACAAGTTCGA CGTTGATGTT
TBRFV Fw4      ---------- ---------- ---------- ---------- ---------- ----------
TBRFV Rv6      ---------- ---------- ---------- ---------- ---------- ----------
TBRFV Fw5      ---------- ---------- ---------- ---------- ---------- ----------
TBRFV Rv5      ---------- ---------- ---------- ---------- ---------- ----------
TBRFV Fw6      ---------- ---------- ---------- ---------- ---------- ----------
TBRFV Rv4      ---------- ---------- ---------- ---------- ---------- ----------
TBRFV Fw7      ---------- ---------- ---------- ---------- ---------- ----------
TBRFV Rv3      ---------- ---------- ---------- ---------- ---------- ----------
TBRFV Fw8      ---------- ---------- ---------- ---------- ---------- ----------
TBRFV Rv2      ---------- ---------- ---------- ---------- ---------- ----------
TBRFV Fw9      ---------- ---------- ---------- ---------- ---------- ----------
TBRFV Rv1      ---------- ---------- ---------- ---------- ---------- ----------
```

```
              ....|....| ....|....| ....|....| ....|....| ....|....| ....|....|
                    1810       1820       1830       1840       1850       1860
TBRFV_PT1     TTTTCCCGGA TGTGCCAGAC TTTGGAGGTA GACCCAATGA CTGCAGCAAA GGTTATTGTG
TBRFV_PT2     TTTTCCCGGA TGTGCCAGAC TTTGGAGGTA GACCCAATGA CTGCAGCAAA GGTTATTGTG
TBRFV Fw1     ---------- ---------- ---------- ---------- ---------- ----------
TBRFV Rv9     ---------- ---------- ---------- ---------- ---------- ----------
TBRFV Fw2     ---------- ---------- ---------- ---------- ---------- ----------
TBRFV Rv8     ---------- ---------- ---------- ---------- ---------- ----------
TBRFV Fw3     TTTTCCCGGA TGTGCCAGAC TTTGGAGGTA GACCCAATGA CTGCAGCAAA GGTTATTGTG
TBRFV Rv7     TTTTCCCGGA TGTGCCAGAC TTTGGAGGTA GACCCAATGA CTGCAGCAAA GGTTATTGTG
TBRFV Fw4     ---------- ---------- ---------- ---------- ---------- ----------
TBRFV Rv6     ---------- ---------- ---------- ---------- ---------- ----------
TBRFV Fw5     ---------- ---------- ---------- ---------- ---------- ----------
TBRFV Rv5     ---------- ---------- ---------- ---------- ---------- ----------
TBRFV Fw6     ---------- ---------- ---------- ---------- ---------- ----------
TBRFV Rv4     ---------- ---------- ---------- ---------- ---------- ----------
TBRFV Fw7     ---------- ---------- ---------- ---------- ---------- ----------
TBRFV Rv3     ---------- ---------- ---------- ---------- ---------- ----------
TBRFV Fw8     ---------- ---------- ---------- ---------- ---------- ----------
TBRFV Rv2     ---------- ---------- ---------- ---------- ---------- ----------
TBRFV Fw9     ---------- ---------- ---------- ---------- ---------- ----------
TBRFV Rv1     ---------- ---------- ---------- ---------- ---------- ----------
```

```
              ....|....| ....|....| ....|....| ....|....| ....|....| ....|....|
                    1870       1880       1890       1900       1910       1920
TBRFV_PT1     GCAGTGATGA GCAATGAGAG CGGACTGACT CTTACATTCG AACAGCCAAC TGAAGCAAAT
TBRFV_PT2     GCAGTGATGA GCAATGAGAG CGGACTGACT CTTACATTCG AACAGCCAAC TGAAGCAAAT
TBRFV Fw1     ---------- ---------- ---------- ---------- ---------- ----------
TBRFV Rv9     ---------- ---------- ---------- ---------- ---------- ----------
TBRFV Fw2     ---------- ---------- ---------- ---------- ---------- ----------
TBRFV Rv8     ---------- ---------- ---------- ---------- ---------- ----------
TBRFV Fw3     GCAGTGATGA GCAATGAGAG CGGACTGACT CTTACATTCG AACAGCCAAC TGAAGCAAAT
TBRFV Rv7     GCAGTGATGA GCAATGAGAG CGGACTGACT CTTACATTCG AACAGCCAAC TGAAGCAAAT
TBRFV Fw4     ---------- ---------- ---------- ---------- ---------- ----------
TBRFV Rv6     ---------- ---------- ---------- ---------- ---------- ----------
TBRFV Fw5     ---------- ---------- ---------- ---------- ---------- ----------
TBRFV Rv5     ---------- ---------- ---------- ---------- ---------- ----------
TBRFV Fw6     ---------- ---------- ---------- ---------- ---------- ----------
TBRFV Rv4     ---------- ---------- ---------- ---------- ---------- ----------
TBRFV Fw7     ---------- ---------- ---------- ---------- ---------- ----------
TBRFV Rv3     ---------- ---------- ---------- ---------- ---------- ----------
TBRFV Fw8     ---------- ---------- ---------- ---------- ---------- ----------
TBRFV Rv2     ---------- ---------- ---------- ---------- ---------- ----------
TBRFV Fw9     ---------- ---------- ---------- ---------- ---------- ----------
TBRFV Rv1     ---------- ---------- ---------- ---------- ---------- ----------
```

```
              ....|....| ....|....| ....|....| ....|....| ....|....| ....|....|
                    1930       1940       1950       1960       1970       1980
TBRFV_PT1     GTCGCATTGG CACTTAAAGA TTCAGAAAAA GCCTCTGAGG GTGCACTAGT GGTTACTTCT
TBRFV_PT2     GTCGCATTGG CACTTAAAGA TTCAGAAAAA GCCTCTGAGG GTGCACTAGT GGTTACTTCT
TBRFV Fw1     ---------- ---------- ---------- ---------- ---------- ----------
TBRFV Rv9     ---------- ---------- ---------- ---------- ---------- ----------
TBRFV Fw2     ---------- ---------- ---------- ---------- ---------- ----------
TBRFV Rv8     ---------- ---------- ---------- ---------- ---------- ----------
```

https://doi.org/10.3390/plants15081240

```
TBRFV Fw3   GTCGCATTGG CACTTAAAGA TTCAGAAAAA GCCTCTGAGG GTGCACTAGT GGTTACTTCT
TBRFV Rv7   GTCGCATTGG CACTTAAAGA TTCAGAAAAA GCCTCTGAGG GTGCACTAGT GGTTACTTCT
TBRFV Fw4   ---------- ---------- ---------- ---------- ---------- ----------
TBRFV Rv6   ---------- ---------- ---------- ---------- ---------- ----------
TBRFV Fw5   ---------- ---------- ---------- ---------- ---------- ----------
TBRFV Rv5   ---------- ---------- ---------- ---------- ---------- ----------
TBRFV Fw6   ---------- ---------- ---------- ---------- ---------- ----------
TBRFV Rv4   ---------- ---------- ---------- ---------- ---------- ----------
TBRFV Fw7   ---------- ---------- ---------- ---------- ---------- ----------
TBRFV Rv3   ---------- ---------- ---------- ---------- ---------- ----------
TBRFV Fw8   ---------- ---------- ---------- ---------- ---------- ----------
TBRFV Rv2   ---------- ---------- ---------- ---------- ---------- ----------
TBRFV Fw9   ---------- ---------- ---------- ---------- ---------- ----------
TBRFV Rv1   ---------- ---------- ---------- ---------- ---------- ----------
```

```
            ....|....| ....|....| ....|....| ....|....| ....|....| ....|....|
                  1990       2000       2010       2020       2030       2040
TBRFV_PT1   AGAGATGTTG AAGAACCATC CATGAAGGGT TCAATGGCAA GAGGAGAGTT ACAATTGGCC
TBRFV_PT2   AGAGATGTTG AAGAACCATC CATGAAGGGT TCAATGGCAA GAGGAGAGTT ACAATTGGCC
TBRFV Fw1   ---------- ---------- ---------- ---------- ---------- ----------
TBRFV Rv9   ---------- ---------- ---------- ---------- ---------- ----------
TBRFV Fw2   ---------- ---------- ---------- ---------- ---------- ----------
TBRFV Rv8   ---------- ---------- ---------- ---------- ---------- ----------
TBRFV Fw3   AGAGATGTTG AAGAACCATC CATGAAGGGT TCAATGGCAA GAGGAGAGTT ACAATTGGCC
TBRFV Rv7   AGAGATGTTG AAGAACCATC CATGAAGGGT TCAATGGCAA GAGGAGAGTT ACAATTGGCC
TBRFV Fw4   ---------- ---------- ---------- ---------- ---------- ----------
TBRFV Rv6   ---------- ---------- ---------- ---------- --GGAGAGTT ACAATTGGCC
TBRFV Fw5   ---------- ---------- ---------- ---------- ---------- ----------
TBRFV Rv5   ---------- ---------- ---------- ---------- ---------- ----------
TBRFV Fw6   ---------- ---------- ---------- ---------- ---------- ----------
TBRFV Rv4   ---------- ---------- ---------- ---------- ---------- ----------
TBRFV Fw7   ---------- ---------- ---------- ---------- ---------- ----------
TBRFV Rv3   ---------- ---------- ---------- ---------- ---------- ----------
TBRFV Fw8   ---------- ---------- ---------- ---------- ---------- ----------
TBRFV Rv2   ---------- ---------- ---------- ---------- ---------- ----------
TBRFV Fw9   ---------- ---------- ---------- ---------- ---------- ----------
TBRFV Rv1   ---------- ---------- ---------- ---------- ---------- ----------
```

```
              ....|....| ....|....| ....|....| ....|....| ....|....| ....|....|
                      2050       2060       2070       2080       2090       2100
TBRFV_PT1     GGTCTGTCTG GAGACCAACC AGAGTCTTCC TATACTCGGA ACGAGGAAAT AGAGTCATTA
TBRFV_PT2     GGTCTGTCTG GAGACCAACC AGAGTCTTCC TATACTCGGA ACGAGGAAAT AGAGTCATTA
TBRFV Fw1     ---------- ---------- ---------- ---------- ---------- ----------
TBRFV Rv9     ---------- ---------- ---------- ---------- ---------- ----------
TBRFV Fw2     ---------- ---------- ---------- ---------- ---------- ----------
TBRFV Rv8     ---------- ---------- ---------- ---------- ---------- ----------
TBRFV Fw3     GGTCTGTCTG GAGACCAACC AGAGTCTTCC TATACTCGGA ACGAGGAAAT AGAGTCATTA
TBRFV Rv7     GGTCTGTCTG GAGACCAACC AGAGTCT-C- ---------- ---------- ----------
TBRFV Fw4     ---------- -----CAACC AGAGTCTTCC TATACTCGGA ACGAGGAAAT AGAGTCATTA
TBRFV Rv6     GGTCTGTCTG GAGACCAACC AGAGTCTTCC TATACTCGGA ACGAGGAAAT AGAGTCATTA
TBRFV Fw5     ---------- ---------- ---------- ---------- ---------- ----------
TBRFV Rv5     ---------- ---------- ---------- ---------- ---------- ----------
TBRFV Fw6     ---------- ---------- ---------- ---------- ---------- ----------
TBRFV Rv4     ---------- ---------- ---------- ---------- ---------- ----------
TBRFV Fw7     ---------- ---------- ---------- ---------- ---------- ----------
TBRFV Rv3     ---------- ---------- ---------- ---------- ---------- ----------
TBRFV Fw8     ---------- ---------- ---------- ---------- ---------- ----------
TBRFV Rv2     ---------- ---------- ---------- ---------- ---------- ----------
TBRFV Fw9     ---------- ---------- ---------- ---------- ---------- ----------
TBRFV Rv1     ---------- ---------- ---------- ---------- ---------- ----------
```

```
              ....|....| ....|....| ....|....| ....|....| ....|....| ....|....|
                      2110       2120       2130       2140       2150       2160
TBRFV_PT1     GAGCAATTCC ACATGGCAAC GGCTAGTTCG TTAATTCGGA AACAGATGAG TTCGATTGTG
TBRFV_PT2     GAGCAATTCC ACATGGCAAC GGCTAGTTCG TTAATTCGGA AACAGATGAG TTCGATTGTG
TBRFV Fw1     ---------- ---------- ---------- ---------- ---------- ----------
TBRFV Rv9     ---------- ---------- ---------- ---------- ---------- ----------
TBRFV Fw2     ---------- ---------- ---------- ---------- ---------- ----------
TBRFV Rv8     ---------- ---------- ---------- ---------- ---------- ----------
TBRFV Fw3     GAGCAAT--- ---------- ---------- ---------- ---------- ----------
TBRFV Rv7     ---------- ---------- ---------- ---------- ---------- ----------
TBRFV Fw4     GAGCAATTCC ACATGGCAAC GGCTAGTTCG TTAATTCGGA AACAGATGAG TTCGATTGTG
TBRFV Rv6     GAGCAATTCC ACATGGCAAC GGCTAGTTCG TTAATTCGGA AACAGATGAG TTCGATTGTG
TBRFV Fw5     ---------- ---------- ---------- ---------- ---------- ----------
TBRFV Rv5     ---------- ---------- ---------- ---------- ---------- ----------
TBRFV Fw6     ---------- ---------- ---------- ---------- ---------- ----------
TBRFV Rv4     ---------- ---------- ---------- ---------- ---------- ----------
TBRFV Fw7     ---------- ---------- ---------- ---------- ---------- ----------
TBRFV Rv3     ---------- ---------- ---------- ---------- ---------- ----------
TBRFV Fw8     ---------- ---------- ---------- ---------- ---------- ----------
TBRFV Rv2     ---------- ---------- ---------- ---------- ---------- ----------
TBRFV Fw9     ---------- ---------- ---------- ---------- ---------- ----------
TBRFV Rv1     ---------- ---------- ---------- ---------- ---------- ----------
```

```
              ....|....| ....|....| ....|....| ....|....| ....|....| ....|....|
                    2170       2180       2190       2200       2210       2220
TBRFV_PT1     TACACGGGCC CCATTAAAGT TCAGCAAATG AAAAACTTTA TTGATAGCCT GGTAGCATCA
TBRFV_PT2     TACACGGGCC CCATTAAAGT TCAGCAAATG AAAAACTTTA TTGATAGCCT GGTAGCATCA
TBRFV Fw1     ---------- ---------- ---------- ---------- ---------- ----------
TBRFV Rv9     ---------- ---------- ---------- ---------- ---------- ----------
TBRFV Fw2     ---------- ---------- ---------- ---------- ---------- ----------
TBRFV Rv8     ---------- ---------- ---------- ---------- ---------- ----------
TBRFV Fw3     ---------- ---------- ---------- ---------- ---------- ----------
TBRFV Rv7     ---------- ---------- ---------- ---------- ---------- ----------
TBRFV Fw4     TACACGGGCC CCATTAAAGT TCAGCAAATG AAAAACTTTA TTGATAGCCT GGTAGCATCA
TBRFV Rv6     TACACGGGCC CCATTAAAGT TCAGCAAATG AAAAACTTTA TTGATAGCCT GGTAGCATCA
TBRFV Fw5     ---------- ---------- ---------- ---------- ---------- ----------
TBRFV Rv5     ---------- ---------- ---------- ---------- ---------- ----------
TBRFV Fw6     ---------- ---------- ---------- ---------- ---------- ----------
TBRFV Rv4     ---------- ---------- ---------- ---------- ---------- ----------
TBRFV Fw7     ---------- ---------- ---------- ---------- ---------- ----------
TBRFV Rv3     ---------- ---------- ---------- ---------- ---------- ----------
TBRFV Fw8     ---------- ---------- ---------- ---------- ---------- ----------
TBRFV Rv2     ---------- ---------- ---------- ---------- ---------- ----------
TBRFV Fw9     ---------- ---------- ---------- ---------- ---------- ----------
TBRFV Rv1     ---------- ---------- ---------- ---------- ---------- ----------

              ....|....| ....|....| ....|....| ....|....| ....|....| ....|....|
                    2230       2240       2250       2260       2270       2280
TBRFV_PT1     CTCTCTGCTG CGGTGTCAAA CCTAGTCAAG ATCCTAAAGG ATACAGCTGC TATAGATCTC
TBRFV_PT2     CTCTCTGCTG CGGTGTCAAA CCTAGTCAAG ATCCTAAAGG ATACAGCTGC TATAGATCTC
TBRFV Fw1     ---------- ---------- ---------- ---------- ---------- ----------
TBRFV Rv9     ---------- ---------- ---------- ---------- ---------- ----------
TBRFV Fw2     ---------- ---------- ---------- ---------- ---------- ----------
TBRFV Rv8     ---------- ---------- ---------- ---------- ---------- ----------
TBRFV Fw3     ---------- ---------- ---------- ---------- ---------- ----------
TBRFV Rv7     ---------- ---------- ---------- ---------- ---------- ----------
TBRFV Fw4     CTCTCTGCTG CGGTGTCAAA CCTAGTCAAG ATCCTAAAGG ATACAGCTGC TATAGATCTC
TBRFV Rv6     CTCTCTGCTG CGGTGTCAAA CCTAGTCAAG ATCCTAAAGG ATACAGCTGC TATAGATCTC
TBRFV Fw5     ---------- ---------- ---------- ---------- ---------- ----------
TBRFV Rv5     ---------- ---------- ---------- ---------- ---------- ----------
TBRFV Fw6     ---------- ---------- ---------- ---------- ---------- ----------
TBRFV Rv4     ---------- ---------- ---------- ---------- ---------- ----------
TBRFV Fw7     ---------- ---------- ---------- ---------- ---------- ----------
TBRFV Rv3     ---------- ---------- ---------- ---------- ---------- ----------
TBRFV Fw8     ---------- ---------- ---------- ---------- ---------- ----------
TBRFV Rv2     ---------- ---------- ---------- ---------- ---------- ----------
TBRFV Fw9     ---------- ---------- ---------- ---------- ---------- ----------
TBRFV Rv1     ---------- ---------- ---------- ---------- ---------- ----------

              ....|....| ....|....| ....|....| ....|....| ....|....| ....|....|
                    2290       2300       2310       2320       2330       2340
TBRFV_PT1     GAGACCCGTC AGAAGTTTGG AGTCTTAGAT GTTGCGACCA AAAGATGGTT AATTAAACCT
TBRFV_PT2     GAGACCCGTC AGAAGTTTGG AGTCTTAGAT GTTGCGACCA AAAGATGGTT AATTAAACCT
TBRFV Fw1     ---------- ---------- ---------- ---------- ---------- ----------
TBRFV Rv9     ---------- ---------- ---------- ---------- ---------- ----------
```

```
TBRFV Fw2    ---------- ---------- ---------- ---------- ---------- ----------
TBRFV Rv8    ---------- ---------- ---------- ---------- ---------- ----------
TBRFV Fw3    ---------- ---------- ---------- ---------- ---------- ----------
TBRFV Rv7    ---------- ---------- ---------- ---------- ---------- ----------
TBRFV Fw4    GAGACCCGTC AGAAGTTTGG AGTCTTAGAT GTTGCGACCA AAAGATGGTT AATTAAACCT
TBRFV Rv6    GAGACCCGTC AGAAGTTTGG AGTCTTAGAT GTTGCGACCA AAAGATGGTT AATTAAACCT
TBRFV Fw5    ---------- ---------- ---------- ---------- ---------- ----------
TBRFV Rv5    ---------- ---------- ---------- ---------- ---------- ----------
TBRFV Fw6    ---------- ---------- ---------- ---------- ---------- ----------
TBRFV Rv4    ---------- ---------- ---------- ---------- ---------- ----------
TBRFV Fw7    ---------- ---------- ---------- ---------- ---------- ----------
TBRFV Rv3    ---------- ---------- ---------- ---------- ---------- ----------
TBRFV Fw8    ---------- ---------- ---------- ---------- ---------- ----------
TBRFV Rv2    ---------- ---------- ---------- ---------- ---------- ----------
TBRFV Fw9    ---------- ---------- ---------- ---------- ---------- ----------
TBRFV Rv1    ---------- ---------- ---------- ---------- ---------- ----------

             ....|....| ....|....| ....|....| ....|....| ....|....| ....|....|
                   2350       2360       2370       2380       2390       2400
TBRFV_PT1    TCAGCCAAGA ATCACGCATG GGGCGTTATT GAAACACATG CTAGGAAGTA CCACGTTGCA
TBRFV_PT2    TCAGCCAAGA ATCACGCATG GGGCGTTATT GAAACACATG CTAGGAAGTA CCACGTTGCA
TBRFV Fw1    ---------- ---------- ---------- ---------- ---------- ----------
TBRFV Rv9    ---------- ---------- ---------- ---------- ---------- ----------
TBRFV Fw2    ---------- ---------- ---------- ---------- ---------- ----------
TBRFV Rv8    ---------- ---------- ---------- ---------- ---------- ----------
TBRFV Fw3    ---------- ---------- ---------- ---------- ---------- ----------
TBRFV Rv7    ---------- ---------- ---------- ---------- ---------- ----------
TBRFV Fw4    TCAGCCAAGA ATCACGCATG GGGCGTTATT GAAACACATG CTAGGAAGTA CCACGTTGCA
TBRFV Rv6    TCAGCCAAGA ATCACGCATG GGGCGTTATT GAAACACATG CTAGGAAGTA CCACGTTGCA
TBRFV Fw5    ---------- ---------- ---------- ---------- ---------- ----------
TBRFV Rv5    ---------- ---------- ---------- ---------- ---------- ----------
TBRFV Fw6    ---------- ---------- ---------- ---------- ---------- ----------
TBRFV Rv4    ---------- ---------- ---------- ---------- ---------- ----------
TBRFV Fw7    ---------- ---------- ---------- ---------- ---------- ----------
TBRFV Rv3    ---------- ---------- ---------- ---------- ---------- ----------
TBRFV Fw8    ---------- ---------- ---------- ---------- ---------- ----------
TBRFV Rv2    ---------- ---------- ---------- ---------- ---------- ----------
TBRFV Fw9    ---------- ---------- ---------- ---------- ---------- ----------
TBRFV Rv1    ---------- ---------- ---------- ---------- ---------- ----------
```

```
                ....|....| ....|....| ....|....| ....|....| ....|....| ....|....|
                      2410       2420       2430       2440       2450       2460
TBRFV_PT1       CTTTTGGAGT ATGATGAGCA TGGAGTGGTA ACTTGCGACA GTTGGAGAAG GGTGGCCGTG
TBRFV_PT2       CTTTTGGAGT ATGATGAGCA TGGAGTGGTA ACTTGCGACA GTTGGAGAAG GGTGGCCGTG
TBRFV Fw1       ---------- ---------- ---------- ---------- ---------- ----------
TBRFV Rv9       ---------- ---------- ---------- ---------- ---------- ----------
TBRFV Fw2       ---------- ---------- ---------- ---------- ---------- ----------
TBRFV Rv8       ---------- ---------- ---------- ---------- ---------- ----------
TBRFV Fw3       ---------- ---------- ---------- ---------- ---------- ----------
TBRFV Rv7       ---------- ---------- ---------- ---------- ---------- ----------
TBRFV Fw4       CTTTTGGAGT ATGATGAGCA TGGAGTGGTA ACTTGCGACA GTTGGAGAAG GGTGGCCGTG
TBRFV Rv6       CTTTTGGAGT ATGATGAGCA TGGAGTGGTA ACTTGCGACA GTTGGAGAAG GGTGGCCGTG
TBRFV Fw5       ---------- ---------- ---------- ---------- ---------- ----------
TBRFV Rv5       ---------- ---------- ---------- ---------- ---------- ----------
TBRFV Fw6       ---------- ---------- ---------- ---------- ---------- ----------
TBRFV Rv4       ---------- ---------- ---------- ---------- ---------- ----------
TBRFV Fw7       ---------- ---------- ---------- ---------- ---------- ----------
TBRFV Rv3       ---------- ---------- ---------- ---------- ---------- ----------
TBRFV Fw8       ---------- ---------- ---------- ---------- ---------- ----------
TBRFV Rv2       ---------- ---------- ---------- ---------- ---------- ----------
TBRFV Fw9       ---------- ---------- ---------- ---------- ---------- ----------
TBRFV Rv1       ---------- ---------- ---------- ---------- ---------- ----------
```

```
                ....|....| ....|....| ....|....| ....|....| ....|....| ....|....|
                      2470       2480       2490       2500       2510       2520
TBRFV_PT1       AGTTCTGAGT CAATGGTTTA TTCTGATATG GCAAAGCTCA GAACACTGAG GAGATTATTA
TBRFV_PT2       AGTTCTGAGT CAATGGTTTA TTCTGATATG GCAAAGCTCA GAACACTGAG GAGATTATTA
TBRFV Fw1       ---------- ---------- ---------- ---------- ---------- ----------
TBRFV Rv9       ---------- ---------- ---------- ---------- ---------- ----------
TBRFV Fw2       ---------- ---------- ---------- ---------- ---------- ----------
TBRFV Rv8       ---------- ---------- ---------- ---------- ---------- ----------
TBRFV Fw3       ---------- ---------- ---------- ---------- ---------- ----------
TBRFV Rv7       ---------- ---------- ---------- ---------- ---------- ----------
TBRFV Fw4       AGTTCTGAGT CAATGGTTTA TTCTGATATG GCAAAGCTCA GAACACTGAG GAGATTATTA
TBRFV Rv6       AGTTCTGAGT CAATGGTTTA TTCTGATATG GCAAAGCTCA GAACACTGAG GAGATTATTA
TBRFV Fw5       ---------- ---------- ---------- ---------- ---------- ----------
TBRFV Rv5       ---------- ---------- ---------- ---------- ---------- ----------
TBRFV Fw6       ---------- ---------- ---------- ---------- ---------- ----------
TBRFV Rv4       ---------- ---------- ---------- ---------- ---------- ----------
TBRFV Fw7       ---------- ---------- ---------- ---------- ---------- ----------
TBRFV Rv3       ---------- ---------- ---------- ---------- ---------- ----------
TBRFV Fw8       ---------- ---------- ---------- ---------- ---------- ----------
TBRFV Rv2       ---------- ---------- ---------- ---------- ---------- ----------
TBRFV Fw9       ---------- ---------- ---------- ---------- ---------- ----------
TBRFV Rv1       ---------- ---------- ---------- ---------- ---------- ----------
```

```
               ....|....| ....|....| ....|....| ....|....| ....|....| ....|....|
                        2530       2540       2550       2560       2570       2580
TBRFV_PT1      AGAGATGGTG AGCCTCATGT CAGCAGTGCT AAAGTCGTCC TAGTTGACGG TGTCCCGGGT
TBRFV_PT2      AGAGATGGTG AGCCTCATGT CAGCAGTGCT AAAGTCGTCC TAGTTGACGG TGTCCCGGGT
TBRFV_Fw1      ---------- ---------- ---------- ---------- ---------- ----------
TBRFV Rv9      ---------- ---------- ---------- ---------- ---------- ----------
TBRFV Fw2      ---------- ---------- ---------- ---------- ---------- ----------
TBRFV Rv8      ---------- ---------- ---------- ---------- ---------- ----------
TBRFV Fw3      ---------- ---------- ---------- ---------- ---------- ----------
TBRFV Rv7      ---------- ---------- ---------- ---------- ---------- ----------
TBRFV Fw4      AGAGATGGTG AGCCTCATGT CAGCAGTGCT AAAGTCGTCC TAGTTGACGG TGTCCCGGGT
TBRFV Rv6      AGAGATGGTG AGCCTCATGT CAGCAGTGCT AAAGTCGTCC TAGTTGACGG TGTCCCGGGT
TBRFV Fw5      ---------- ---------- ---------- ---------- ---------- ----------
TBRFV Rv5      ---------- ---------- ---------- ---------- ---------- ----------
TBRFV Fw6      ---------- ---------- ---------- ---------- ---------- ----------
TBRFV Rv4      ---------- ---------- ---------- ---------- ---------- ----------
TBRFV Fw7      ---------- ---------- ---------- ---------- ---------- ----------
TBRFV Rv3      ---------- ---------- ---------- ---------- ---------- ----------
TBRFV Fw8      ---------- ---------- ---------- ---------- ---------- ----------
TBRFV Rv2      ---------- ---------- ---------- ---------- ---------- ----------
TBRFV Fw9      ---------- ---------- ---------- ---------- ---------- ----------
TBRFV Rv1      ---------- ---------- ---------- ---------- ---------- ----------

               ....|....| ....|....| ....|....| ....|....| ....|....| ....|....|
                        2590       2600       2610       2620       2630       2640
TBRFV_PT1      TGTGGAAAGA CAAAAGAGAT TCTCTCGAAA GTAAATTTTG AGGAAGATCT AATCTTAGTA
TBRFV_PT2      TGTGGAAAGA CAAAAGAGAT TCTCTCGAAA GTAAATTTTG AGGAAGATCT AATCTTAGTA
TBRFV_Fw1      ---------- ---------- ---------- ---------- ---------- ----------
TBRFV Rv9      ---------- ---------- ---------- ---------- ---------- ----------
TBRFV Fw2      ---------- ---------- ---------- ---------- ---------- ----------
TBRFV Rv8      ---------- ---------- ---------- ---------- ---------- ----------
TBRFV Fw3      ---------- ---------- ---------- ---------- ---------- ----------
TBRFV Rv7      ---------- ---------- ---------- ---------- ---------- ----------
TBRFV Fw4      TGTGGAAAGA CAAAAGAGAT TCTCTCGAAA GTAAATTTTG AGGAAGATCT AATCTTAGTA
TBRFV Rv6      TGTGGAAAGA CAAAAGAGAT TCTCTCGAAA GTAAATTTTG AGGAAGATCT AATCTTAGTA
TBRFV Fw5      ---------- ---------- ---------- ---------- ---------- ----------
TBRFV Rv5      ---------- ---------- ---------- ---------- ---------- ----------
TBRFV Fw6      ---------- ---------- ---------- ---------- ---------- ----------
TBRFV Rv4      ---------- ---------- ---------- ---------- ---------- ----------
TBRFV Fw7      ---------- ---------- ---------- ---------- ---------- ----------
TBRFV Rv3      ---------- ---------- ---------- ---------- ---------- ----------
TBRFV Fw8      ---------- ---------- ---------- ---------- ---------- ----------
TBRFV Rv2      ---------- ---------- ---------- ---------- ---------- ----------
TBRFV Fw9      ---------- ---------- ---------- ---------- ---------- ----------
TBRFV Rv1      ---------- ---------- ---------- ---------- ---------- ----------

               ....|....| ....|....| ....|....| ....|....| ....|....| ....|....|
                        2650       2660       2670       2680       2690       2700
TBRFV_PT1      CCGGGTAAGC AGGCTGCTGA AATGATAAAG AGGCGTGCTA ATGCGTCAGG AATAATTCAA
TBRFV_PT2      CCGGGTAAGC AGGCTGCTGA AATGATAAAG AGGCGTGCTA ATGCGTCAGG AATAATTCAA
```

```
TBRFV Fw1     ---------- ---------- ---------- ---------- ---------- ----------
TBRFV Rv9     ---------- ---------- ---------- ---------- ---------- ----------
TBRFV Fw2     ---------- ---------- ---------- ---------- ---------- ----------
TBRFV Rv8     ---------- ---------- ---------- ---------- ---------- ----------
TBRFV Fw3     ---------- ---------- ---------- ---------- ---------- ----------
TBRFV Rv7     ---------- ---------- ---------- ---------- ---------- ----------
TBRFV Fw4     CCGGGTAAGC AGGCTGCTGA AATGATAAAG AGGCGTGCTA ATGCGTCAGG AATAATTCAA
TBRFV Rv6     CCGGGTAAGC AGGCTGCTGA AATGATAAAG AGGCGTGCTA ATGCGTCAGG AATAATTCAA
TBRFV Fw5     ---------- ---------- ---------- ---------- ---------- ----------
TBRFV Rv5     ---------- ---------- ---------- ---------- ---------- ----------
TBRFV Fw6     ---------- ---------- ---------- ---------- ---------- ----------
TBRFV Rv4     ---------- ---------- ---------- ---------- ---------- ----------
TBRFV Fw7     ---------- ---------- ---------- ---------- ---------- ----------
TBRFV Rv3     ---------- ---------- ---------- ---------- ---------- ----------
TBRFV Fw8     ---------- ---------- ---------- ---------- ---------- ----------
TBRFV Rv2     ---------- ---------- ---------- ---------- ---------- ----------
TBRFV Fw9     ---------- ---------- ---------- ---------- ---------- ----------
TBRFV Rv1     ---------- ---------- ---------- ---------- ---------- ----------

              ....|....| ....|....| ....|....| ....|....| ....|....| ....|....|
                    2710       2720       2730       2740       2750       2760
TBRFV_PT1     GCCACAAGAG ATAATGTTCG TACTGTTGAT TCATTTATAA TGAATTACGG TAAAGGAACA
TBRFV_PT2     GCCACAAGAG ATAATGTTCG TACTGTTGAT TCATTTATAA TGAATTACGG TAAAGGAACA
TBRFV Fw1     ---------- ---------- ---------- ---------- ---------- ----------
TBRFV Rv9     ---------- ---------- ---------- ---------- ---------- ----------
TBRFV Fw2     ---------- ---------- ---------- ---------- ---------- ----------
TBRFV Rv8     ---------- ---------- ---------- ---------- ---------- ----------
TBRFV Fw3     ---------- ---------- ---------- ---------- ---------- ----------
TBRFV Rv7     ---------- ---------- ---------- ---------- ---------- ----------
TBRFV Fw4     GCCACAAGAG ATAATGTTCG TACTGTTGAT TCATTTATAA TGAATTACGG TAAAGGAACA
TBRFV Rv6     GCCACAAGAG ATAATGTTCG TACTGTTGAT TCATTTATAA TGAATTACGG TAAAGGAACA
TBRFV Fw5     ---------- ---------- ---------- ---------- ------ACGG TAAAGGAACA
TBRFV Rv5     ---------- ---ATGTTCG TACTGTTGAT TCATTTATAA TGAATTACGG TAAAGGAACA
TBRFV Fw6     ---------- ---------- ---------- ---------- ---------- ----------
TBRFV Rv4     ---------- ---------- ---------- ---------- ---------- ----------
TBRFV Fw7     ---------- ---------- ---------- ---------- ---------- ----------
TBRFV Rv3     ---------- ---------- ---------- ---------- ---------- ----------
TBRFV Fw8     ---------- ---------- ---------- ---------- ---------- ----------
TBRFV Rv2     ---------- ---------- ---------- ---------- ---------- ----------
TBRFV Fw9     ---------- ---------- ---------- ---------- ---------- ----------
TBRFV Rv1     ---------- ---------- ---------- ---------- ---------- ----------
```

https://doi.org/10.3390/plants15081240

```
           ....|....| ....|....| ....|....| ....|....| ....|....| ....|....|
                 2770       2780       2790       2800       2810       2820
TBRFV_PT1  CGCTGTCAGT TCAAAAGGTT ATTTATCGAC GAAGGTCTGA TGTTGCACAC TGGTTGTGTG
TBRFV_PT2  CGCTGTCAGT TCAAAAGGTT ATTTATCGAC GAAGGTCTGA TGTTGCACAC TGGTTGTGTG
TBRFV Fw1  ---------- ---------- ---------- ---------- ---------- ----------
TBRFV Rv9  ---------- ---------- ---------- ---------- ---------- ----------
TBRFV Fw2  ---------- ---------- ---------- ---------- ---------- ----------
TBRFV Rv8  ---------- ---------- ---------- ---------- ---------- ----------
TBRFV Fw3  ---------- ---------- ---------- ---------- ---------- ----------
TBRFV Rv7  ---------- ---------- ---------- ---------- ---------- ----------
TBRFV Fw4  CGCTGTCAGT TCAAAAGGTT ATTTATCGAC GAAGGTCTGA TGT------- ----------
TBRFV Rv6  CGCTGTCAGT TCAAAA---- ---------- ---------- ---------- ----------
TBRFV Fw5  CGCTGTCAGT TCAAAAGGTT ATTTATCGAC GAAGGTCTGA TGTTGCACAC TGGTTGTGTG
TBRFV Rv5  CGCTGTCAGT TCAAAAGGTT ATTTATCGAC GAAGGTCTGA TGTTGCACAC TGGTTGTGTG
TBRFV Fw6  ---------- ---------- ---------- ---------- ---------- ----------
TBRFV Rv4  ---------- ---------- ---------- ---------- ---------- ----------
TBRFV Fw7  ---------- ---------- ---------- ---------- ---------- ----------
TBRFV Rv3  ---------- ---------- ---------- ---------- ---------- ----------
TBRFV Fw8  ---------- ---------- ---------- ---------- ---------- ----------
TBRFV Rv2  ---------- ---------- ---------- ---------- ---------- ----------
TBRFV Fw9  ---------- ---------- ---------- ---------- ---------- ----------
TBRFV Rv1  ---------- ---------- ---------- ---------- ---------- ----------
```

```
           ....|....| ....|....| ....|....| ....|....| ....|....| ....|....|
                 2830       2840       2850       2860       2870       2880
TBRFV_PT1  AATTTCCTTG TTTCTATGTC TCTGTGCGAA ATTGCATATG TTTATGGAGA CACACAACAA
TBRFV_PT2  AATTTTCTTG TTTCTATGTC TCTGTGCGAA ATTGCATATG TTTATGGAGA CACACAACAA
TBRFV Fw1  ---------- ---------- ---------- ---------- ---------- ----------
TBRFV Rv9  ---------- ---------- ---------- ---------- ---------- ----------
TBRFV Fw2  ---------- ---------- ---------- ---------- ---------- ----------
TBRFV Rv8  ---------- ---------- ---------- ---------- ---------- ----------
TBRFV Fw3  ---------- ---------- ---------- ---------- ---------- ----------
TBRFV Rv7  ---------- ---------- ---------- ---------- ---------- ----------
TBRFV Fw4  ---------- ---------- ---------- ---------- ---------- ----------
TBRFV Rv6  ---------- ---------- ---------- ---------- ---------- ----------
TBRFV Fw5  AATTTTCTTG TTTCTATGTC TCTGTGCGAA ATTGCATATG TTTATGGAGA CACACAACAA
TBRFV Rv5  AATTTTCTTG TTTCTATGTC TCTGTGCGAA ATTGCATATG TTTATGGAGA CACACAACAA
TBRFV Fw6  ---------- ---------- ---------- ---------- ---------- ----------
TBRFV Rv4  ---------- ---------- ---------- ---------- ---------- ----------
TBRFV Fw7  ---------- ---------- ---------- ---------- ---------- ----------
TBRFV Rv3  ---------- ---------- ---------- ---------- ---------- ----------
TBRFV Fw8  ---------- ---------- ---------- ---------- ---------- ----------
TBRFV Rv2  ---------- ---------- ---------- ---------- ---------- ----------
TBRFV Fw9  ---------- ---------- ---------- ---------- ---------- ----------
TBRFV Rv1  ---------- ---------- ---------- ---------- ---------- ----------
```

```
               ....|....| ....|....| ....|....| ....|....| ....|....| ....|....|
                      2890       2900       2910       2920       2930       2940
TBRFV_PT1      ATTCCATACA TCAACAGAGT ATCCGGTTTT CCGTACCCTG CACATTTTGC AAAAATAGAG
TBRFV_PT2      ATTCCATACA TCAACAGAGT ATCCGGTTTT CCGTACCCTG CACATTTTGC AAAAATAGAG
TBRFV Fw1      ---------- ---------- ---------- ---------- ---------- ----------
TBRFV Rv9      ---------- ---------- ---------- ---------- ---------- ----------
TBRFV Fw2      ---------- ---------- ---------- ---------- ---------- ----------
TBRFV Rv8      ---------- ---------- ---------- ---------- ---------- ----------
TBRFV Fw3      ---------- ---------- ---------- ---------- ---------- ----------
TBRFV Rv7      ---------- ---------- ---------- ---------- ---------- ----------
TBRFV Fw4      ---------- ---------- ---------- ---------- ---------- ----------
TBRFV Rv6      ---------- ---------- ---------- ---------- ---------- ----------
TBRFV Fw5      ATTCCATACA TCAACAGAGT ATCCGGTTTT CCGTACCCTG CACATTTTGC AAAAATAGAG
TBRFV Rv5      ATTCCATACA TCAACAGAGT ATCCGGTTTT CCGTACCCTG CACATTTTGC AAAAATAGAG
TBRFV Fw6      ---------- ---------- ---------- ---------- ---------- ----------
TBRFV Rv4      ---------- ---------- ---------- ---------- ---------- ----------
TBRFV Fw7      ---------- ---------- ---------- ---------- ---------- ----------
TBRFV Rv3      ---------- ---------- ---------- ---------- ---------- ----------
TBRFV Fw8      ---------- ---------- ---------- ---------- ---------- ----------
TBRFV Rv2      ---------- ---------- ---------- ---------- ---------- ----------
TBRFV Fw9      ---------- ---------- ---------- ---------- ---------- ----------
TBRFV Rv1      ---------- ---------- ---------- ---------- ---------- ----------
```

```
               ....|....| ....|....| ....|....| ....|....| ....|....| ....|....|
                      2950       2960       2970       2980       2990       3000
TBRFV_PT1      GTTGATGAGG TAGAAACTCG CAGAACTACG CTGCGTTGTC CAGCCGACAT TACCCACTAT
TBRFV_PT2      GTTGATGAGG TAGAAACTCG CAGAACTACG CTGCGTTGTC CAGCCGACAT TACCCACTAT
TBRFV Fw1      ---------- ---------- ---------- ---------- ---------- ----------
TBRFV Rv9      ---------- ---------- ---------- ---------- ---------- ----------
TBRFV Fw2      ---------- ---------- ---------- ---------- ---------- ----------
TBRFV Rv8      ---------- ---------- ---------- ---------- ---------- ----------
TBRFV Fw3      ---------- ---------- ---------- ---------- ---------- ----------
TBRFV Rv7      ---------- ---------- ---------- ---------- ---------- ----------
TBRFV Fw4      ---------- ---------- ---------- ---------- ---------- ----------
TBRFV Rv6      ---------- ---------- ---------- ---------- ---------- ----------
TBRFV Fw5      GTTGATGAGG TAGAAACTCG CAGAACTACG CTGCGTTGTC CAGCCGACAT TACCCACTAT
TBRFV Rv5      GTTGATGAGG TAGAAACTCG CAGAACTACG CTGCGTTGTC CAGCCGACAT TACCCACTAT
TBRFV Fw6      ---------- ---------- ---------- ---------- ---------- ----------
TBRFV Rv4      ---------- ---------- ---------- ---------- ---------- ----------
TBRFV Fw7      ---------- ---------- ---------- ---------- ---------- ----------
TBRFV Rv3      ---------- ---------- ---------- ---------- ---------- ----------
TBRFV Fw8      ---------- ---------- ---------- ---------- ---------- ----------
TBRFV Rv2      ---------- ---------- ---------- ---------- ---------- ----------
TBRFV Fw9      ---------- ---------- ---------- ---------- ---------- ----------
TBRFV Rv1      ---------- ---------- ---------- ---------- ---------- ----------
```

```
               ....|....| ....|....| ....|....| ....|....| ....|....| ....|....|
                      3010       3020       3030       3040       3050       3060
TBRFV_PT1      CTTAACAAAA GGTACGAAGG ATATGTCATG TGTACATCGT CGGTTAAAAA GTCAGTTTCT
TBRFV_PT2      CTTAACAAAA GGTACGAAGG ATATGTCATG TGTACATCGT CGGTTAAAAA GTCAGTTTCT
TBRFV Fw1      ---------- ---------- ---------- ---------- ---------- ----------
TBRFV Rv9      ---------- ---------- ---------- ---------- ---------- ----------
TBRFV Fw2      ---------- ---------- ---------- ---------- ---------- ----------
TBRFV Rv8      ---------- ---------- ---------- ---------- ---------- ----------
TBRFV Fw3      ---------- ---------- ---------- ---------- ---------- ----------
TBRFV Rv7      ---------- ---------- ---------- ---------- ---------- ----------
TBRFV Fw4      ---------- ---------- ---------- ---------- ---------- ----------
TBRFV Rv6      ---------- ---------- ---------- ---------- ---------- ----------
TBRFV Fw5      CTTAACAAAA GGTACGAAGG ATATGTCATG TGTACATCGT CGGTTAAAAA GTCAGTTTCT
TBRFV Rv5      CTTAACAAAA GGTACGAAGG ATATGTCATG TGTACATCGT CGGTTAAAAA GTCAGTTTCT
TBRFV Fw6      ---------- ---------- ---------- ---------- ---------- ----------
TBRFV Rv4      ---------- ---------- ---------- ---------- ---------- ----------
TBRFV Fw7      ---------- ---------- ---------- ---------- ---------- ----------
TBRFV Rv3      ---------- ---------- ---------- ---------- ---------- ----------
TBRFV Fw8      ---------- ---------- ---------- ---------- ---------- ----------
TBRFV Rv2      ---------- ---------- ---------- ---------- ---------- ----------
TBRFV Fw9      ---------- ---------- ---------- ---------- ---------- ----------
TBRFV Rv1      ---------- ---------- ---------- ---------- ---------- ----------

               ....|....| ....|....| ....|....| ....|....| ....|....| ....|....|
                      3070       3080       3090       3100       3110       3120
TBRFV_PT1      CAGGAAATGG TGAGCGGGGC CGCAATGATC AATCCTGTAT CTAAGCCATT GAATGGGAAA
TBRFV_PT2      CAGGAAATGG TGAGCGGGGC CGCAATGATC AATCCTGTAT CTAAGCCATT GAATGGGAAA
TBRFV Fw1      ---------- ---------- ---------- ---------- ---------- ----------
TBRFV Rv9      ---------- ---------- ---------- ---------- ---------- ----------
TBRFV Fw2      ---------- ---------- ---------- ---------- ---------- ----------
TBRFV Rv8      ---------- ---------- ---------- ---------- ---------- ----------
TBRFV Fw3      ---------- ---------- ---------- ---------- ---------- ----------
TBRFV Rv7      ---------- ---------- ---------- ---------- ---------- ----------
TBRFV Fw4      ---------- ---------- ---------- ---------- ---------- ----------
TBRFV Rv6      ---------- ---------- ---------- ---------- ---------- ----------
TBRFV Fw5      CAGGAAATGG TGAGCGGGGC CGCAATGATC AATCCTGTAT CTAAGCCATT GAATGGGAAA
TBRFV Rv5      CAGGAAATGG TGAGCGGGGC CGCAATGATC AATCCTGTAT CTAAGCCATT GAATGGGAAA
TBRFV Fw6      ---------- ---------- ---------- ---------- ---------- ----------
TBRFV Rv4      ---------- ---------- ---------- ---------- ---------- ----------
TBRFV Fw7      ---------- ---------- ---------- ---------- ---------- ----------
TBRFV Rv3      ---------- ---------- ---------- ---------- ---------- ----------
TBRFV Fw8      ---------- ---------- ---------- ---------- ---------- ----------
TBRFV Rv2      ---------- ---------- ---------- ---------- ---------- ----------
TBRFV Fw9      ---------- ---------- ---------- ---------- ---------- ----------
TBRFV Rv1      ---------- ---------- ---------- ---------- ---------- ----------
```

```
              ....|....| ....|....| ....|....| ....|....| ....|....| ....|....|
                    3130       3140       3150       3160       3170       3180
TBRFV_PT1     GTTTTGACTT TCACTCAGTC TGATAAAGAG GCGCTGCTTT CTCGAGGATA TACGGACGTC
TBRFV_PT2     GTTTTGACTT TCACTCAGTC TGATAAAGAG GCGCTGCTTT CTCGAGGATA TACGGACGTC
TBRFV Fw1     ---------- ---------- ---------- ---------- ---------- ----------
TBRFV Rv9     ---------- ---------- ---------- ---------- ---------- ----------
TBRFV Fw2     ---------- ---------- ---------- ---------- ---------- ----------
TBRFV Rv8     ---------- ---------- ---------- ---------- ---------- ----------
TBRFV Fw3     ---------- ---------- ---------- ---------- ---------- ----------
TBRFV Rv7     ---------- ---------- ---------- ---------- ---------- ----------
TBRFV Fw4     ---------- ---------- ---------- ---------- ---------- ----------
TBRFV Rv6     ---------- ---------- ---------- ---------- ---------- ----------
TBRFV Fw5     GTTTTGACTT TCACTCAGTC TGATAAAGAG GCGCTGCTTT CTCGAGGATA TACGGACGTC
TBRFV Rv5     GTTTTGACTT TCACTCAGTC TGATAAAGAG GCGCTGCTTT CTCGAGGATA TACGGACGTC
TBRFV Fw6     ---------- ---------- ---------- ---------- ---------- ----------
TBRFV Rv4     ---------- ---------- ---------- ---------- ---------- ----------
TBRFV Fw7     ---------- ---------- ---------- ---------- ---------- ----------
TBRFV Rv3     ---------- ---------- ---------- ---------- ---------- ----------
TBRFV Fw8     ---------- ---------- ---------- ---------- ---------- ----------
TBRFV Rv2     ---------- ---------- ---------- ---------- ---------- ----------
TBRFV Fw9     ---------- ---------- ---------- ---------- ---------- ----------
TBRFV Rv1     ---------- ---------- ---------- ---------- ---------- ----------
```

```
              ....|....| ....|....| ....|....| ....|....| ....|....| ....|....|
                    3190       3200       3210       3220       3230       3240
TBRFV_PT1     CATACAGTAC ATGAGGTACA AGGTGAGACA TATGCAGATG TGTCGTTGGT CAGATTGACT
TBRFV_PT2     CATACAGTAC ATGAGGTACA AGGTGAGACA TATGCAGATG TGTCGTTGGT CAGATTGACT
TBRFV Fw1     ---------- ---------- ---------- ---------- ---------- ----------
TBRFV Rv9     ---------- ---------- ---------- ---------- ---------- ----------
TBRFV Fw2     ---------- ---------- ---------- ---------- ---------- ----------
TBRFV Rv8     ---------- ---------- ---------- ---------- ---------- ----------
TBRFV Fw3     ---------- ---------- ---------- ---------- ---------- ----------
TBRFV Rv7     ---------- ---------- ---------- ---------- ---------- ----------
TBRFV Fw4     ---------- ---------- ---------- ---------- ---------- ----------
TBRFV Rv6     ---------- ---------- ---------- ---------- ---------- ----------
TBRFV Fw5     CATACAGTAC ATGAGGTACA AGGTGAGACA TATGCAGATG TGTCGTTGGT CAGATTGACT
TBRFV Rv5     CATACAGTAC ATGAGGTACA AGGTGAGACA TATGCAGATG TGTCGTTGGT CAGATTGACT
TBRFV Fw6     ---------- ---------- ---------- ---------- ---------- ----------
TBRFV Rv4     ---------- ---------- ---------- ---------- ---------- ----------
TBRFV Fw7     ---------- ---------- ---------- ---------- ---------- ----------
TBRFV Rv3     ---------- ---------- ---------- ---------- ---------- ----------
TBRFV Fw8     ---------- ---------- ---------- ---------- ---------- ----------
TBRFV Rv2     ---------- ---------- ---------- ---------- ---------- ----------
TBRFV Fw9     ---------- ---------- ---------- ---------- ---------- ----------
TBRFV Rv1     ---------- ---------- ---------- ---------- ---------- ----------
```

```
              ....|....| ....|....| ....|....| ....|....| ....|....| ....|....|
                    3250       3260       3270       3280       3290       3300
TBRFV_PT1     CCGACACCTG TATCTATCAT CGCAGGAGAT AGTCCGCACG TTCTCGTAGC TTTGTCAAGG
TBRFV_PT2     CCGACACCTG TATCTATCAT CGCAGGAGAT AGTCCGCACG TTCTCGTAGC TTTGTCAAGG
TBRFV Fw1     ---------- ---------- ---------- ---------- ---------- ----------
TBRFV Rv9     ---------- ---------- ---------- ---------- ---------- ----------
TBRFV Fw2     ---------- ---------- ---------- ---------- ---------- ----------
TBRFV Rv8     ---------- ---------- ---------- ---------- ---------- ----------
TBRFV Fw3     ---------- ---------- ---------- ---------- ---------- ----------
TBRFV Rv7     ---------- ---------- ---------- ---------- ---------- ----------
TBRFV Fw4     ---------- ---------- ---------- ---------- ---------- ----------
TBRFV Rv6     ---------- ---------- ---------- ---------- ---------- ----------
TBRFV Fw5     CCGACACCTG TATCTATCAT CGCAGGAGAT AGTCCGCACG TTCTCGTAGC TTTGTCAAGG
TBRFV Rv5     CCGACACCTG TATCTATCAT CGCAGGAGAT AGTCCGCACG TTCTCGTAGC TTTGTCAAGG
TBRFV Fw6     ---------- ---------- ---------- ---------- ---------- ----------
TBRFV Rv4     ---------- ---------- ---------- ---------- ---------- ----------
TBRFV Fw7     ---------- ---------- ---------- ---------- ---------- ----------
TBRFV Rv3     ---------- ---------- ---------- ---------- ---------- ----------
TBRFV Fw8     ---------- ---------- ---------- ---------- ---------- ----------
TBRFV Rv2     ---------- ---------- ---------- ---------- ---------- ----------
TBRFV Fw9     ---------- ---------- ---------- ---------- ---------- ----------
TBRFV Rv1     ---------- ---------- ---------- ---------- ---------- ----------
```

```
              ....|....| ....|....| ....|....| ....|....| ....|....| ....|....|
                    3310       3320       3330       3340       3350       3360
TBRFV_PT1     CATACCCAAA CATTGAAGTA TTACACCGTA GTGATGGATC CTCTTGTAAG TATAATTAGG
TBRFV_PT2     CATACCCAAA CATTGAAGTA TTACACCGTA GTGATGGATC CTCTTGTAAG TATAATTAGG
TBRFV Fw1     ---------- ---------- ---------- ---------- ---------- ----------
TBRFV Rv9     ---------- ---------- ---------- ---------- ---------- ----------
TBRFV Fw2     ---------- ---------- ---------- ---------- ---------- ----------
TBRFV Rv8     ---------- ---------- ---------- ---------- ---------- ----------
TBRFV Fw3     ---------- ---------- ---------- ---------- ---------- ----------
TBRFV Rv7     ---------- ---------- ---------- ---------- ---------- ----------
TBRFV Fw4     ---------- ---------- ---------- ---------- ---------- ----------
TBRFV Rv6     ---------- ---------- ---------- ---------- ---------- ----------
TBRFV Fw5     CATACCCAAA CATTGAAGTA TTACACCGTA GTGATGGATC CTCTTGTAAG TATAATTAGG
TBRFV Rv5     CATACCCAAA CATTGAAGTA TTACACCGTA GTGATGGATC CTCTTGTAAG TATAATTAGG
TBRFV Fw6     ---------- ---------- ---------- ---------- ---------- ----------
TBRFV Rv4     ---------- ---------- ---------- ---------- ---------- ----------
TBRFV Fw7     ---------- ---------- ---------- ---------- ---------- ----------
TBRFV Rv3     ---------- ---------- ---------- ---------- ---------- ----------
TBRFV Fw8     ---------- ---------- ---------- ---------- ---------- ----------
TBRFV Rv2     ---------- ---------- ---------- ---------- ---------- ----------
TBRFV Fw9     ---------- ---------- ---------- ---------- ---------- ----------
TBRFV Rv1     ---------- ---------- ---------- ---------- ---------- ----------
```

```
                ....|....| ....|....| ....|....| ....|....| ....|....| ....|....|
                      3370       3380       3390       3400       3410       3420
TBRFV_PT1       GATTTAGAAA AACTTAGTTC TTACTTGTTA GATATGTATA AAGTAGATGC AGGGACCCAA
TBRFV_PT2       GATTTAGAAA AACTTAGTTC TTACTTGTTA GATATGTATA AAGTAGATGC AGGGACCCAA
TBRFV Fw1       ---------- ---------- ---------- ---------- ---------- ----------
TBRFV Rv9       ---------- ---------- ---------- ---------- ---------- ----------
TBRFV Fw2       ---------- ---------- ---------- ---------- ---------- ----------
TBRFV Rv8       ---------- ---------- ---------- ---------- ---------- ----------
TBRFV Fw3       ---------- ---------- ---------- ---------- ---------- ----------
TBRFV Rv7       ---------- ---------- ---------- ---------- ---------- ----------
TBRFV Fw4       ---------- ---------- ---------- ---------- ---------- ----------
TBRFV Rv6       ---------- ---------- ---------- ---------- ---------- ----------
TBRFV Fw5       GATTTAGAAA AACTTAGTTC TTACTTGTTA GATATGTATA AAGTAGATGC AGGGACCCAA
TBRFV Rv5       GATTTAGAAA AACTTAGTTC TTACTTGTTA GATATGTATA AAGTAGATGC AGGGACCCAA
TBRFV Fw6       ---------- ---------- ---------- ---------- ---------- ----------
TBRFV Rv4       ---------- ---------- ---------- ---------- ---------- ---GACCCAA
TBRFV Fw7       ---------- ---------- ---------- ---------- ---------- ----------
TBRFV Rv3       ---------- ---------- ---------- ---------- ---------- ----------
TBRFV Fw8       ---------- ---------- ---------- ---------- ---------- ----------
TBRFV Rv2       ---------- ---------- ---------- ---------- ---------- ----------
TBRFV Fw9       ---------- ---------- ---------- ---------- ---------- ----------
TBRFV Rv1       ---------- ---------- ---------- ---------- ---------- ----------
```

```
                ....|....| ....|....| ....|....| ....|....| ....|....| ....|....|
                      3430       3440       3450       3460       3470       3480
TBRFV_PT1       TAGCAATTAC AGGTAGACTC CGTGTTTAAA GGTTCTAATC TTTTTGTTGC AGCACCAAAG
TBRFV_PT2       TAGCAATTAC AGGTAGACTC CGTGTTTAAA GGTTCTAATC TTTTTGTTGC AGCACCAAAG
TBRFV Fw1       ---------- ---------- ---------- ---------- ---------- ----------
TBRFV Rv9       ---------- ---------- ---------- ---------- ---------- ----------
TBRFV Fw2       ---------- ---------- ---------- ---------- ---------- ----------
TBRFV Rv8       ---------- ---------- ---------- ---------- ---------- ----------
TBRFV Fw3       ---------- ---------- ---------- ---------- ---------- ----------
TBRFV Rv7       ---------- ---------- ---------- ---------- ---------- ----------
TBRFV Fw4       ---------- ---------- ---------- ---------- ---------- ----------
TBRFV Rv6       ---------- ---------- ---------- ---------- ---------- ----------
TBRFV Fw5       TAGCAATTAC AGGTAGACTC CGTGTTTAAA GGTTCTAATC TTTTTGTTGC AGCACCAAAG
TBRFV Rv5       TAGCAATTAC AGGTAGACTC CGTGTTTAAA GGTTCTAATC TTTTTGTTGC AGCACC----
TBRFV Fw6       ---------- ---------- --TGTTTAA- GGTTCTAATC TTTTTGTTGC AGCACCAAAG
TBRFV Rv4       TAGCAATTAC AGGTAGACTC CGTGTTTAAA GGTTCTAATC TTTTTGTTGC AGCACCAAAG
TBRFV Fw7       ---------- ---------- ---------- ---------- ---------- ----------
TBRFV Rv3       ---------- ---------- ---------- ---------- ---------- ----------
TBRFV Fw8       ---------- ---------- ---------- ---------- ---------- ----------
TBRFV Rv2       ---------- ---------- ---------- ---------- ---------- ----------
TBRFV Fw9       ---------- ---------- ---------- ---------- ---------- ----------
TBRFV Rv1       ---------- ---------- ---------- ---------- ---------- ----------
```

```
                ....|....| ....|....| ....|....| ....|....| ....|....| ....|....|
                      3490       3500       3510       3520       3530       3540
TBRFV_PT1       ACTGGAGATA TCTCAGATAT GCAATTTTAC TATGATAAGT GTCTCCCAGG TAATAGCACC
TBRFV_PT2       ACTGGAGATA TCTCAGATAT GCAATTTTAC TATGATAAGT GTCTCCCAGG TAATAGCACC
TBRFV Fw1       ---------- ---------- ---------- ---------- ---------- ----------
TBRFV Rv9       ---------- ---------- ---------- ---------- ---------- ----------
TBRFV Fw2       ---------- ---------- ---------- ---------- ---------- ----------
TBRFV Rv8       ---------- ---------- ---------- ---------- ---------- ----------
TBRFV Fw3       ---------- ---------- ---------- ---------- ---------- ----------
TBRFV Rv7       ---------- ---------- ---------- ---------- ---------- ----------
TBRFV Fw4       ---------- ---------- ---------- ---------- ---------- ----------
TBRFV Rv6       ---------- ---------- ---------- ---------- ---------- ----------
TBRFV Fw5       ACTGGAGATA TCTCAGATAT GCAATTT--- ---------- ---------- ----------
TBRFV Rv5       ---------- ---------- ---------- ---------- ---------- ----------
TBRFV Fw6       ACTGGAGATA TCTCAGATAT GCAATTTTAC TATGATAAGT GTCTCCCAGG TAATAGCACC
TBRFV Rv4       ACTGGAGATA TCTCAGATAT GCAATTTTAC TATGATAAGT GTCTCCCAGG TAATAGCACC
TBRFV Fw7       ---------- ---------- ---------- ---------- ---------- ----------
TBRFV Rv3       ---------- ---------- ---------- ---------- ---------- ----------
TBRFV Fw8       ---------- ---------- ---------- ---------- ---------- ----------
TBRFV Rv2       ---------- ---------- ---------- ---------- ---------- ----------
TBRFV Fw9       ---------- ---------- ---------- ---------- ---------- ----------
TBRFV Rv1       ---------- ---------- ---------- ---------- ---------- ----------
```

```
                ....|....| ....|....| ....|....| ....|....| ....|....| ....|....|
                      3550       3560       3570       3580       3590       3600
TBRFV_PT1       ATGTTAAATA ACTATGATGC TGTTACCATG AGGTTGACTG ACATTTCTCT TAATGTCAAA
TBRFV_PT2       ATGTTAAATA ACTATGATGC TGTTACCATG AGGTTGACTG ACATTTCTCT TAATGTCAAA
TBRFV Fw1       ---------- ---------- ---------- ---------- ---------- ----------
TBRFV Rv9       ---------- ---------- ---------- ---------- ---------- ----------
TBRFV Fw2       ---------- ---------- ---------- ---------- ---------- ----------
TBRFV Rv8       ---------- ---------- ---------- ---------- ---------- ----------
TBRFV Fw3       ---------- ---------- ---------- ---------- ---------- ----------
TBRFV Rv7       ---------- ---------- ---------- ---------- ---------- ----------
TBRFV Fw4       ---------- ---------- ---------- ---------- ---------- ----------
TBRFV Rv6       ---------- ---------- ---------- ---------- ---------- ----------
TBRFV Fw5       ---------- ---------- ---------- ---------- ---------- ----------
TBRFV Rv5       ---------- ---------- ---------- ---------- ---------- ----------
TBRFV Fw6       ATGTTAAATA ACTATGATGC TGTTACCATG AGGTTGACTG ACATTTCTCT TAATGTCAAA
TBRFV Rv4       ATGTTAAATA ACTATGATGC TGTTACCATG AGGTTGACTG ACATTTCTCT TAATGTCAAA
TBRFV Fw7       ---------- ---------- ---------- ---------- ---------- ----------
TBRFV Rv3       ---------- ---------- ---------- ---------- ---------- ----------
TBRFV Fw8       ---------- ---------- ---------- ---------- ---------- ----------
TBRFV Rv2       ---------- ---------- ---------- ---------- ---------- ----------
TBRFV Fw9       ---------- ---------- ---------- ---------- ---------- ----------
TBRFV Rv1       ---------- ---------- ---------- ---------- ---------- ----------
```

```
                ....|....| ....|....| ....|....| ....|....| ....|....| ....|....|
                      3610       3620       3630       3640       3650       3660
TBRFV_PT1       GATTGCATAT TGGATTTCTC TAAGTCTGTG GCTGCACCGA AGGATCCGAT CAAACCACTG
TBRFV_PT2       GATTGCATAT TGGATTTCTC TAAGTCTGTG GCTGCACCGA AGGATCCGAT CAAACCACTG
TBRFV Fw1       ---------- ---------- ---------- ---------- ---------- ----------
TBRFV Rv9       ---------- ---------- ---------- ---------- ---------- ----------
TBRFV Fw2       ---------- ---------- ---------- ---------- ---------- ----------
TBRFV Rv8       ---------- ---------- ---------- ---------- ---------- ----------
TBRFV Fw3       ---------- ---------- ---------- ---------- ---------- ----------
TBRFV Rv7       ---------- ---------- ---------- ---------- ---------- ----------
TBRFV Fw4       ---------- ---------- ---------- ---------- ---------- ----------
TBRFV Rv6       ---------- ---------- ---------- ---------- ---------- ----------
TBRFV Fw5       ---------- ---------- ---------- ---------- ---------- ----------
TBRFV Rv5       ---------- ---------- ---------- ---------- ---------- ----------
TBRFV Fw6       GATTGCATAT TGGATTTCTC TAAGTCTGTG GCTGCACCGA AGGATCCGAT CAAACCACTG
TBRFV Rv4       GATTGCATAT TGGATTTCTC TAAGTCTGTG GCTGCACCGA AGGATCCGAT CAAACCACTG
TBRFV Fw7       ---------- ---------- ---------- ---------- ---------- ----------
TBRFV Rv3       ---------- ---------- ---------- ---------- ---------- ----------
TBRFV Fw8       ---------- ---------- ---------- ---------- ---------- ----------
TBRFV Rv2       ---------- ---------- ---------- ---------- ---------- ----------
TBRFV Fw9       ---------- ---------- ---------- ---------- ---------- ----------
TBRFV Rv1       ---------- ---------- ---------- ---------- ---------- ----------

                ....|....| ....|....| ....|....| ....|....| ....|....| ....|....|
                      3670       3680       3690       3700       3710       3720
TBRFV_PT1       ATTCCAATGG TACGAACGGC GGCAGAAATG CCACGCCAGA CTGGACTATT GGAAAATTTG
TBRFV_PT2       ATTCCAATGG TACGAACGGC GGCAGAAATG CCACGCCAGA CTGGACTATT GGAAAATTTG
TBRFV Fw1       ---------- ---------- ---------- ---------- ---------- ----------
TBRFV Rv9       ---------- ---------- ---------- ---------- ---------- ----------
TBRFV Fw2       ---------- ---------- ---------- ---------- ---------- ----------
TBRFV Rv8       ---------- ---------- ---------- ---------- ---------- ----------
TBRFV Fw3       ---------- ---------- ---------- ---------- ---------- ----------
TBRFV Rv7       ---------- ---------- ---------- ---------- ---------- ----------
TBRFV Fw4       ---------- ---------- ---------- ---------- ---------- ----------
TBRFV Rv6       ---------- ---------- ---------- ---------- ---------- ----------
TBRFV Fw5       ---------- ---------- ---------- ---------- ---------- ----------
TBRFV Rv5       ---------- ---------- ---------- ---------- ---------- ----------
TBRFV Fw6       ATTCCAATGG TACGAACGGC GGCAGAAATG CCACGCCAGA CTGGACTATT GGAAAATTTG
TBRFV Rv4       ATTCCAATGG TACGAACGGC GGCAGAAATG CCACGCCAGA CTGGACTATT GGAAAATTTG
TBRFV Fw7       ---------- ---------- ---------- ---------- ---------- ----------
TBRFV Rv3       ---------- ---------- ---------- ---------- ---------- ----------
TBRFV Fw8       ---------- ---------- ---------- ---------- ---------- ----------
TBRFV Rv2       ---------- ---------- ---------- ---------- ---------- ----------
TBRFV Fw9       ---------- ---------- ---------- ---------- ---------- ----------
TBRFV Rv1       ---------- ---------- ---------- ---------- ---------- ----------
```

```
               ....|....| ....|....| ....|....| ....|....| ....|....| ....|....|
                    3730       3740       3750       3760       3770       3780
TBRFV_PT1      GTGGCGATGA TCAAAAGAAA CTTTAATTCA CCGGAGTTAT CAGGAATAAT CGACATTGAG
TBRFV_PT2      GTGGCGATGA TCAAAAGAAA CTTTAATTCA CCGGAGTTAT CAGGAATAAT CGACATTGAG
TBRFV Fw1      ---------- ---------- ---------- ---------- ---------- ----------
TBRFV Rv9      ---------- ---------- ---------- ---------- ---------- ----------
TBRFV Fw2      ---------- ---------- ---------- ---------- ---------- ----------
TBRFV Rv8      ---------- ---------- ---------- ---------- ---------- ----------
TBRFV Fw3      ---------- ---------- ---------- ---------- ---------- ----------
TBRFV Rv7      ---------- ---------- ---------- ---------- ---------- ----------
TBRFV Fw4      ---------- ---------- ---------- ---------- ---------- ----------
TBRFV Rv6      ---------- ---------- ---------- ---------- ---------- ----------
TBRFV Fw5      ---------- ---------- ---------- ---------- ---------- ----------
TBRFV Rv5      ---------- ---------- ---------- ---------- ---------- ----------
TBRFV Fw6      GTGGCGATGA TCAAAAGAAA CTTTAATTCA CCGGAGTTAT CAGGAATAAT CGACATTGAG
TBRFV Rv4      GTGGCGATGA TCAAAAGAAA CTTTAATTCA CCGGAGTTAT CAGGAATAAT CGACATTGAG
TBRFV Fw7      ---------- ---------- ---------- ---------- ---------- ----------
TBRFV Rv3      ---------- ---------- ---------- ---------- ---------- ----------
TBRFV Fw8      ---------- ---------- ---------- ---------- ---------- ----------
TBRFV Rv2      ---------- ---------- ---------- ---------- ---------- ----------
TBRFV Fw9      ---------- ---------- ---------- ---------- ---------- ----------
TBRFV Rv1      ---------- ---------- ---------- ---------- ---------- ----------

               ....|....| ....|....| ....|....| ....|....| ....|....| ....|....|
                    3790       3800       3810       3820       3830       3840
TBRFV_PT1      AATACTGCAT CTTTAGTAGT AGATAAATTT TTTGATAGTT ACTTGCTTAA AGAAAAAAGA
TBRFV_PT2      AATACTGCAT CTTTAGTAGT AGATAAATTT TTTGATAGTT ACTTGCTTAA AGAAAAAAGA
TBRFV Fw1      ---------- ---------- ---------- ---------- ---------- ----------
TBRFV Rv9      ---------- ---------- ---------- ---------- ---------- ----------
TBRFV Fw2      ---------- ---------- ---------- ---------- ---------- ----------
TBRFV Rv8      ---------- ---------- ---------- ---------- ---------- ----------
TBRFV Fw3      ---------- ---------- ---------- ---------- ---------- ----------
TBRFV Rv7      ---------- ---------- ---------- ---------- ---------- ----------
TBRFV Fw4      ---------- ---------- ---------- ---------- ---------- ----------
TBRFV Rv6      ---------- ---------- ---------- ---------- ---------- ----------
TBRFV Fw5      ---------- ---------- ---------- ---------- ---------- ----------
TBRFV Rv5      ---------- ---------- ---------- ---------- ---------- ----------
TBRFV Fw6      AATACTGCAT CTTTAGTAGT AGATAAATTT TTTGATAGTT ACTTGCTTAA AGAAAAAAGA
TBRFV Rv4      AATACTGCAT CTTTAGTAGT AGATAAATTT TTTGATAGTT ACTTGCTTAA AGAAAAAAGA
TBRFV Fw7      ---------- ---------- ---------- ---------- ---------- ----------
TBRFV Rv3      ---------- ---------- ---------- ---------- ---------- ----------
TBRFV Fw8      ---------- ---------- ---------- ---------- ---------- ----------
TBRFV Rv2      ---------- ---------- ---------- ---------- ---------- ----------
TBRFV Fw9      ---------- ---------- ---------- ---------- ---------- ----------
TBRFV Rv1      ---------- ---------- ---------- ---------- ---------- ----------
```

```
                ....|....| ....|....| ....|....| ....|....| ....|....| ....|....|
                       3850       3860       3870       3880       3890       3900
TBRFV_PT1       AAACCAAATA AAAATGTTTC TTTATTTTGT AGAGAGTCTC TCAATAGATG GTTAGAGAAG
TBRFV_PT2       AAACCAAATA AAAATGTTTC TTTATTTTGT AGAGAGTCTC TCAATAGATG GTTAGAGAAG
TBRFV_Fw1       ---------- ---------- ---------- ---------- ---------- ----------
TBRFV Rv9       ---------- ---------- ---------- ---------- ---------- ----------
TBRFV Fw2       ---------- ---------- ---------- ---------- ---------- ----------
TBRFV Rv8       ---------- ---------- ---------- ---------- ---------- ----------
TBRFV Fw3       ---------- ---------- ---------- ---------- ---------- ----------
TBRFV Rv7       ---------- ---------- ---------- ---------- ---------- ----------
TBRFV Fw4       ---------- ---------- ---------- ---------- ---------- ----------
TBRFV Rv6       ---------- ---------- ---------- ---------- ---------- ----------
TBRFV Fw5       ---------- ---------- ---------- ---------- ---------- ----------
TBRFV Rv5       ---------- ---------- ---------- ---------- ---------- ----------
TBRFV Fw6       AAACCAAATA AAAATGTTTC TTTATTTTGT AGAGAGTCTC TCAATAGATG GTTAGAGAAG
TBRFV Rv4       AAACCAAATA AAAATGTTTC TTTATTTTGT AGAGAGTCTC TCAATAGATG GTTAGAGAAG
TBRFV Fw7       ---------- ---------- ---------- ---------- ---------- ----------
TBRFV Rv3       ---------- ---------- ---------- ---------- ---------- ----------
TBRFV Fw8       ---------- ---------- ---------- ---------- ---------- ----------
TBRFV Rv2       ---------- ---------- ---------- ---------- ---------- ----------
TBRFV Fw9       ---------- ---------- ---------- ---------- ---------- ----------
TBRFV Rv1       ---------- ---------- ---------- ---------- ---------- ----------

                ....|....| ....|....| ....|....| ....|....| ....|....| ....|....|
                       3910       3920       3930       3940       3950       3960
TBRFV_PT1       CAGGAGCAAG TGACCATTGG TCAGCTTGCA GATTTTGATT TTGTGGATCT TCCTGCCGTT
TBRFV_PT2       CAGGAGCAAG TGACCATTGG TCAGCTTGCA GATTTTGATT TTGTGGATCT TCCTGCCGTT
TBRFV_Fw1       ---------- ---------- ---------- ---------- ---------- ----------
TBRFV Rv9       ---------- ---------- ---------- ---------- ---------- ----------
TBRFV Fw2       ---------- ---------- ---------- ---------- ---------- ----------
TBRFV Rv8       ---------- ---------- ---------- ---------- ---------- ----------
TBRFV Fw3       ---------- ---------- ---------- ---------- ---------- ----------
TBRFV Rv7       ---------- ---------- ---------- ---------- ---------- ----------
TBRFV Fw4       ---------- ---------- ---------- ---------- ---------- ----------
TBRFV Rv6       ---------- ---------- ---------- ---------- ---------- ----------
TBRFV Fw5       ---------- ---------- ---------- ---------- ---------- ----------
TBRFV Rv5       ---------- ---------- ---------- ---------- ---------- ----------
TBRFV Fw6       CAGGAGCAAG TGACCATTGG TCAGCTTGCA GATTTTGATT TTGTGGATCT TCCTGCCGTT
TBRFV Rv4       CAGGAGCAAG TGACCATTGG TCAGCTTGCA GATTTTGATT TTGTGGATCT TCCTGCCGTT
TBRFV Fw7       ---------- ---------- ---------- ---------- ---------- ----------
TBRFV Rv3       ---------- ---------- ---------- ---------- ---------- ----------
TBRFV Fw8       ---------- ---------- ---------- ---------- ---------- ----------
TBRFV Rv2       ---------- ---------- ---------- ---------- ---------- ----------
TBRFV Fw9       ---------- ---------- ---------- ---------- ---------- ----------
TBRFV Rv1       ---------- ---------- ---------- ---------- ---------- ----------
```

```
             ....|....| ....|....| ....|....| ....|....| ....|....| ....|....|
                  3970       3980       3990       4000       4010       4020
TBRFV_PT1    GATCAGTACA GGCATATGAT TAAAGCGCAA CCTAAGCAGA AGCTGGATAC ATCAATTCAA
TBRFV_PT2    GATCAGTACA GGCATATGAT TAAAGCGCAA CCTAAGCAGA AGCTGGATAC ATCAATTCAA
TBRFV Fw1    ---------- ---------- ---------- ---------- ---------- ----------
TBRFV Rv9    ---------- ---------- ---------- ---------- ---------- ----------
TBRFV Fw2    ---------- ---------- ---------- ---------- ---------- ----------
TBRFV Rv8    ---------- ---------- ---------- ---------- ---------- ----------
TBRFV Fw3    ---------- ---------- ---------- ---------- ---------- ----------
TBRFV Rv7    ---------- ---------- ---------- ---------- ---------- ----------
TBRFV Fw4    ---------- ---------- ---------- ---------- ---------- ----------
TBRFV Rv6    ---------- ---------- ---------- ---------- ---------- ----------
TBRFV Fw5    ---------- ---------- ---------- ---------- ---------- ----------
TBRFV Rv5    ---------- ---------- ---------- ---------- ---------- ----------
TBRFV Fw6    GATCAGTACA GGCATATGAT TAAAGCGCAA CCTAAGCAGA AGCTGGATAC ATCAATTCAA
TBRFV Rv4    GATCAGTACA GGCATATGAT TAAAGCGCAA CCTAAGCAGA AGCTGGATAC ATCAATTCAA
TBRFV Fw7    ---------- ---------- ---------- ---------- ---------- ----------
TBRFV Rv3    ---------- ---------- ---------- ---------- ---------- ----------
TBRFV Fw8    ---------- ---------- ---------- ---------- ---------- ----------
TBRFV Rv2    ---------- ---------- ---------- ---------- ---------- ----------
TBRFV Fw9    ---------- ---------- ---------- ---------- ---------- ----------
TBRFV Rv1    ---------- ---------- ---------- ---------- ---------- ----------
```

```
             ....|....| ....|....| ....|....| ....|....| ....|....| ....|....|
                  4030       4040       4050       4060       4070       4080
TBRFV_PT1    AGCGAATATC CGGCCTTGCA GACGATTGTG TATCATTCGA AAAAGATCAA CGCAATCTTC
TBRFV_PT2    AGCGAATATC CGGCCTTGCA GACGATTGTG TATCATTCGA AAAAGATCAA CGCAATCTTC
TBRFV Fw1    ---------- ---------- ---------- ---------- ---------- ----------
TBRFV Rv9    ---------- ---------- ---------- ---------- ---------- ----------
TBRFV Fw2    ---------- ---------- ---------- ---------- ---------- ----------
TBRFV Rv8    ---------- ---------- ---------- ---------- ---------- ----------
TBRFV Fw3    ---------- ---------- ---------- ---------- ---------- ----------
TBRFV Rv7    ---------- ---------- ---------- ---------- ---------- ----------
TBRFV Fw4    ---------- ---------- ---------- ---------- ---------- ----------
TBRFV Rv6    ---------- ---------- ---------- ---------- ---------- ----------
TBRFV Fw5    ---------- ---------- ---------- ---------- ---------- ----------
TBRFV Rv5    ---------- ---------- ---------- ---------- ---------- ----------
TBRFV Fw6    AGCGAATATC CGGCCTTGCA GACGATTGTG TATCATTCGA AAAAGATCAA CGCAATCTTC
TBRFV Rv4    AGCGAATATC CGGCCTTGCA GACGATTGTG TATCATTCGA AAAAGATCAA CGCAATCTTC
TBRFV Fw7    ---------- ---------- ---------- ---------- ---------- ----------
TBRFV Rv3    ---------- ---------- ---------- ---------- ---------- ----------
TBRFV Fw8    ---------- ---------- ---------- ---------- ---------- ----------
TBRFV Rv2    ---------- ---------- ---------- ---------- ---------- ----------
TBRFV Fw9    ---------- ---------- ---------- ---------- ---------- ----------
TBRFV Rv1    ---------- ---------- ---------- ---------- ---------- ----------
```

```
                ....|....| ....|....| ....|....| ....|....| ....|....| ....|....|
                      4090       4100       4110       4120       4130       4140
TBRFV_PT1       GGTCCTTTGT TCAGTGAGCT CACAAGGCAA ATGCTCGAAA GCATAGACTC AAGTAAGTTT
TBRFV_PT2       GGTCCTTTGT TCAGTGAGCT CACAAGGCAA ATGCTCGAAA GCATAGACTC AAGTAAGTTT
TBRFV Fw1       ---------- ---------- ---------- ---------- ---------- ----------
TBRFV Rv9       ---------- ---------- ---------- ---------- ---------- ----------
TBRFV Fw2       ---------- ---------- ---------- ---------- ---------- ----------
TBRFV Rv8       ---------- ---------- ---------- ---------- ---------- ----------
TBRFV Fw3       ---------- ---------- ---------- ---------- ---------- ----------
TBRFV Rv7       ---------- ---------- ---------- ---------- ---------- ----------
TBRFV Fw4       ---------- ---------- ---------- ---------- ---------- ----------
TBRFV Rv6       ---------- ---------- ---------- ---------- ---------- ----------
TBRFV Fw5       ---------- ---------- ---------- ---------- ---------- ----------
TBRFV Rv5       ---------- ---------- ---------- ---------- ---------- ----------
TBRFV Fw6       GGTCCTTTGT TCAGTGAGCT CACAAGGCAA ATGCTCGAAA GCATAGACTC AAGTAAGTTT
TBRFV Rv4       GGTCCTTTGT TCAGTGAGCT CACAAGGCAA ATGCTCGAAA GCATAGACTC AAGTAAGTTT
TBRFV Fw7       ---------- ---------- ---------- ---------- ---------- -----AGTTT
TBRFV Rv3       ---------- ---------- ---------- ------GA-A GCATAGACTC AAGTAAGTTT
TBRFV Fw8       ---------- ---------- ---------- ---------- ---------- ----------
TBRFV Rv2       ---------- ---------- ---------- ---------- ---------- ----------
TBRFV Fw9       ---------- ---------- ---------- ---------- ---------- ----------
TBRFV Rv1       ---------- ---------- ---------- ---------- ---------- ----------

                ....|....| ....|....| ....|....| ....|....| ....|....| ....|....|
                      4150       4160       4170       4180       4190       4200
TBRFV_PT1       TTGTTCTTTA CAAGGAAGAC GCCAGCTCAA ATTGAGGATT TCTTCGGAGA TCTCGATAGC
TBRFV_PT2       TTGTTCTTTA CAAGGAAGAC GCCAGCTCAA ATTGAGAATT TCTTCGGAGA TCTCGATAGC
TBRFV Fw1       ---------- ---------- ---------- ---------- ---------- ----------
TBRFV Rv9       ---------- ---------- ---------- ---------- ---------- ----------
TBRFV Fw2       ---------- ---------- ---------- ---------- ---------- ----------
TBRFV Rv8       ---------- ---------- ---------- ---------- ---------- ----------
TBRFV Fw3       ---------- ---------- ---------- ---------- ---------- ----------
TBRFV Rv7       ---------- ---------- ---------- ---------- ---------- ----------
TBRFV Fw4       ---------- ---------- ---------- ---------- ---------- ----------
TBRFV Rv6       ---------- ---------- ---------- ---------- ---------- ----------
TBRFV Fw5       ---------- ---------- ---------- ---------- ---------- ----------
TBRFV Rv5       ---------- ---------- ---------- ---------- ---------- ----------
TBRFV Fw6       TTGTTCTTTA CAAGGAAGAC GCCAGCTCAA ATTGAGAATT TCTTCGGAGA TCTCGATAGC
TBRFV Rv4       TTGTTCTTTA CAAGGAAGAC GCCAGCTCAA ATTGAGAA-- ---------- ----------
TBRFV Fw7       TTGTTCTTTA CAAGGAAGAC GCCAGCTCAA ATTGAGAATT TCTTCGGAGA TCTCGATAGC
TBRFV Rv3       TTGTTCTTTA CAAGGAAGAC GCCAGCTCAA ATTGAGAATT TCTTCGGAGA TCTCGATAGC
TBRFV Fw8       ---------- ---------- ---------- ---------- ---------- ----------
TBRFV Rv2       ---------- ---------- ---------- ---------- ---------- ----------
TBRFV Fw9       ---------- ---------- ---------- ---------- ---------- ----------
TBRFV Rv1       ---------- ---------- ---------- ---------- ---------- ----------
```

```
                ....|....| ....|....| ....|....| ....|....| ....|....| ....|....|
                       4210       4220       4230       4240       4250       4260
TBRFV_PT1       CATGTCCCTA TGGATATCTT GGAGTTGGAT ATTTCGAAGT ATGACAAATC TCAGAACGAG
TBRFV_PT2       CATGTCCCTA TGGATATCTT GGAGTTGGAT ATTTCGAAGT ATGACAAATC TCAGAACGAG
TBRFV Fw1       ---------- ---------- ---------- ---------- ---------- ----------
TBRFV Rv9       ---------- ---------- ---------- ---------- ---------- ----------
TBRFV Fw2       ---------- ---------- ---------- ---------- ---------- ----------
TBRFV Rv8       ---------- ---------- ---------- ---------- ---------- ----------
TBRFV Fw3       ---------- ---------- ---------- ---------- ---------- ----------
TBRFV Rv7       ---------- ---------- ---------- ---------- ---------- ----------
TBRFV Fw4       ---------- ---------- ---------- ---------- ---------- ----------
TBRFV Rv6       ---------- ---------- ---------- ---------- ---------- ----------
TBRFV Fw5       ---------- ---------- ---------- ---------- ---------- ----------
TBRFV Rv5       ---------- ---------- ---------- ---------- ---------- ----------
TBRFV Fw6       CATGTCCCTA TG-------- ---------- ---------- ---------- ----------
TBRFV Rv4       ---------- ---------- ---------- ---------- ---------- ----------
TBRFV Fw7       CATGTCCCTA TGGATATCTT GGAGTTGGAT ATTTCGAAGT ATGACAAATC TCAGAACGAG
TBRFV Rv3       CATGTCCCTA TGGATATCTT GGAGTTGGAT ATTTCGAAGT ATGACAAATC TCAGAACGAG
TBRFV Fw8       ---------- ---------- ---------- ---------- ---------- ----------
TBRFV Rv2       ---------- ---------- ---------- ---------- ---------- ----------
TBRFV Fw9       ---------- ---------- ---------- ---------- ---------- ----------
TBRFV Rv1       ---------- ---------- ---------- ---------- ---------- ----------
```

```
                ....|....| ....|....| ....|....| ....|....| ....|....| ....|....|
                       4270       4280       4290       4300       4310       4320
TBRFV_PT1       TTCCACTGTG CAGTAGAGTA TGAAATATGG AGAAGACTTG GATTAGAAGA TTTTCTGGGA
TBRFV_PT2       TTCCACTGTG CAGTAGAGTA TGAAATATGG AGAAGACTTG GATTAGAAGA TTTTCTGGGA
TBRFV Fw1       ---------- ---------- ---------- ---------- ---------- ----------
TBRFV Rv9       ---------- ---------- ---------- ---------- ---------- ----------
TBRFV Fw2       ---------- ---------- ---------- ---------- ---------- ----------
TBRFV Rv8       ---------- ---------- ---------- ---------- ---------- ----------
TBRFV Fw3       ---------- ---------- ---------- ---------- ---------- ----------
TBRFV Rv7       ---------- ---------- ---------- ---------- ---------- ----------
TBRFV Fw4       ---------- ---------- ---------- ---------- ---------- ----------
TBRFV Rv6       ---------- ---------- ---------- ---------- ---------- ----------
TBRFV Fw5       ---------- ---------- ---------- ---------- ---------- ----------
TBRFV Rv5       ---------- ---------- ---------- ---------- ---------- ----------
TBRFV Fw6       ---------- ---------- ---------- ---------- ---------- ----------
TBRFV Rv4       ---------- ---------- ---------- ---------- ---------- ----------
TBRFV Fw7       TTCCACTGTG CAGTAGAGTA TGAAATATGG AGAAGACTTG GATTAGAAGA TTTTCTGGGA
TBRFV Rv3       TTCCACTGTG CAGTAGAGTA TGAAATATGG AGAAGACTTG GATTAGAAGA TTTTCTGGGA
TBRFV Fw8       ---------- ---------- ---------- ---------- ---------- ----------
TBRFV Rv2       ---------- ---------- ---------- ---------- ---------- ----------
TBRFV Fw9       ---------- ---------- ---------- ---------- ---------- ----------
TBRFV Rv1       ---------- ---------- ---------- ---------- ---------- ----------
```

```
             ....|....| ....|....| ....|....| ....|....| ....|....| ....|....|
                   4330       4340       4350       4360       4370       4380
TBRFV_PT1    GAAGTTTGGA AACAAGGCCA CAGGAAAACT ACTCTTAAAG ATTACACAGC TGGTATTAAA
TBRFV_PT2    GAAGTTTGGA AACAAGGCCA CAGGAAAACT ACTCTTAAAG ATTACACAGC TGGTATTAAA
TBRFV Fw1    ---------- ---------- ---------- ---------- ---------- ----------
TBRFV Rv9    ---------- ---------- ---------- ---------- ---------- ----------
TBRFV Fw2    ---------- ---------- ---------- ---------- ---------- ----------
TBRFV Rv8    ---------- ---------- ---------- ---------- ---------- ----------
TBRFV Fw3    ---------- ---------- ---------- ---------- ---------- ----------
TBRFV Rv7    ---------- ---------- ---------- ---------- ---------- ----------
TBRFV Fw4    ---------- ---------- ---------- ---------- ---------- ----------
TBRFV Rv6    ---------- ---------- ---------- ---------- ---------- ----------
TBRFV Fw5    ---------- ---------- ---------- ---------- ---------- ----------
TBRFV Rv5    ---------- ---------- ---------- ---------- ---------- ----------
TBRFV Fw6    ---------- ---------- ---------- ---------- ---------- ----------
TBRFV Rv4    ---------- ---------- ---------- ---------- ---------- ----------
TBRFV Fw7    GAAGTTTGGA AACAAGGCCA CAGGAAAACT ACTCTTAAAG ATTACACAGC TGGTATTAAA
TBRFV Rv3    GAAGTTTGGA AACAAGGCCA CAGGAAAACT ACTCTTAAAG ATTACACAGC TGGTATTAAA
TBRFV Fw8    ---------- ---------- ---------- ---------- ---------- ----------
TBRFV Rv2    ---------- ---------- ---------- ---------- ---------- ----------
TBRFV Fw9    ---------- ---------- ---------- ---------- ---------- ----------
TBRFV Rv1    ---------- ---------- ---------- ---------- ---------- ----------
```

```
             ....|....| ....|....| ....|....| ....|....| ....|....| ....|....|
                   4390       4400       4410       4420       4430       4440
TBRFV_PT1    ACGTGTTTAT GGTACCAGAG AAAGAGTGGG GACGTTACAA CATTCATCGG TAATACGGTG
TBRFV_PT2    ACGTGTTTAT GGTACCAGAG AAAGAGTGGG GACGTTACAA CATTCATCGG TAATACGGTG
TBRFV Fw1    ---------- ---------- ---------- ---------- ---------- ----------
TBRFV Rv9    ---------- ---------- ---------- ---------- ---------- ----------
TBRFV Fw2    ---------- ---------- ---------- ---------- ---------- ----------
TBRFV Rv8    ---------- ---------- ---------- ---------- ---------- ----------
TBRFV Fw3    ---------- ---------- ---------- ---------- ---------- ----------
TBRFV Rv7    ---------- ---------- ---------- ---------- ---------- ----------
TBRFV Fw4    ---------- ---------- ---------- ---------- ---------- ----------
TBRFV Rv6    ---------- ---------- ---------- ---------- ---------- ----------
TBRFV Fw5    ---------- ---------- ---------- ---------- ---------- ----------
TBRFV Rv5    ---------- ---------- ---------- ---------- ---------- ----------
TBRFV Fw6    ---------- ---------- ---------- ---------- ---------- ----------
TBRFV Rv4    ---------- ---------- ---------- ---------- ---------- ----------
TBRFV Fw7    ACGTGTTTAT GGTACCAGAG AAAGAGTGGG GACGTTACAA CATTCATCGG TAATACGGTG
TBRFV Rv3    ACGTGTTTAT GGTACCAGAG AAAGAGTGGG GACGTTACAA CATTCATCGG TAATACGGTG
TBRFV Fw8    ---------- ---------- ---------- ---------- ---------- ----------
TBRFV Rv2    ---------- ---------- ---------- ---------- ---------- ----------
TBRFV Fw9    ---------- ---------- ---------- ---------- ---------- ----------
TBRFV Rv1    ---------- ---------- ---------- ---------- ---------- ----------
```

```
               ....|....| ....|....| ....|....| ....|....| ....|....| ....|....|
                      4450       4460       4470       4480       4490       4500
TBRFV_PT1      ATTATTGCTG CTTGTTTAGC TTCCATGTTG CCCATGGAGA AAATAATCAA AGGTGCATTT
TBRFV_PT2      ATTATTGCTG CTTGTTTAGC TTCCATGTTG CCCATGGAGA AAATAATCAA AGGTGCATTT
TBRFV_Fw1      ---------- ---------- ---------- ---------- ---------- ----------
TBRFV Rv9      ---------- ---------- ---------- ---------- ---------- ----------
TBRFV Fw2      ---------- ---------- ---------- ---------- ---------- ----------
TBRFV Rv8      ---------- ---------- ---------- ---------- ---------- ----------
TBRFV Fw3      ---------- ---------- ---------- ---------- ---------- ----------
TBRFV Rv7      ---------- ---------- ---------- ---------- ---------- ----------
TBRFV Fw4      ---------- ---------- ---------- ---------- ---------- ----------
TBRFV Rv6      ---------- ---------- ---------- ---------- ---------- ----------
TBRFV Fw5      ---------- ---------- ---------- ---------- ---------- ----------
TBRFV Rv5      ---------- ---------- ---------- ---------- ---------- ----------
TBRFV Fw6      ---------- ---------- ---------- ---------- ---------- ----------
TBRFV Rv4      ---------- ---------- ---------- ---------- ---------- ----------
TBRFV Fw7      ATTATTGCTG CTTGTTTAGC TTCCATGTTG CCCATGGAGA AAATAATCAA AGGTGCATTT
TBRFV Rv3      ATTATTGCTG CTTGTTTAGC TTCCATGTTG CCCATGGAGA AAATAATCAA AGGTGCATTT
TBRFV Fw8      ---------- ---------- ---------- ---------- ---------- ----------
TBRFV Rv2      ---------- ---------- ---------- ---------- ---------- ----------
TBRFV Fw9      ---------- ---------- ---------- ---------- ---------- ----------
TBRFV Rv1      ---------- ---------- ---------- ---------- ---------- ----------

               ....|....| ....|....| ....|....| ....|....| ....|....| ....|....|
                      4510       4520       4530       4540       4550       4560
TBRFV_PT1      TGCGGAGATG ACAGTTTACT ATACTTCCCA AAAGGTTGTG AGTTTCCTGA CATACAGCAT
TBRFV_PT2      TGCGGAGATG ACAGTTTACT ATACTTCCCA AAAGGTTGTG AGTTTCCTGA CATACAGCAT
TBRFV_Fw1      ---------- ---------- ---------- ---------- ---------- ----------
TBRFV Rv9      ---------- ---------- ---------- ---------- ---------- ----------
TBRFV Fw2      ---------- ---------- ---------- ---------- ---------- ----------
TBRFV Rv8      ---------- ---------- ---------- ---------- ---------- ----------
TBRFV Fw3      ---------- ---------- ---------- ---------- ---------- ----------
TBRFV Rv7      ---------- ---------- ---------- ---------- ---------- ----------
TBRFV Fw4      ---------- ---------- ---------- ---------- ---------- ----------
TBRFV Rv6      ---------- ---------- ---------- ---------- ---------- ----------
TBRFV Fw5      ---------- ---------- ---------- ---------- ---------- ----------
TBRFV Rv5      ---------- ---------- ---------- ---------- ---------- ----------
TBRFV Fw6      ---------- ---------- ---------- ---------- ---------- ----------
TBRFV Rv4      ---------- ---------- ---------- ---------- ---------- ----------
TBRFV Fw7      TGCGGAGATG ACAGTTTACT ATACTTCCCA AAAGGTTGTG AGTTTCCTGA CATACAGCAT
TBRFV Rv3      TGCGGAGATG ACAGTTTACT ATACTTCCCA AAAGGTTGTG AGTTTCCTGA CATACAGCAT
TBRFV Fw8      ---------- ---------- ---------- ---------- ---------- ----------
TBRFV Rv2      ---------- ---------- ---------- ---------- ---------- ----------
TBRFV Fw9      ---------- ---------- ---------- ---------- ---------- ----------
TBRFV Rv1      ---------- ---------- ---------- ---------- ---------- ----------
```

```
               ....|....| ....|....| ....|....| ....|....| ....|....| ....|....|
                      4570       4580       4590       4600       4610       4620
TBRFV_PT1      ACAGCTAACC TTATGTGGAA TTTCGAGGCT AAGCTATTCA GAAAGCAGTA TGGTTATTTC
TBRFV_PT2      ACAGCTAACC TTATGTGGAA TTTCGAGGCT AAGCTATTCA GAAAGCAGTA TGGTTATTTC
TBRFV Fw1      ---------- ---------- ---------- ---------- ---------- ----------
TBRFV Rv9      ---------- ---------- ---------- ---------- ---------- ----------
TBRFV Fw2      ---------- ---------- ---------- ---------- ---------- ----------
TBRFV Rv8      ---------- ---------- ---------- ---------- ---------- ----------
TBRFV Fw3      ---------- ---------- ---------- ---------- ---------- ----------
TBRFV Rv7      ---------- ---------- ---------- ---------- ---------- ----------
TBRFV Fw4      ---------- ---------- ---------- ---------- ---------- ----------
TBRFV Rv6      ---------- ---------- ---------- ---------- ---------- ----------
TBRFV Fw5      ---------- ---------- ---------- ---------- ---------- ----------
TBRFV Rv5      ---------- ---------- ---------- ---------- ---------- ----------
TBRFV Fw6      ---------- ---------- ---------- ---------- ---------- ----------
TBRFV Rv4      ---------- ---------- ---------- ---------- ---------- ----------
TBRFV Fw7      ACAGCTAACC TTATGTGGAA TTTCGAGGCT AAGCTATTCA GAAAGCAGTA TGGTTATTTC
TBRFV Rv3      ACAGCTAACC TTATGTGGAA TTTCGAGGCT AAGCTATTCA GAAAGCAGTA TGGTTATTTC
TBRFV Fw8      ---------- ---------- ---------- ---------- ---------- ----------
TBRFV Rv2      ---------- ---------- ---------- ---------- ---------- ----------
TBRFV Fw9      ---------- ---------- ---------- ---------- ---------- ----------
TBRFV Rv1      ---------- ---------- ---------- ---------- ---------- ----------
```

```
               ....|....| ....|....| ....|....| ....|....| ....|....| ....|....|
                      4630       4640       4650       4660       4670       4680
TBRFV_PT1      TGTGGAAGGT ACGTGATACA TCATGACAGA GGGTGTATTG TTTATTATGA CCCTTTGAAG
TBRFV_PT2      TGTGGAAGGT ACGTGATACA TCATGACAGA GGGTGTATTG TTTATTATGA CCCTTTGAAG
TBRFV Fw1      ---------- ---------- ---------- ---------- ---------- ----------
TBRFV Rv9      ---------- ---------- ---------- ---------- ---------- ----------
TBRFV Fw2      ---------- ---------- ---------- ---------- ---------- ----------
TBRFV Rv8      ---------- ---------- ---------- ---------- ---------- ----------
TBRFV Fw3      ---------- ---------- ---------- ---------- ---------- ----------
TBRFV Rv7      ---------- ---------- ---------- ---------- ---------- ----------
TBRFV Fw4      ---------- ---------- ---------- ---------- ---------- ----------
TBRFV Rv6      ---------- ---------- ---------- ---------- ---------- ----------
TBRFV Fw5      ---------- ---------- ---------- ---------- ---------- ----------
TBRFV Rv5      ---------- ---------- ---------- ---------- ---------- ----------
TBRFV Fw6      ---------- ---------- ---------- ---------- ---------- ----------
TBRFV Rv4      ---------- ---------- ---------- ---------- ---------- ----------
TBRFV Fw7      TGTGGAAGGT ACGTGATACA TCATGACAGA GGGTGTATTG TTTATTATGA CCCTTTGAAG
TBRFV Rv3      TGTGGAAGGT ACGTGATACA TCATGACAGA GGGTGTATTG TTTATTATGA CCCTTTGAAG
TBRFV Fw8      ---------- ---------- ---------- ---------- ---------- ----------
TBRFV Rv2      ---------- ---------- ---------- ---------- ---------- ----------
TBRFV Fw9      ---------- ---------- ---------- ---------- ---------- ----------
TBRFV Rv1      ---------- ---------- ---------- ---------- ---------- ----------
```

```
             ....|....| ....|....| ....|....| ....|....| ....|....| ....|....|
                  4690       4700       4710       4720       4730       4740
TBRFV_PT1    TTGATTTCTA AACTTGGTGC TAAACACATC AAGGATTGGG ATCACTTAGA AGAGTTCAGA
TBRFV_PT2    TTGATTTCTA AACTTGGTGC TAAACACATC AAGGATTGGG ATCACTTAGA AGAGTTCAGA
TBRFV Fw1    ---------- ---------- ---------- ---------- ---------- ----------
TBRFV Rv9    ---------- ---------- ---------- ---------- ---------- ----------
TBRFV Fw2    ---------- ---------- ---------- ---------- ---------- ----------
TBRFV Rv8    ---------- ---------- ---------- ---------- ---------- ----------
TBRFV Fw3    ---------- ---------- ---------- ---------- ---------- ----------
TBRFV Rv7    ---------- ---------- ---------- ---------- ---------- ----------
TBRFV Fw4    ---------- ---------- ---------- ---------- ---------- ----------
TBRFV Rv6    ---------- ---------- ---------- ---------- ---------- ----------
TBRFV Fw5    ---------- ---------- ---------- ---------- ---------- ----------
TBRFV Rv5    ---------- ---------- ---------- ---------- ---------- ----------
TBRFV Fw6    ---------- ---------- ---------- ---------- ---------- ----------
TBRFV Rv4    ---------- ---------- ---------- ---------- ---------- ----------
TBRFV Fw7    TTGATTTCTA AACTTGGTGC TAAACACATC AAGGATTGGG ATCACTTAGA AGAGTTCAGA
TBRFV Rv3    TTGATTTCTA AACTTGGTGC TAAACACATC AAGGATTGGG ATCACTTAGA AGAGTTCAGA
TBRFV Fw8    ---------- ---------- ---------- ---------- ---------- ----------
TBRFV Rv2    ---------- ---------- ---------- ---------- ---------- ----------
TBRFV Fw9    ---------- ---------- ---------- ---------- ---------- ----------
TBRFV Rv1    ---------- ---------- ---------- ---------- ---------- ----------
```

~

```
             ....|....| ....|....| ....|....| ....|....| ....|....| ....|....|
                  4750       4760       4770       4780       4790       4800
TBRFV_PT1    AGATCCCTTT GTGATGTTGC AAATTCGTTG AACAACTGTG CGTATTACAC GCAGTTGGAC
TBRFV_PT2    AGATCCCTTT GTGATGTTGC AAATTCGTTG AACAACTGTG CGTATTACAC GCAGTTGGAC
TBRFV Fw1    ---------- ---------- ---------- ---------- ---------- ----------
TBRFV Rv9    ---------- ---------- ---------- ---------- ---------- ----------
TBRFV Fw2    ---------- ---------- ---------- ---------- ---------- ----------
TBRFV Rv8    ---------- ---------- ---------- ---------- ---------- ----------
TBRFV Fw3    ---------- ---------- ---------- ---------- ---------- ----------
TBRFV Rv7    ---------- ---------- ---------- ---------- ---------- ----------
TBRFV Fw4    ---------- ---------- ---------- ---------- ---------- ----------
TBRFV Rv6    ---------- ---------- ---------- ---------- ---------- ----------
TBRFV Fw5    ---------- ---------- ---------- ---------- ---------- ----------
TBRFV Rv5    ---------- ---------- ---------- ---------- ---------- ----------
TBRFV Fw6    ---------- ---------- ---------- ---------- ---------- ----------
TBRFV Rv4    ---------- ---------- ---------- ---------- ---------- ----------
TBRFV Fw7    AGATCCCTTT GTGATGTTGC AAATTCGTTG AACAACTGTG CGTATTACAC GCAGTTGGAC
TBRFV Rv3    AGATCCCTTT GTGATGTTGC AAATTCGTTG AACAACTGTG CGTATTACAC GCAGTTGGAC
TBRFV Fw8    ---------- ---------- ---------- ---------- ---------- ----------
TBRFV Rv2    ---------- ---------- ---------- ---------- ---------- ----------
TBRFV Fw9    ---------- ---------- ---------- ---------- ---------- ----------
TBRFV Rv1    ---------- ---------- ---------- ---------- ---------- ----------
```

```
            ....|....| ....|....| ....|....| ....|....| ....|....| ....|....|
                  4810       4820       4830       4840       4850       4860
TBRFV_PT1   GACGCTGTGA GTGAGGTCCA TAAAACCGCA CCCCCGGGTT CGTTTGTATA TAAAAGTTTA
TBRFV_PT2   GACGCTGTGA GTGAGGTCCA TAAAACCGCA CCCCCGGGTT CGTTTGTATA TAAAAGTTTA
TBRFV_Fw1   ---------- ---------- ---------- ---------- ---------- ----------
TBRFV_Rv9   ---------- ---------- ---------- ---------- ---------- ----------
TBRFV_Fw2   ---------- ---------- ---------- ---------- ---------- ----------
TBRFV_Rv8   ---------- ---------- ---------- ---------- ---------- ----------
TBRFV_Fw3   ---------- ---------- ---------- ---------- ---------- ----------
TBRFV_Rv7   ---------- ---------- ---------- ---------- ---------- ----------
TBRFV_Fw4   ---------- ---------- ---------- ---------- ---------- ----------
TBRFV_Rv6   ---------- ---------- ---------- ---------- ---------- ----------
TBRFV_Fw5   ---------- ---------- ---------- ---------- ---------- ----------
TBRFV_Rv5   ---------- ---------- ---------- ---------- ---------- ----------
TBRFV_Fw6   ---------- ---------- ---------- ---------- ---------- ----------
TBRFV_Rv4   ---------- ---------- ---------- ---------- ---------- ----------
TBRFV_Fw7   GACGCTGTGA GTGAGGTCCA TAAAACCGCA CCCCCGGGTT CGTTTGTATA TAAAAGTTTA
TBRFV_Rv3   GACGCTGTGA GTGAGGTCCA TAAAACCGCA CCCCCGGGTT CGTTTGTATA TAAAAGTTTA
TBRFV_Fw8   ---------- ---------- ---------- ---------- ---------- --AAAGTTTA
TBRFV_Rv2   ---------- -TGAGGTCCA TAAAACCGCA CCCCCGGGTT CGTTTGTATA TAAAAGTTTA
TBRFV_Fw9   ---------- ---------- ---------- ---------- ---------- ----------
TBRFV_Rv1   ---------- ---------- ---------- ---------- ---------- ----------

            ....|....| ....|....| ....|....| ....|....| ....|....| ....|....|
                  4870       4880       4890       4900       4910       4920
TBRFV_PT1   GTTAAATATC TGTCCGATAA GGTTCTTTTT AGAAGTTTGT TTATAGATGG CTCTTGTTAA
TBRFV_PT2   GTTAAATATC TGTCCGATAA GGTTCTTTTT AGAAGTTTGT TTATAGATGG CTCTTGTTAA
TBRFV_Fw1   ---------- ---------- ---------- ---------- ---------- ----------
TBRFV_Rv9   ---------- ---------- ---------- ---------- ---------- ----------
TBRFV_Fw2   ---------- ---------- ---------- ---------- ---------- ----------
TBRFV_Rv8   ---------- ---------- ---------- ---------- ---------- ----------
TBRFV_Fw3   ---------- ---------- ---------- ---------- ---------- ----------
TBRFV_Rv7   ---------- ---------- ---------- ---------- ---------- ----------
TBRFV_Fw4   ---------- ---------- ---------- ---------- ---------- ----------
TBRFV_Rv6   ---------- ---------- ---------- ---------- ---------- ----------
TBRFV_Fw5   ---------- ---------- ---------- ---------- ---------- ----------
TBRFV_Rv5   ---------- ---------- ---------- ---------- ---------- ----------
TBRFV_Fw6   ---------- ---------- ---------- ---------- ---------- ----------
TBRFV_Rv4   ---------- ---------- ---------- ---------- ---------- ----------
TBRFV_Fw7   GTTAAATATC TGTCCGATAA GGTTCTTTTT AGAAGTTTGT TTATAGATGG ----------
TBRFV_Rv3   GTTAAATATC TGTCCGA--- ---------- ---------- ---------- ----------
TBRFV_Fw8   GTTAA-TATC TGTCCGATAA GGTTCTTTTT AGAAGTTTGT TTATAGATGG CTCTTGTTAA
TBRFV_Rv2   GTTAAATATC TGTCCGATAA GGTTCTTTTT AGAAGTTTGT TTATAGATGG CTCTTGTTAA
TBRFV_Fw9   ---------- ---------- ---------- ---------- ---------- ----------
TBRFV_Rv1   ---------- ---------- ---------- ---------- ---------- ----------
```

```
               ....|....| ....|....| ....|....| ....|....| ....|....| ....|....|
                      4930       4940       4950       4960       4970       4980
TBRFV_PT1      GGGTAAAGTC AATATTAATG AGTTCATAGA CTTGTCAAAA TCAGAAAAAT TTCTTCCGTC
TBRFV_PT2      GGGTAAAGTC AATATTAATG AGTTCATAGA CTTGTCAAAA TCAGAAAAAT TTCTTCCGTC
TBRFV Fw1      ---------- ---------- ---------- ---------- ---------- ----------
TBRFV Rv9      ---------- ---------- ---------- ---------- ---------- ----------
TBRFV Fw2      ---------- ---------- ---------- ---------- ---------- ----------
TBRFV Rv8      ---------- ---------- ---------- ---------- ---------- ----------
TBRFV Fw3      ---------- ---------- ---------- ---------- ---------- ----------
TBRFV Rv7      ---------- ---------- ---------- ---------- ---------- ----------
TBRFV Fw4      ---------- ---------- ---------- ---------- ---------- ----------
TBRFV Rv6      ---------- ---------- ---------- ---------- ---------- ----------
TBRFV Fw5      ---------- ---------- ---------- ---------- ---------- ----------
TBRFV Rv5      ---------- ---------- ---------- ---------- ---------- ----------
TBRFV Fw6      ---------- ---------- ---------- ---------- ---------- ----------
TBRFV Rv4      ---------- ---------- ---------- ---------- ---------- ----------
TBRFV Fw7      ---------- ---------- ---------- ---------- ---------- ----------
TBRFV Rv3      ---------- ---------- ---------- ---------- ---------- ----------
TBRFV Fw8      GGGTAAAGTC AATATTAATG AGTTCATAGA CTTGTCAAAA TCAGAAAAAT TTCTTCCGTC
TBRFV Rv2      GGGTAAAGTC AATATTAATG AGTTCATAGA CTTGTCAAAA TCAGAAAAAT TTCTTCCGTC
TBRFV Fw9      ---------- ---------- ---------- ---------- ---------- ----------
TBRFV Rv1      ---------- ---------- ---------- ---------- ---------- ----------
```

```
               ....|....| ....|....| ....|....| ....|....| ....|....| ....|....|
                      4990       5000       5010       5020       5030       5040
TBRFV_PT1      TATGTTCACA CCTGTTAAGA GTGTCATGAT CTCCAAGGTT GATAAGATAT TGGTTCATGA
TBRFV_PT2      TATGTTCACA CCTGTTAAGA GTGTCATGAT CTCCAAGGTT GATAAGATAT TGGTTCATGA
TBRFV Fw1      ---------- ---------- ---------- ---------- ---------- ----------
TBRFV Rv9      ---------- ---------- ---------- ---------- ---------- ----------
TBRFV Fw2      ---------- ---------- ---------- ---------- ---------- ----------
TBRFV Rv8      ---------- ---------- ---------- ---------- ---------- ----------
TBRFV Fw3      ---------- ---------- ---------- ---------- ---------- ----------
TBRFV Rv7      ---------- ---------- ---------- ---------- ---------- ----------
TBRFV Fw4      ---------- ---------- ---------- ---------- ---------- ----------
TBRFV Rv6      ---------- ---------- ---------- ---------- ---------- ----------
TBRFV Fw5      ---------- ---------- ---------- ---------- ---------- ----------
TBRFV Rv5      ---------- ---------- ---------- ---------- ---------- ----------
TBRFV Fw6      ---------- ---------- ---------- ---------- ---------- ----------
TBRFV Rv4      ---------- ---------- ---------- ---------- ---------- ----------
TBRFV Fw7      ---------- ---------- ---------- ---------- ---------- ----------
TBRFV Rv3      ---------- ---------- ---------- ---------- ---------- ----------
TBRFV Fw8      TATGTTCACA CCTGTTAAGA GTGTCATGAT CTCCAAGGTT GATAAGATAT TGGTTCATGA
TBRFV Rv2      TATGTTCACA CCTGTTAAGA GTGTCATGAT CTCCAAGGTT GATAAGATAT TGGTTCATGA
TBRFV Fw9      ---------- ---------- ---------- ---------- ---------- ----------
TBRFV Rv1      ---------- ---------- ---------- ---------- ---------- ----------
```

```
                ....|....| ....|....| ....|....| ....|....| ....|....| ....|....|
                      5050       5060       5070       5080       5090       5100
TBRFV_PT1       AGATGAATCT TTGTCCGAAG TCAATTTACT CAAAGGTGTA AAACTCATTG ATGGTGGCTA
TBRFV_PT2       AGATGAATCT TTGTCCGAAG TCAATTTACT CAAAGGTGTA AAACTCATTG ATGGTGGCTA
TBRFV Fw1       ---------- ---------- ---------- ---------- ---------- ----------
TBRFV Rv9       ---------- ---------- ---------- ---------- ---------- ----------
TBRFV Fw2       ---------- ---------- ---------- ---------- ---------- ----------
TBRFV Rv8       ---------- ---------- ---------- ---------- ---------- ----------
TBRFV Fw3       ---------- ---------- ---------- ---------- ---------- ----------
TBRFV Rv7       ---------- ---------- ---------- ---------- ---------- ----------
TBRFV Fw4       ---------- ---------- ---------- ---------- ---------- ----------
TBRFV Rv6       ---------- ---------- ---------- ---------- ---------- ----------
TBRFV Fw5       ---------- ---------- ---------- ---------- ---------- ----------
TBRFV Rv5       ---------- ---------- ---------- ---------- ---------- ----------
TBRFV Fw6       ---------- ---------- ---------- ---------- ---------- ----------
TBRFV Rv4       ---------- ---------- ---------- ---------- ---------- ----------
TBRFV Fw7       ---------- ---------- ---------- ---------- ---------- ----------
TBRFV Rv3       ---------- ---------- ---------- ---------- ---------- ----------
TBRFV Fw8       AGATGAATCT TTGTCCGAAG TCAATTTACT CAAAGGTGTA AAACTCATTG ATGGTGGCTA
TBRFV Rv2       AGATGAATCT TTGTCCGAAG TCAATTTACT CAAAGGTGTA AAACTCATTG ATGGTGGCTA
TBRFV Fw9       ---------- ---------- ---------- ---------- ---------- ----------
TBRFV Rv1       ---------- ---------- ---------- ---------- ---------- ----------
```

```
                ....|....| ....|....| ....|....| ....|....| ....|....| ....|....|
                      5110       5120       5130       5140       5150       5160
TBRFV_PT1       TGTACATCTT GCTGGTCTTG TAGTGACAGG TGAATGGAAT TTGCCAGATA ATTGTCGCGG
TBRFV_PT2       TGTACATCTT GCTGGTCTTG TAGTGACAGG TGAATGGAAT TTGCCAGATA ATTGTCGCGG
TBRFV Fw1       ---------- ---------- ---------- ---------- ---------- ----------
TBRFV Rv9       ---------- ---------- ---------- ---------- ---------- ----------
TBRFV Fw2       ---------- ---------- ---------- ---------- ---------- ----------
TBRFV Rv8       ---------- ---------- ---------- ---------- ---------- ----------
TBRFV Fw3       ---------- ---------- ---------- ---------- ---------- ----------
TBRFV Rv7       ---------- ---------- ---------- ---------- ---------- ----------
TBRFV Fw4       ---------- ---------- ---------- ---------- ---------- ----------
TBRFV Rv6       ---------- ---------- ---------- ---------- ---------- ----------
TBRFV Fw5       ---------- ---------- ---------- ---------- ---------- ----------
TBRFV Rv5       ---------- ---------- ---------- ---------- ---------- ----------
TBRFV Fw6       ---------- ---------- ---------- ---------- ---------- ----------
TBRFV Rv4       ---------- ---------- ---------- ---------- ---------- ----------
TBRFV Fw7       ---------- ---------- ---------- ---------- ---------- ----------
TBRFV Rv3       ---------- ---------- ---------- ---------- ---------- ----------
TBRFV Fw8       TGTACATCTT GCTGGTCTTG TAGTGACAGG TGAATGGAAT TTGCCAGATA ATTGTCGCGG
TBRFV Rv2       TGTACATCTT GCTGGTCTTG TAGTGACAGG TGAATGGAAT TTGCCAGATA ATTGTCGCGG
TBRFV Fw9       ---------- ---------- ---------- ---------- ---------- ----------
TBRFV Rv1       ---------- ---------- ---------- ---------- ---------- ----------
```

```
              ....|....| ....|....| ....|....| ....|....| ....|....| ....|....|
                    5170       5180       5190       5200       5210       5220
TBRFV_PT1     TGGTGTCAGT GTCTGTTTGG TCGATAAGAG AATGGAGAGA GCGGACGAGG CAACTCTTGC
TBRFV_PT2     TGGTGTCAGT GTCTGTTTGG TCGATAAGAG AATGGAGAGA GCGGACGAGG CAACTCTTGC
TBRFV_Fw1     ---------- ---------- ---------- ---------- ---------- ----------
TBRFV_Rv9     ---------- ---------- ---------- ---------- ---------- ----------
TBRFV_Fw2     ---------- ---------- ---------- ---------- ---------- ----------
TBRFV_Rv8     ---------- ---------- ---------- ---------- ---------- ----------
TBRFV_Fw3     ---------- ---------- ---------- ---------- ---------- ----------
TBRFV_Rv7     ---------- ---------- ---------- ---------- ---------- ----------
TBRFV_Fw4     ---------- ---------- ---------- ---------- ---------- ----------
TBRFV_Rv6     ---------- ---------- ---------- ---------- ---------- ----------
TBRFV_Fw5     ---------- ---------- ---------- ---------- ---------- ----------
TBRFV_Rv5     ---------- ---------- ---------- ---------- ---------- ----------
TBRFV_Fw6     ---------- ---------- ---------- ---------- ---------- ----------
TBRFV_Rv4     ---------- ---------- ---------- ---------- ---------- ----------
TBRFV_Fw7     ---------- ---------- ---------- ---------- ---------- ----------
TBRFV_Rv3     ---------- ---------- ---------- ---------- ---------- ----------
TBRFV_Fw8     TGGTGTCAGT GTCTGTTTGG TCGATAAGAG AATGGAGAGA GCGGACGAGG CAACTCTTGC
TBRFV_Rv2     TGGTGTCAGT GTCTGTTTGG TCGATAAGAG AATGGAGAGA GCGGACGAGG CAACTCTTGC
TBRFV_Fw9     ---------- ---------- ---------- ---------- ---------- ----------
TBRFV_Rv1     ---------- ---------- ---------- ---------- ---------- ----------
```

```
              ....|....| ....|....| ....|....| ....|....| ....|....| ....|....|
                    5230       5240       5250       5260       5270       5280
TBRFV_PT1     TTCATACTAT ACCGCAGCGG CTAAGAAAAG GTTTCAGTTC AAAGTCGTTC CAAATTACAA
TBRFV_PT2     TTCATACTAT ACCGCAGCGG CTAAGAAAAG GTTTCAGTTC AAAGTCGTTC CAAATTACAA
TBRFV_Fw1     ---------- ---------- ---------- ---------- ---------- ----------
TBRFV_Rv9     ---------- ---------- ---------- ---------- ---------- ----------
TBRFV_Fw2     ---------- ---------- ---------- ---------- ---------- ----------
TBRFV_Rv8     ---------- ---------- ---------- ---------- ---------- ----------
TBRFV_Fw3     ---------- ---------- ---------- ---------- ---------- ----------
TBRFV_Rv7     ---------- ---------- ---------- ---------- ---------- ----------
TBRFV_Fw4     ---------- ---------- ---------- ---------- ---------- ----------
TBRFV_Rv6     ---------- ---------- ---------- ---------- ---------- ----------
TBRFV_Fw5     ---------- ---------- ---------- ---------- ---------- ----------
TBRFV_Rv5     ---------- ---------- ---------- ---------- ---------- ----------
TBRFV_Fw6     ---------- ---------- ---------- ---------- ---------- ----------
TBRFV_Rv4     ---------- ---------- ---------- ---------- ---------- ----------
TBRFV_Fw7     ---------- ---------- ---------- ---------- ---------- ----------
TBRFV_Rv3     ---------- ---------- ---------- ---------- ---------- ----------
TBRFV_Fw8     TTCATACTAT ACCGCAGCGG CTAAGAAAAG GTTTCAGTTC AAAGTCGTTC CAAATTACAA
TBRFV_Rv2     TTCATACTAT ACCGCAGCGG CTAAGAAAAG GTTTCAGTTC AAAGTCGTTC CAAATTACAA
TBRFV_Fw9     ---------- ---------- ---------- ---------- ---------- ----------
TBRFV_Rv1     ---------- ---------- ---------- ---------- ---------- ----------
```

```
             ....|....| ....|....| ....|....| ....|....| ....|....| ....|....|
                  5290       5300       5310       5320       5330       5340
TBRFV_PT1    CATCACTACC AAGGACGCAG AAAAGGCAGT TTGGCAAGTA CTAGTTAATA TTAGAAATGT
TBRFV_PT2    CATCACTACC AAGGACGCAG AAAAGGCAGT TTGGCAAGTA CTAGTTAATA TTAGAAATGT
TBRFV Fw1    ---------- ---------- ---------- ---------- ---------- ----------
TBRFV Rv9    ---------- ---------- ---------- ---------- ---------- ----------
TBRFV Fw2    ---------- ---------- ---------- ---------- ---------- ----------
TBRFV Rv8    ---------- ---------- ---------- ---------- ---------- ----------
TBRFV Fw3    ---------- ---------- ---------- ---------- ---------- ----------
TBRFV Rv7    ---------- ---------- ---------- ---------- ---------- ----------
TBRFV Fw4    ---------- ---------- ---------- ---------- ---------- ----------
TBRFV Rv6    ---------- ---------- ---------- ---------- ---------- ----------
TBRFV Fw5    ---------- ---------- ---------- ---------- ---------- ----------
TBRFV Rv5    ---------- ---------- ---------- ---------- ---------- ----------
TBRFV Fw6    ---------- ---------- ---------- ---------- ---------- ----------
TBRFV Rv4    ---------- ---------- ---------- ---------- ---------- ----------
TBRFV Fw7    ---------- ---------- ---------- ---------- ---------- ----------
TBRFV Rv3    ---------- ---------- ---------- ---------- ---------- ----------
TBRFV Fw8    CATCACTACC AAGGACGCAG AAAAGGCAGT TTGGCAAGTA CTAGTTAATA TTAGAAATGT
TBRFV Rv2    CATCACTACC AAGGACGCAG AAAAGGCAGT TTGGCAAGTA CTAGTTAATA TTAGAAATGT
TBRFV Fw9    ---------- ---------- ---------- ---------- ---------- ----------
TBRFV Rv1    ---------- ---------- ---------- ---------- ---------- ----------
```

```
             ....|....| ....|....| ....|....| ....|....| ....|....| ....|....|
                  5350       5360       5370       5380       5390       5400
TBRFV_PT1    TAAAATTGCT GCGGGTTACT GTCCGCTGTC ATTAGAATTT GTGTCAGTGT GTATTGTTTA
TBRFV_PT2    TAAAATTGCT GCGGGTTACT GTCCGCTGTC ATTAGAATTT GTGTCAGTGT GTATTGTTTA
TBRFV Fw1    ---------- ---------- ---------- ---------- ---------- ----------
TBRFV Rv9    ---------- ---------- ---------- ---------- ---------- ----------
TBRFV Fw2    ---------- ---------- ---------- ---------- ---------- ----------
TBRFV Rv8    ---------- ---------- ---------- ---------- ---------- ----------
TBRFV Fw3    ---------- ---------- ---------- ---------- ---------- ----------
TBRFV Rv7    ---------- ---------- ---------- ---------- ---------- ----------
TBRFV Fw4    ---------- ---------- ---------- ---------- ---------- ----------
TBRFV Rv6    ---------- ---------- ---------- ---------- ---------- ----------
TBRFV Fw5    ---------- ---------- ---------- ---------- ---------- ----------
TBRFV Rv5    ---------- ---------- ---------- ---------- ---------- ----------
TBRFV Fw6    ---------- ---------- ---------- ---------- ---------- ----------
TBRFV Rv4    ---------- ---------- ---------- ---------- ---------- ----------
TBRFV Fw7    ---------- ---------- ---------- ---------- ---------- ----------
TBRFV Rv3    ---------- ---------- ---------- ---------- ---------- ----------
TBRFV Fw8    TAAAATTGCT GCGGGTTACT GTCCGCTGTC ATTAGAATTT GTGTCAGTGT GTATTGTTTA
TBRFV Rv2    TAAAATTGCT GCGGGTTACT GTCCGCTGTC ATTAGAATTT GTGTCAGTGT GTATTGTTTA
TBRFV Fw9    ---------- ---------- ---------- ---------- ---------- ----------
TBRFV Rv1    ---------- ---------- ---------- ---------- ---------- ----------
```

```
                ....|....| ....|....| ....|....| ....|....| ....|....| ....|....|
                       5410       5420       5430       5440       5450       5460
TBRFV_PT1       TAAAAATATT ATAAAACTCG GTTTGAGAGA GAAAATTACG AGCGTCACGG ATGGAGGGCC
TBRFV_PT2       TAAAAATATT ATAAAACTCG GTTTGAGAGA GAAAATTACG AGCGTCACGG ATGGAGGGCC
TBRFV Fw1       ---------- ---------- ---------- ---------- ---------- ----------
TBRFV Rv9       ---------- ---------- ---------- ---------- ---------- ----------
TBRFV Fw2       ---------- ---------- ---------- ---------- ---------- ----------
TBRFV Rv8       ---------- ---------- ---------- ---------- ---------- ----------
TBRFV Fw3       ---------- ---------- ---------- ---------- ---------- ----------
TBRFV Rv7       ---------- ---------- ---------- ---------- ---------- ----------
TBRFV Fw4       ---------- ---------- ---------- ---------- ---------- ----------
TBRFV Rv6       ---------- ---------- ---------- ---------- ---------- ----------
TBRFV Fw5       ---------- ---------- ---------- ---------- ---------- ----------
TBRFV Rv5       ---------- ---------- ---------- ---------- ---------- ----------
TBRFV Fw6       ---------- ---------- ---------- ---------- ---------- ----------
TBRFV Rv4       ---------- ---------- ---------- ---------- ---------- ----------
TBRFV Fw7       ---------- ---------- ---------- ---------- ---------- ----------
TBRFV Rv3       ---------- ---------- ---------- ---------- ---------- ----------
TBRFV Fw8       TAAAAATATT ATAAAACTCG GTTTGAGAGA GAAAATTACG AGCGTCACGG ATGGAGGGCC
TBRFV Rv2       TAAAAATATT ATAAAACTCG GTTTGAGAGA GAAAATTACG AGCGTCACGG ATGGAGGGCC
TBRFV Fw9       ---------- ---------- ---------- ---------- ---------- ----------
TBRFV Rv1       ---------- ---------- ---------- ---------- ---------- ----------
```

```
                ....|....| ....|....| ....|....| ....|....| ....|....| ....|....|
                       5470       5480       5490       5500       5510       5520
TBRFV_PT1       CATGGAACTA TCAGAAGAAG TTGTTGATGA GTTCATGGAA GAAGTCCCGA TGTCTGTAAG
TBRFV_PT2       CATGGAACTA TCAGAAGAAG TTGTTGATGA GTTCATGGAA GAAGTCCCGA TGTCTGTAAG
TBRFV Fw1       ---------- ---------- ---------- ---------- ---------- ----------
TBRFV Rv9       ---------- ---------- ---------- ---------- ---------- ----------
TBRFV Fw2       ---------- ---------- ---------- ---------- ---------- ----------
TBRFV Rv8       ---------- ---------- ---------- ---------- ---------- ----------
TBRFV Fw3       ---------- ---------- ---------- ---------- ---------- ----------
TBRFV Rv7       ---------- ---------- ---------- ---------- ---------- ----------
TBRFV Fw4       ---------- ---------- ---------- ---------- ---------- ----------
TBRFV Rv6       ---------- ---------- ---------- ---------- ---------- ----------
TBRFV Fw5       ---------- ---------- ---------- ---------- ---------- ----------
TBRFV Rv5       ---------- ---------- ---------- ---------- ---------- ----------
TBRFV Fw6       ---------- ---------- ---------- ---------- ---------- ----------
TBRFV Rv4       ---------- ---------- ---------- ---------- ---------- ----------
TBRFV Fw7       ---------- ---------- ---------- ---------- ---------- ----------
TBRFV Rv3       ---------- ---------- ---------- ---------- ---------- ----------
TBRFV Fw8       CATGGAACTA TCAGAAGAAG TTGTTGATGA GTTCATGGAA GAAGTCCCGA TGTCTGTAAG
TBRFV Rv2       CATGGAACTA TCAGAAGAAG TTGTTGATGA GTTCATGGAA GAAGTCCCGA TGTCTGTAAG
TBRFV Fw9       ---------- ---------- ---------- ---------- ---------- ----------
TBRFV Rv1       ---------- ---------- ---------- ---------- ---------- --------AG
```

```
               ....|....| ....|....| ....|....| ....|....| ....|....| ....|....|
                      5530       5540       5550       5560       5570       5580
TBRFV_PT1      GCTTGCAAAA TTTCGTTCGA AGACCGGAAA AAAGTTTAGT AGTAAAAGTG AGAATAATAG
TBRFV_PT2      GCTTGCAAAA TTTCGTTCGA AGACCGGAAA AAAGTTTAGT AGTAAAAGTG AGAATAATAG
TBRFV Fw1      ---------- ---------- ---------- ---------- ---------- ----------
TBRFV Rv9      ---------- ---------- ---------- ---------- ---------- ----------
TBRFV Fw2      ---------- ---------- ---------- ---------- ---------- ----------
TBRFV Rv8      ---------- ---------- ---------- ---------- ---------- ----------
TBRFV Fw3      ---------- ---------- ---------- ---------- ---------- ----------
TBRFV Rv7      ---------- ---------- ---------- ---------- ---------- ----------
TBRFV Fw4      ---------- ---------- ---------- ---------- ---------- ----------
TBRFV Rv6      ---------- ---------- ---------- ---------- ---------- ----------
TBRFV Fw5      ---------- ---------- ---------- ---------- ---------- ----------
TBRFV Rv5      ---------- ---------- ---------- ---------- ---------- ----------
TBRFV Fw6      ---------- ---------- ---------- ---------- ---------- ----------
TBRFV Rv4      ---------- ---------- ---------- ---------- ---------- ----------
TBRFV Fw7      ---------- ---------- ---------- ---------- ---------- ----------
TBRFV Rv3      ---------- ---------- ---------- ---------- ---------- ----------
TBRFV Fw8      GCTTGCAAAA TTTCGTTCGA AGACCGGAAA AAAGTTTAGT AGTAAAAGTG AGAATAATAG
TBRFV Rv2      GCTTGCAAAA TTTCGTTCGA AGACCGGAAA AAAGTTTAGT AGTAAAAGTG AGAATAATA-
TBRFV Fw9      ---------- ---------- -------AAA AAAGTTTAGT AGTAAAAGTG AGAATAATAG
TBRFV Rv1      GCTTGCAAAA TTTCGTTCGA AGACCGGAAA AAAGTTTAGT AGTAAAAGTG AGAATAATAG

               ....|....| ....|....| ....|....| ....|....| ....|....| ....|....|
                      5590       5600       5610       5620       5630       5640
TBRFV_PT1      TGGTAATAAT AGGCCGAAAC CAAACAAAAA CCAAAGGAAG GAAAAGGGTT TAAAAGTTAG
TBRFV_PT2      TGGTAATAAT AGGCCGAAAC CAAACAAAAA CCAAAGGAAG GAAAAGGGTT TAAAAGTTAG
TBRFV Fw1      ---------- ---------- ---------- ---------- ---------- ----------
TBRFV Rv9      ---------- ---------- ---------- ---------- ---------- ----------
TBRFV Fw2      ---------- ---------- ---------- ---------- ---------- ----------
TBRFV Rv8      ---------- ---------- ---------- ---------- ---------- ----------
TBRFV Fw3      ---------- ---------- ---------- ---------- ---------- ----------
TBRFV Rv7      ---------- ---------- ---------- ---------- ---------- ----------
TBRFV Fw4      ---------- ---------- ---------- ---------- ---------- ----------
TBRFV Rv6      ---------- ---------- ---------- ---------- ---------- ----------
TBRFV Fw5      ---------- ---------- ---------- ---------- ---------- ----------
TBRFV Rv5      ---------- ---------- ---------- ---------- ---------- ----------
TBRFV Fw6      ---------- ---------- ---------- ---------- ---------- ----------
TBRFV Rv4      ---------- ---------- ---------- ---------- ---------- ----------
TBRFV Fw7      ---------- ---------- ---------- ---------- ---------- ----------
TBRFV Rv3      ---------- ---------- ---------- ---------- ---------- ----------
TBRFV Fw8      TGGTAATAAT AGGCCGAAAC CAAACAAAAA CCAAAGGAAG GAAAA-GG-T TA--------
TBRFV Rv2      ---------- ---------- ---------- ---------- ---------- ----------
TBRFV Fw9      TGGTAATAAT AGGCCGAAAC CAAACAAAAA CCAAAGGAAG GAAAAGGGTT TAAAAGTTAG
TBRFV Rv1      TGGTAATAAT AGGCCGAAAC CAAACAAAAA CCAAAGGAAG GAAAAGGGTT TAAAAGTTAG

               ....|....| ....|....| ....|....| ....|....| ....|....| ....|....|
                      5650       5660       5670       5680       5690       5700
TBRFV_PT1      GGTTGAGAAG GATAATTTAA TTGATAATGA ATTGGAGACT TACGTCGCCG ATTCAGATTC
TBRFV_PT2      GGTTGAGAAG GATAATTTAA TTGATAATGA ATTGGAGACT TACGTCGCCG ATTCAGATTC
TBRFV Fw1      ---------- ---------- ---------- ---------- ---------- ----------
TBRFV Rv9      ---------- ---------- ---------- ---------- ---------- ----------
TBRFV Fw2      ---------- ---------- ---------- ---------- ---------- ----------
TBRFV Rv8      ---------- ---------- ---------- ---------- ---------- ----------
TBRFV Fw3      ---------- ---------- ---------- ---------- ---------- ----------
TBRFV Rv7      ---------- ---------- ---------- ---------- ---------- ----------
TBRFV Fw4      ---------- ---------- ---------- ---------- ---------- ----------
```

```
TBRFV Rv6    ---------- ---------- ---------- ---------- ---------- ----------
TBRFV Fw5    ---------- ---------- ---------- ---------- ---------- ----------
TBRFV Rv5    ---------- ---------- ---------- ---------- ---------- ----------
TBRFV Fw6    ---------- ---------- ---------- ---------- ---------- ----------
TBRFV Rv4    ---------- ---------- ---------- ---------- ---------- ----------
TBRFV Fw7    ---------- ---------- ---------- ---------- ---------- ----------
TBRFV Rv3    ---------- ---------- ---------- ---------- ---------- ----------
TBRFV Fw8    ---------- ---------- ---------- ---------- ---------- ----------
TBRFV Rv2    ---------- ---------- ---------- ---------- ---------- ----------
TBRFV Fw9    GGTTGAGAAG GATAATTTAA TTGATAATGA ATTGGAGACT TACGTCGCCG ATTCAGATTC
TBRFV Rv1    GGTTGAGAAG GATAATTTAA TTGATAATGA ATTGGAGACT TACGTCGCCG ATTCAGATTC

             ....|....| ....|....| ....|....| ....|....| ....|....| ....|....|
                   5710       5720       5730       5740       5750       5760
TBRFV_PT1    GTATTAAATA TGTCTTACAC AATCGCAACT CCATCGCAAT TTGTGTTTTT GTCATCAGCA
TBRFV_PT2    GTATTAAATA TGTCTTACAC AATCGCAACT CCATCGCAAT TTGTGTTTTT GTCATCAGCA
TBRFV Fw1    ---------- ---------- ---------- ---------- ---------- ----------
TBRFV Rv9    ---------- ---------- ---------- ---------- ---------- ----------
TBRFV Fw2    ---------- ---------- ---------- ---------- ---------- ----------
TBRFV Rv8    ---------- ---------- ---------- ---------- ---------- ----------
TBRFV Fw3    ---------- ---------- ---------- ---------- ---------- ----------
TBRFV Rv7    ---------- ---------- ---------- ---------- ---------- ----------
TBRFV Fw4    ---------- ---------- ---------- ---------- ---------- ----------
TBRFV Rv6    ---------- ---------- ---------- ---------- ---------- ----------
TBRFV Fw5    ---------- ---------- ---------- ---------- ---------- ----------
TBRFV Rv5    ---------- ---------- ---------- ---------- ---------- ----------
TBRFV Fw6    ---------- ---------- ---------- ---------- ---------- ----------
TBRFV Rv4    ---------- ---------- ---------- ---------- ---------- ----------
TBRFV Fw7    ---------- ---------- ---------- ---------- ---------- ----------
TBRFV Rv3    ---------- ---------- ---------- ---------- ---------- ----------
TBRFV Fw8    ---------- ---------- ---------- ---------- ---------- ----------
TBRFV Rv2    ---------- ---------- ---------- ---------- ---------- ----------
TBRFV Fw9    GTATTAAATA TGTCTTACAC AATCGCAACT CCATCGCAAT TTGTGTTTTT GTCATCAGCA
TBRFV Rv1    GTATTAAATA TGTCTTACAC AATCGCAACT CCATCGCAAT TTGTGTTTTT GTCATCAGCA
```

https://doi.org/10.3390/plants15081240

```
             ....|....| ....|....| ....|....| ....|....| ....|....| ....|....|
                   5770       5780       5790       5800       5810       5820
TBRFV_PT1    TGGGCCGACC CTATAGAATT AATAAATTTA TGTACTAATT CACTAGGTAA TCAGTTCCAA
TBRFV_PT2    TGGGCCGACC CTATAGAATT AATAAATTTA TGTACTAATT CACTAGGTAA TCAGTTCCAA
TBRFV Fw1    ---------- ---------- ---------- ---------- ---------- ----------
TBRFV Rv9    ---------- ---------- ---------- ---------- ---------- ----------
TBRFV Fw2    ---------- ---------- ---------- ---------- ---------- ----------
TBRFV Rv8    ---------- ---------- ---------- ---------- ---------- ----------
TBRFV Fw3    ---------- ---------- ---------- ---------- ---------- ----------
TBRFV Rv7    ---------- ---------- ---------- ---------- ---------- ----------
TBRFV Fw4    ---------- ---------- ---------- ---------- ---------- ----------
TBRFV Rv6    ---------- ---------- ---------- ---------- ---------- ----------
TBRFV Fw5    ---------- ---------- ---------- ---------- ---------- ----------
TBRFV Rv5    ---------- ---------- ---------- ---------- ---------- ----------
TBRFV Fw6    ---------- ---------- ---------- ---------- ---------- ----------
TBRFV Rv4    ---------- ---------- ---------- ---------- ---------- ----------
TBRFV Fw7    ---------- ---------- ---------- ---------- ---------- ----------
TBRFV Rv3    ---------- ---------- ---------- ---------- ---------- ----------
TBRFV Fw8    ---------- ---------- ---------- ---------- ---------- ----------
TBRFV Rv2    ---------- ---------- ---------- ---------- ---------- ----------
TBRFV Fw9    TGGGCCGACC CTATAGAATT AATAAATTTA TGTACTAATT CACTAGGTAA TCAGTTCCAA
TBRFV Rv1    TGGGCCGACC CTATAGAATT AATAAATTTA TGTACTAATT CACTAGGTAA TCAGTTCCAA
```

```
             ....|....| ....|....| ....|....| ....|....| ....|....| ....|....|
                   5830       5840       5850       5860       5870       5880
TBRFV_PT1    ACACAACAAG CTAGAACAAC CGTTCAACGG CAATTTAGCG AAGTGTGGAA ACCTGTCCCT
TBRFV_PT2    ACACAACAAG CTAGAACAAC CGTTCAACGG CAATTTAGCG AAGTGTGGAA ACCTGTCCCT
TBRFV Fw1    ---------- ---------- ---------- ---------- ---------- ----------
TBRFV Rv9    ---------- ---------- ---------- ---------- ---------- ----------
TBRFV Fw2    ---------- ---------- ---------- ---------- ---------- ----------
TBRFV Rv8    ---------- ---------- ---------- ---------- ---------- ----------
TBRFV Fw3    ---------- ---------- ---------- ---------- ---------- ----------
TBRFV Rv7    ---------- ---------- ---------- ---------- ---------- ----------
TBRFV Fw4    ---------- ---------- ---------- ---------- ---------- ----------
TBRFV Rv6    ---------- ---------- ---------- ---------- ---------- ----------
TBRFV Fw5    ---------- ---------- ---------- ---------- ---------- ----------
TBRFV Rv5    ---------- ---------- ---------- ---------- ---------- ----------
TBRFV Fw6    ---------- ---------- ---------- ---------- ---------- ----------
TBRFV Rv4    ---------- ---------- ---------- ---------- ---------- ----------
TBRFV Fw7    ---------- ---------- ---------- ---------- ---------- ----------
TBRFV Rv3    ---------- ---------- ---------- ---------- ---------- ----------
TBRFV Fw8    ---------- ---------- ---------- ---------- ---------- ----------
TBRFV Rv2    ---------- ---------- ---------- ---------- ---------- ----------
TBRFV Fw9    ACACAACAAG CTAGAACAAC CGTTCAACGG CAATTTAGCG AAGTGTGGAA ACCTGTCCCT
TBRFV Rv1    ACACAACAAG CTAGAACAAC CGTTCAACGG CAATTTAGCG AAGTGTGGAA ACCTGTCCCT
```

```
              ....|....| ....|....| ....|....| ....|....| ....|....| ....|....|
                      5890       5900       5910       5920       5930       5940
TBRFV_PT1     CAAGTCACTG TTAGGTTTCC TGACAGTGGT TTTAAGGTGT ATAGGTACAA TGCGGTACTA
TBRFV_PT2     CAAGTCACTG TTAGGTTTCC TGACAGTGGT TTTAAGGTGT ATAGGTACAA TGCGGTACTA
TBRFV Fw1     ---------- ---------- ---------- ---------- ---------- ----------
TBRFV Rv9     ---------- ---------- ---------- ---------- ---------- ----------
TBRFV Fw2     ---------- ---------- ---------- ---------- ---------- ----------
TBRFV Rv8     ---------- ---------- ---------- ---------- ---------- ----------
TBRFV Fw3     ---------- ---------- ---------- ---------- ---------- ----------
TBRFV Rv7     ---------- ---------- ---------- ---------- ---------- ----------
TBRFV Fw4     ---------- ---------- ---------- ---------- ---------- ----------
TBRFV Rv6     ---------- ---------- ---------- ---------- ---------- ----------
TBRFV Fw5     ---------- ---------- ---------- ---------- ---------- ----------
TBRFV Rv5     ---------- ---------- ---------- ---------- ---------- ----------
TBRFV Fw6     ---------- ---------- ---------- ---------- ---------- ----------
TBRFV Rv4     ---------- ---------- ---------- ---------- ---------- ----------
TBRFV Fw7     ---------- ---------- ---------- ---------- ---------- ----------
TBRFV Rv3     ---------- ---------- ---------- ---------- ---------- ----------
TBRFV Fw8     ---------- ---------- ---------- ---------- ---------- ----------
TBRFV Rv2     ---------- ---------- ---------- ---------- ---------- ----------
TBRFV Fw9     CAAGTCACTG TTAGGTTTCC TGACAGTGGT TTTAAGGTGT ATAGGTACAA TGCGGTACTA
TBRFV Rv1     CAAGTCACTG TTAGGTTTCC TGACAGTGGT TTTAAGGTGT ATAGGTACAA TGCGGTACTA
```

```
              ....|....| ....|....| ....|....| ....|....| ....|....| ....|....|
                      5950       5960       5970       5980       5990       6000
TBRFV_PT1     GATCCTCTAG TTACTGCTTT GTTAGGAGCT TTCGATACTA GAAATAGGAT TATAGAAGTC
TBRFV_PT2     GATCCTCTAG TTACTGCTTT GTTAGGAGCT TTCGATACTA GAAATAGGAT TATAGAAGTC
TBRFV Fw1     ---------- ---------- ---------- ---------- ---------- ----------
TBRFV Rv9     ---------- ---------- ---------- ---------- ---------- ----------
TBRFV Fw2     ---------- ---------- ---------- ---------- ---------- ----------
TBRFV Rv8     ---------- ---------- ---------- ---------- ---------- ----------
TBRFV Fw3     ---------- ---------- ---------- ---------- ---------- ----------
TBRFV Rv7     ---------- ---------- ---------- ---------- ---------- ----------
TBRFV Fw4     ---------- ---------- ---------- ---------- ---------- ----------
TBRFV Rv6     ---------- ---------- ---------- ---------- ---------- ----------
TBRFV Fw5     ---------- ---------- ---------- ---------- ---------- ----------
TBRFV Rv5     ---------- ---------- ---------- ---------- ---------- ----------
TBRFV Fw6     ---------- ---------- ---------- ---------- ---------- ----------
TBRFV Rv4     ---------- ---------- ---------- ---------- ---------- ----------
TBRFV Fw7     ---------- ---------- ---------- ---------- ---------- ----------
TBRFV Rv3     ---------- ---------- ---------- ---------- ---------- ----------
TBRFV Fw8     ---------- ---------- ---------- ---------- ---------- ----------
TBRFV Rv2     ---------- ---------- ---------- ---------- ---------- ----------
TBRFV Fw9     GATCCTCTAG TTACTGCTTT GTTAGGAGCT TTCGATACTA GAAATAGGAT TATAGAAGTC
TBRFV Rv1     GATCCTCTAG TTACTGCTTT GTTAGGAGCT TTCGATACTA GAAATAGGAT TATAGAAGTC
```

```
              ....|....| ....|....| ....|....| ....|....| ....|....| ....|....|
                      6010       6020       6030       6040       6050       6060
TBRFV_PT1     GAAAATCAGG CGAACCCGAC AACCGCCGAA ACGTTAGACG CTACTCGTAG AGTAGATGAC
TBRFV_PT2     GAAAATCAGG CGAACCCGAC AACCGCCGAA ACGTTAGACG CTACTCGTAG AGTAGATGAC
TBRFV Fw1     ---------- ---------- ---------- ---------- ---------- ----------
TBRFV Rv9     ---------- ---------- ---------- ---------- ---------- ----------
TBRFV Fw2     ---------- ---------- ---------- ---------- ---------- ----------
TBRFV Rv8     ---------- ---------- ---------- ---------- ---------- ----------
TBRFV Fw3     ---------- ---------- ---------- ---------- ---------- ----------
```

```
TBRFV Rv7      ---------- ---------- ---------- ---------- ---------- ----------
TBRFV Fw4      ---------- ---------- ---------- ---------- ---------- ----------
TBRFV Rv6      ---------- ---------- ---------- ---------- ---------- ----------
TBRFV Fw5      ---------- ---------- ---------- ---------- ---------- ----------
TBRFV Rv5      ---------- ---------- ---------- ---------- ---------- ----------
TBRFV Fw6      ---------- ---------- ---------- ---------- ---------- ----------
TBRFV Rv4      ---------- ---------- ---------- ---------- ---------- ----------
TBRFV Fw7      ---------- ---------- ---------- ---------- ---------- ----------
TBRFV Rv3      ---------- ---------- ---------- ---------- ---------- ----------
TBRFV Fw8      ---------- ---------- ---------- ---------- ---------- ----------
TBRFV Rv2      ---------- ---------- ---------- ---------- ---------- ----------
TBRFV Fw9      GAAAATCAGG CGAACCCGAC AACCGCCGAA ACGTTAGACG CTACTCGTAG AGTAGATGAC
TBRFV Rv1      GAAAATCAGG CGAACCCGAC AACCGCCGAA ACGTTAGACG CTACTCGTAG AGTAGATGAC

               ....|....| ....|....| ....|....| ....|....| ....|....| ....|....|
                     6070       6080       6090       6100       6110       6120
TBRFV_PT1      GCAACGGTGG CTATAAGGAG CGCTATAAAT AATTTAGTAG TAGAATTGGT CAAAGGAACA
TBRFV_PT2      GCAACGGTGG CTATAAGGAG CGCTATAAAT AATTTAGTAG TAGAATTGGT CAAAGGAACA
TBRFV Fw1      ---------- ---------- ---------- ---------- ---------- ----------
TBRFV Rv9      ---------- ---------- ---------- ---------- ---------- ----------
TBRFV Fw2      ---------- ---------- ---------- ---------- ---------- ----------
TBRFV Rv8      ---------- ---------- ---------- ---------- ---------- ----------
TBRFV Fw3      ---------- ---------- ---------- ---------- ---------- ----------
TBRFV Rv7      ---------- ---------- ---------- ---------- ---------- ----------
TBRFV Fw4      ---------- ---------- ---------- ---------- ---------- ----------
TBRFV Rv6      ---------- ---------- ---------- ---------- ---------- ----------
TBRFV Fw5      ---------- ---------- ---------- ---------- ---------- ----------
TBRFV Rv5      ---------- ---------- ---------- ---------- ---------- ----------
TBRFV Fw6      ---------- ---------- ---------- ---------- ---------- ----------
TBRFV Rv4      ---------- ---------- ---------- ---------- ---------- ----------
TBRFV Fw7      ---------- ---------- ---------- ---------- ---------- ----------
TBRFV Rv3      ---------- ---------- ---------- ---------- ---------- ----------
TBRFV Fw8      ---------- ---------- ---------- ---------- ---------- ----------
TBRFV Rv2      ---------- ---------- ---------- ---------- ---------- ----------
TBRFV Fw9      GCAACGGTGG CTATAAGGAG CGCTATAAAT AATTTAGTAG TAGAATTGGT CAAAGGAACA
TBRFV Rv1      GCAACGGTGG CTATAAGGAG CGCTATAAAT AATTTAGTAG TAGAATTGGT CAAAGGAACA
```

```
               ....|....| ....|....| ....|....| ....|....| ....|....| ....|....|
                       6130       6140       6150       6160       6170       6180
TBRFV_PT1      GGTTTGTACA ATCAGAGCAC ATTTGAAAGT GCATCCGGTT TACAATGGTC CTCTGCACCT
TBRFV_PT2      GGTTTGTACA ATCAGAGCAC ATTTGAAAGT GCATCCGGTT TACAATGGTC CTCTGCACCT
TBRFV Fw1      ---------- ---------- ---------- ---------- ---------- ----------
TBRFV Rv9      ---------- ---------- ---------- ---------- ---------- ----------
TBRFV Fw2      ---------- ---------- ---------- ---------- ---------- ----------
TBRFV Rv8      ---------- ---------- ---------- ---------- ---------- ----------
TBRFV Fw3      ---------- ---------- ---------- ---------- ---------- ----------
TBRFV Rv7      ---------- ---------- ---------- ---------- ---------- ----------
TBRFV Fw4      ---------- ---------- ---------- ---------- ---------- ----------
TBRFV Rv6      ---------- ---------- ---------- ---------- ---------- ----------
TBRFV Fw5      ---------- ---------- ---------- ---------- ---------- ----------
TBRFV Rv5      ---------- ---------- ---------- ---------- ---------- ----------
TBRFV Fw6      ---------- ---------- ---------- ---------- ---------- ----------
TBRFV Rv4      ---------- ---------- ---------- ---------- ---------- ----------
TBRFV Fw7      ---------- ---------- ---------- ---------- ---------- ----------
TBRFV Rv3      ---------- ---------- ---------- ---------- ---------- ----------
TBRFV Fw8      ---------- ---------- ---------- ---------- ---------- ----------
TBRFV Rv2      ---------- ---------- ---------- ---------- ---------- ----------
TBRFV Fw9      GGTTTGTACA ATCAGAGCAC ATTTGAAAGT GCATCCGGTT TACAATGGTC CTCTGCACCT
TBRFV Rv1      GGTTTGTACA ATCAGAGCAC ATTTGAAAGT GCATCCGGTT TACAATGGTC CTCTGCACCT
```

```
               ....|....| ....|....| ....|....| ....|....| ....|....| ....|....|
                       6190       6200       6210       6220       6230       6240
TBRFV_PT1      GCATCTTGAG ATAATCGAGA TGCTTAAATA ACAGATTGTG TCTGCAAACA CACGTGGTAC
TBRFV_PT2      GCATCTTGAG ATAATCGAGA TGCTTAAATA ACAGATTGTG TCTGCAAACA CACGTGGTAC
TBRFV Fw1      ---------- ---------- ---------- ---------- ---------- ----------
TBRFV Rv9      ---------- ---------- ---------- ---------- ---------- ----------
TBRFV Fw2      ---------- ---------- ---------- ---------- ---------- ----------
TBRFV Rv8      ---------- ---------- ---------- ---------- ---------- ----------
TBRFV Fw3      ---------- ---------- ---------- ---------- ---------- ----------
TBRFV Rv7      ---------- ---------- ---------- ---------- ---------- ----------
TBRFV Fw4      ---------- ---------- ---------- ---------- ---------- ----------
TBRFV Rv6      ---------- ---------- ---------- ---------- ---------- ----------
TBRFV Fw5      ---------- ---------- ---------- ---------- ---------- ----------
TBRFV Rv5      ---------- ---------- ---------- ---------- ---------- ----------
TBRFV Fw6      ---------- ---------- ---------- ---------- ---------- ----------
TBRFV Rv4      ---------- ---------- ---------- ---------- ---------- ----------
TBRFV Fw7      ---------- ---------- ---------- ---------- ---------- ----------
TBRFV Rv3      ---------- ---------- ---------- ---------- ---------- ----------
TBRFV Fw8      ---------- ---------- ---------- ---------- ---------- ----------
TBRFV Rv2      ---------- ---------- ---------- ---------- ---------- ----------
TBRFV Fw9      GCATCTTGAG ATAATCGAGA TGCTTAAATA ACAGATTGTG TCTGCAAACA CACGTGGTAC
TBRFV Rv1      GCATCTTGAG ATAATCGAGA TGCTTAAATA ACAGATTGTG TCTGCAAACA CACGTGGTAC
```

```
                 ....|....| ....|....| ....|....| ....|....| ....|....| ....|....|
                        6250       6260       6270       6280       6290       6300
TBRFV_PT1        GTACGATAAC GTATAGTGTT TTTCCCTCCA CTTAAATCGA AGGGTAGTGT CTTGGAGCGC
TBRFV_PT2        GTACGATAAC GTATAGTGTT TTTCCCTCCA CTTAAATCGA AGGGTAGTGT CTTGGAGCGC
TBRFV_Fw1        ---------- ---------- ---------- ---------- ---------- ----------
TBRFV Rv9        ---------- ---------- ---------- ---------- ---------- ----------
TBRFV Fw2        ---------- ---------- ---------- ---------- ---------- ----------
TBRFV Rv8        ---------- ---------- ---------- ---------- ---------- ----------
TBRFV Fw3        ---------- ---------- ---------- ---------- ---------- ----------
TBRFV Rv7        ---------- ---------- ---------- ---------- ---------- ----------
TBRFV Fw4        ---------- ---------- ---------- ---------- ---------- ----------
TBRFV Rv6        ---------- ---------- ---------- ---------- ---------- ----------
TBRFV Fw5        ---------- ---------- ---------- ---------- ---------- ----------
TBRFV Rv5        ---------- ---------- ---------- ---------- ---------- ----------
TBRFV Fw6        ---------- ---------- ---------- ---------- ---------- ----------
TBRFV Rv4        ---------- ---------- ---------- ---------- ---------- ----------
TBRFV Fw7        ---------- ---------- ---------- ---------- ---------- ----------
TBRFV Rv3        ---------- ---------- ---------- ---------- ---------- ----------
TBRFV Fw8        ---------- ---------- ---------- ---------- ---------- ----------
TBRFV Rv2        ---------- ---------- ---------- ---------- ---------- ----------
TBRFV Fw9        GTACGATAAC GTATAGTGTT TTTCCCTCCA CTTAAATCGA AGGGTAGTGT CTTGGAGCGC
TBRFV Rv1        GTACGATAAC GTATAGTGTT TTTCCCTCCA CTTAAATCGA AGGGTAGTGT CTTGGAGCGC

                 ....|....| ....|....| ....|....| ....|....| ....|....| ....|....|
                        6310       6320       6330       6340       6350       6360
TBRFV_PT1        GCGGGACAAA TGTGTATGGT TCATACACAT CCGTAGGCAC GTAATAAAGC GAGGGATTCG
TBRFV_PT2        GCGGGACAAA TGTGTATGGT TCATACACAT CCGTAGGCAC GTAATAAAGC GAGGGATTCG
TBRFV_Fw1        ---------- ---------- ---------- ---------- ---------- ----------
TBRFV Rv9        ---------- ---------- ---------- ---------- ---------- ----------
TBRFV Fw2        ---------- ---------- ---------- ---------- ---------- ----------
TBRFV Rv8        ---------- ---------- ---------- ---------- ---------- ----------
TBRFV Fw3        ---------- ---------- ---------- ---------- ---------- ----------
TBRFV Rv7        ---------- ---------- ---------- ---------- ---------- ----------
TBRFV Fw4        ---------- ---------- ---------- ---------- ---------- ----------
TBRFV Rv6        ---------- ---------- ---------- ---------- ---------- ----------
TBRFV Fw5        ---------- ---------- ---------- ---------- ---------- ----------
TBRFV Rv5        ---------- ---------- ---------- ---------- ---------- ----------
TBRFV Fw6        ---------- ---------- ---------- ---------- ---------- ----------
TBRFV Rv4        ---------- ---------- ---------- ---------- ---------- ----------
TBRFV Fw7        ---------- ---------- ---------- ---------- ---------- ----------
TBRFV Rv3        ---------- ---------- ---------- ---------- ---------- ----------
TBRFV Fw8        ---------- ---------- ---------- ---------- ---------- ----------
TBRFV Rv2        ---------- ---------- ---------- ---------- ---------- ----------
TBRFV Fw9        GCGGGACAAA TGTGTATGGT TCATACACAT CCGTAGGCAC GTAATAAAGC GAGGGATTCG
TBRFV Rv1        GCGGGACAAA TGTGTATGGT TCATACACAT CCG------- ---------- ----------
```

```
          ....|....| ....|....| ....|....
                   6370       6380
TBRFV_PT1 AATTCCCCCG GAACCCCCGG TAGGGGCCC
TBRFV_PT2 AATTCCCCC- ---------- ---------
TBRFV Fw1 ---------- ---------- ---------
TBRFV Rv9 ---------- ---------- ---------
TBRFV Fw2 ---------- ---------- ---------
TBRFV Rv8 ---------- ---------- ---------
TBRFV Fw3 ---------- ---------- ---------
TBRFV Rv7 ---------- ---------- ---------
TBRFV Fw4 ---------- ---------- ---------
TBRFV Rv6 ---------- ---------- ---------
TBRFV Fw5 ---------- ---------- ---------
TBRFV Rv5 ---------- ---------- ---------
TBRFV Fw6 ---------- ---------- ---------
TBRFV Rv4 ---------- ---------- ---------
TBRFV Fw7 ---------- ---------- ---------
TBRFV Rv3 ---------- ---------- ---------
TBRFV Fw8 ---------- ---------- ---------
TBRFV Rv2 ---------- ---------- ---------
TBRFV Fw9 AATTCCCC-- ---------- ---------
TBRFV Rv1 ---------- ---------- ---------
```

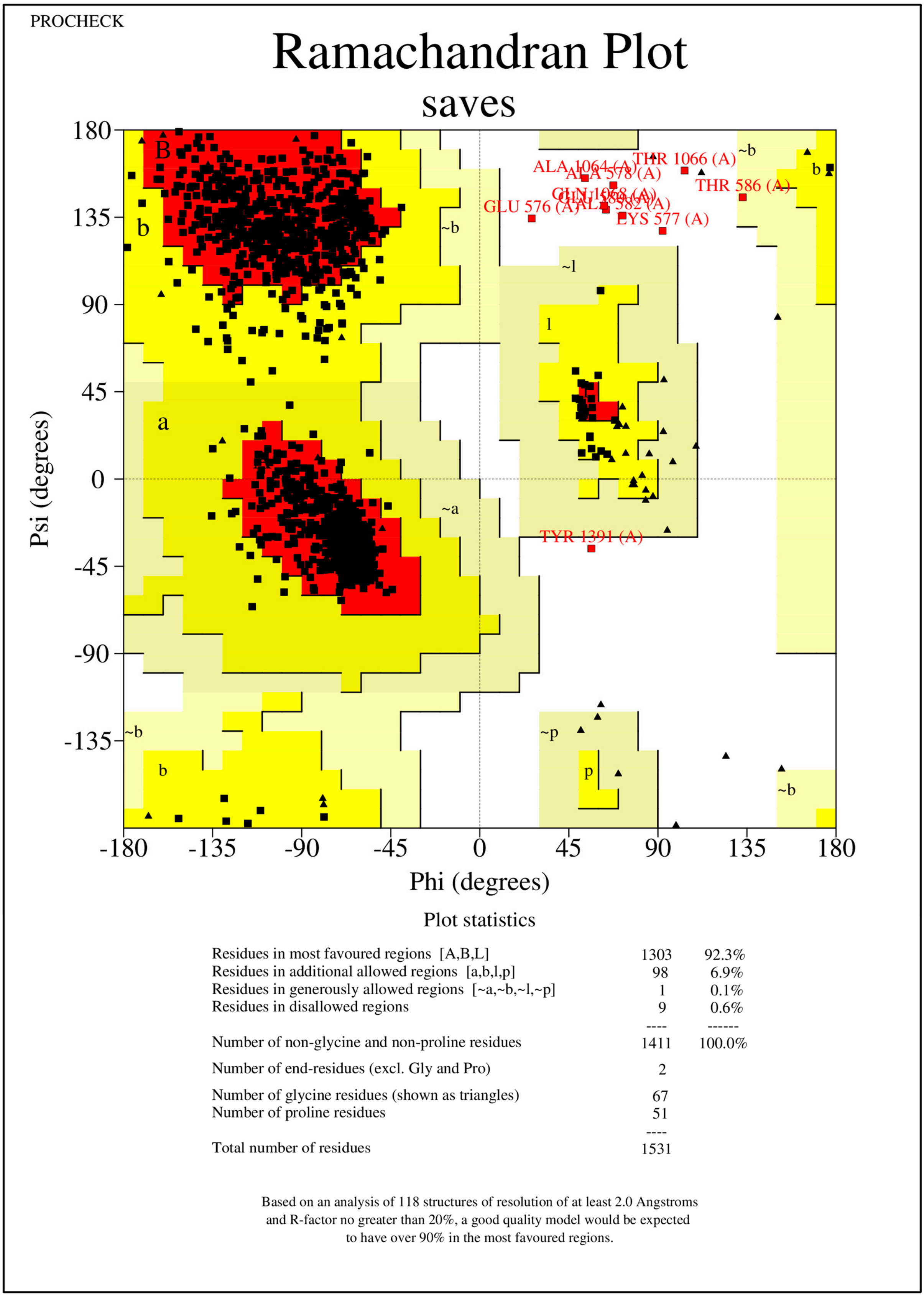

saves_01.ps

Figure S1. Ramachandran plot analysis (PROCHECK) for the 183 kDa replicase of the reference sequence (NC_028478.1 corresponding to Tom1-Jo).

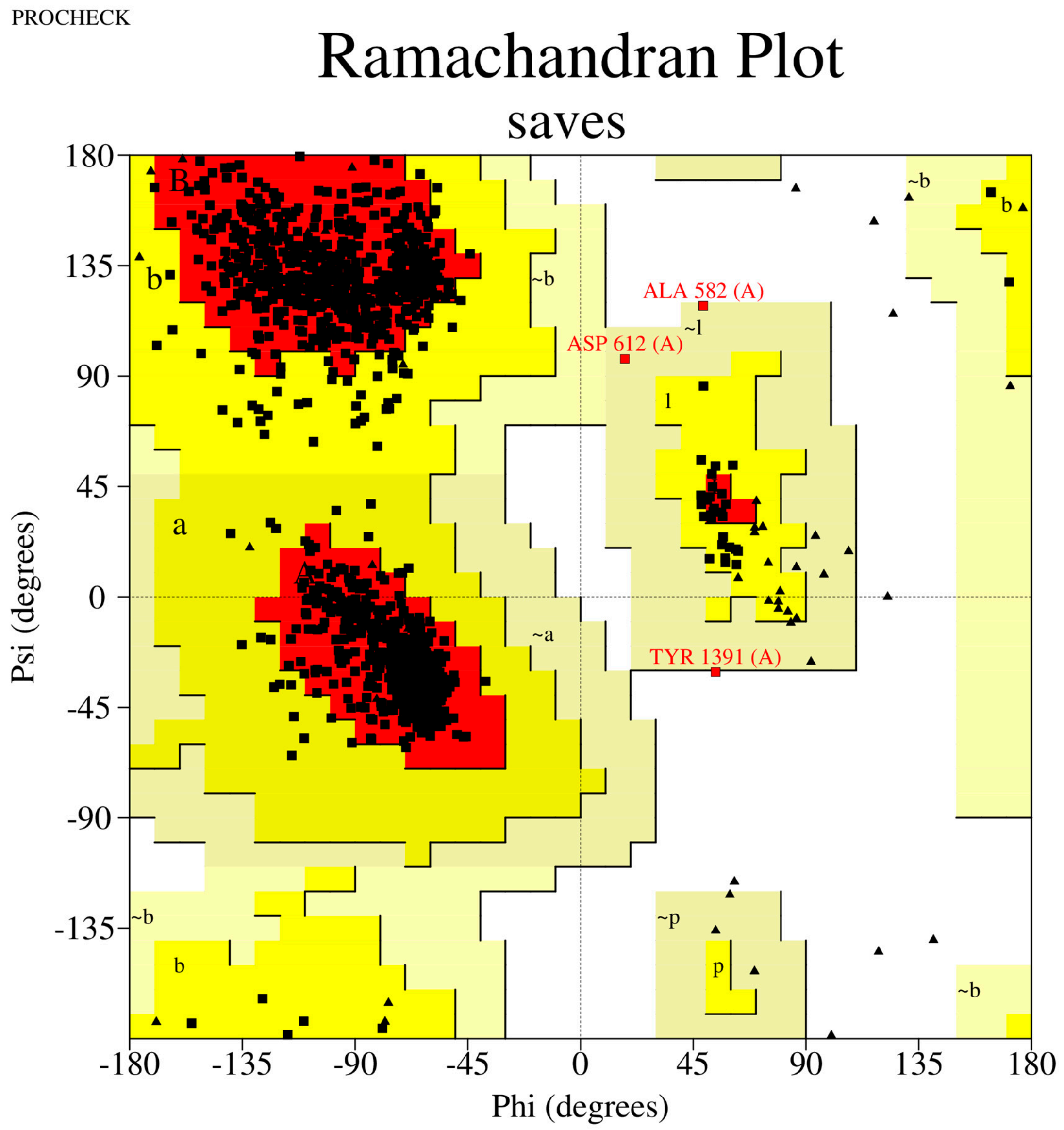

Plot statistics

Residues in most favoured regions [A,B,L]	1319	93.5%
Residues in additional allowed regions [a,b,l,p]	89	6.3%
Residues in generously allowed regions [~a,~b,~l,~p]	2	0.1%
Residues in disallowed regions	1	0.1%
	----	------
Number of non-glycine and non-proline residues	1411	100.0%
Number of end-residues (excl. Gly and Pro)	2	
Number of glycine residues (shown as triangles)	67	
Number of proline residues	51	

Total number of residues	1531	

Based on an analysis of 118 structures of resolution of at least 2.0 Angstroms and R-factor no greater than 20%, a good quality model would be expected to have over 90% in the most favoured regions.

saves_01.ps

Figure S2. Ramachandran plot analysis (PROCHECK) for the 183 kDa replicase of the ToBRFV_PT1 isolate (PV978367.1).

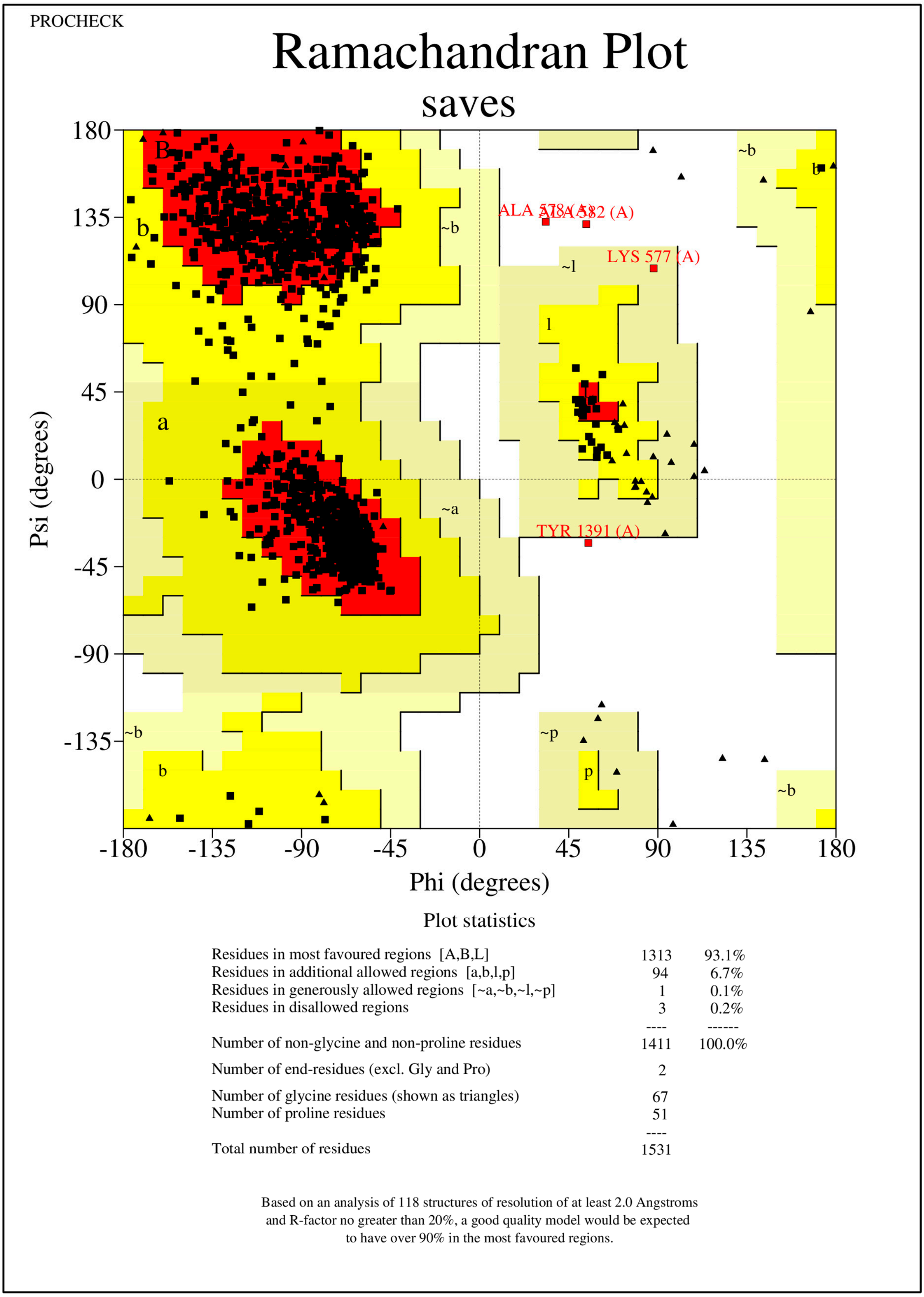

saves_01.ps

Figure S3. Ramachandran plot analysis (PROCHECK) for the 183 kDa replicase of the ToBRFV_PT2 isolate (PV978368.1).

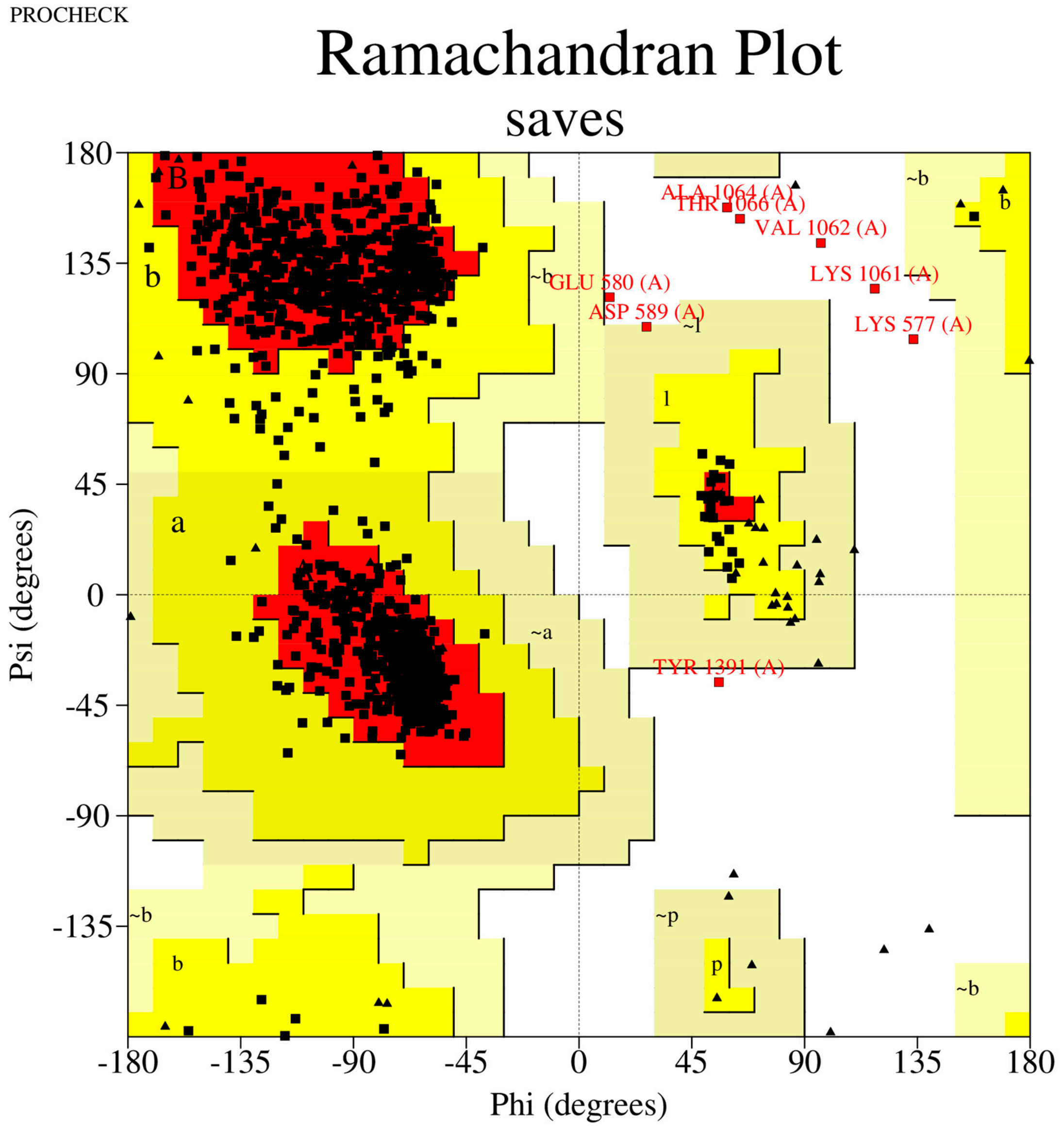

Plot statistics

Residues in most favoured regions [A,B,L]	1313	93.1%
Residues in additional allowed regions [a,b,l,p]	90	6.4%
Residues in generously allowed regions [~a,~b,~l,~p]	1	0.1%
Residues in disallowed regions	7	0.5%
	----	------
Number of non-glycine and non-proline residues	1411	100.0%
Number of end-residues (excl. Gly and Pro)	2	
Number of glycine residues (shown as triangles)	67	
Number of proline residues	51	

Total number of residues	1531	

Based on an analysis of 118 structures of resolution of at least 2.0 Angstroms
and R-factor no greater than 20%, a good quality model would be expected
to have over 90% in the most favoured regions.

saves_01.ps

Figure S4. Ramachandran plot analysis (PROCHECK) for the 183 kDa replicase of the Israeli isolate (OM_515237.1).

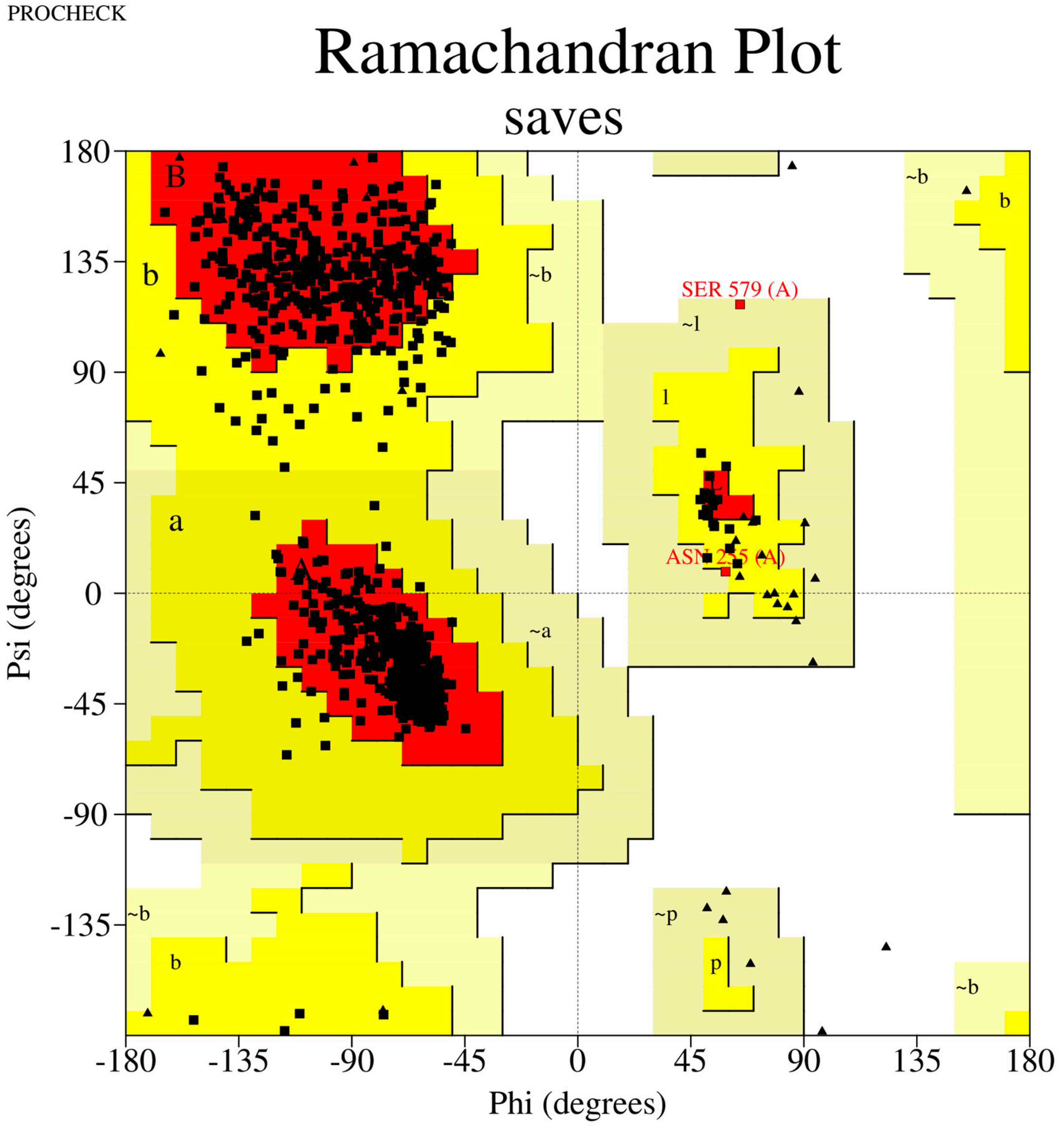

Plot statistics

Residues in most favoured regions [A,B,L]	887	92.6%
Residues in additional allowed regions [a,b,l,p]	69	7.2%
Residues in generously allowed regions [~a,~b,~l,~p]	2	0.2%
Residues in disallowed regions	0	0.0%
	----	------
Number of non-glycine and non-proline residues	958	100.0%
Number of end-residues (excl. Gly and Pro)	2	
Number of glycine residues (shown as triangles)	45	
Number of proline residues	31	

Total number of residues	1036	

Based on an analysis of 118 structures of resolution of at least 2.0 Angstroms
and R-factor no greater than 20%, a good quality model would be expected
to have over 90% in the most favoured regions.

saves_01.ps

Figure S5. Ramachandran plot analysis (PROCHECK) for the 126 kDa replicase of the reference sequence (NC_028478.1 corresponding to Tom1-Jo).

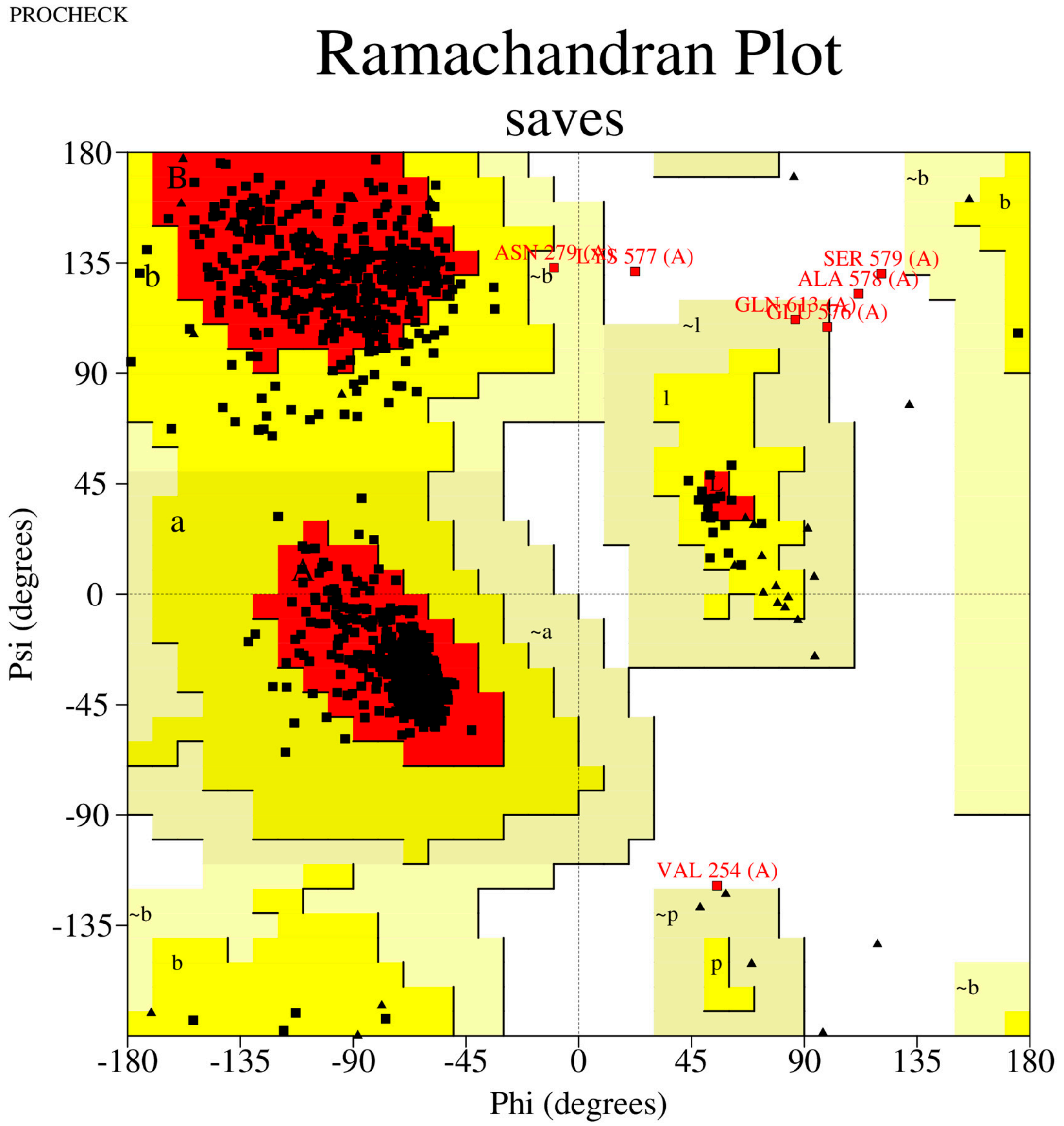

Plot statistics

Residues in most favoured regions [A,B,L]	880	91.9%
Residues in additional allowed regions [a,b,l,p]	71	7.4%
Residues in generously allowed regions [~a,~b,~l,~p]	3	0.3%
Residues in disallowed regions	4	0.4%
	----	------
Number of non-glycine and non-proline residues	958	100.0%
Number of end-residues (excl. Gly and Pro)	2	
Number of glycine residues (shown as triangles)	45	
Number of proline residues	31	

Total number of residues	1036	

Based on an analysis of 118 structures of resolution of at least 2.0 Angstroms and R-factor no greater than 20%, a good quality model would be expected to have over 90% in the most favoured regions.

saves_01.ps

Figure S6. Ramachandran plot analysis (PROCHECK) for the 126 kDa replicase of the ToBRFV_PT1 isolate (PV978367.1).

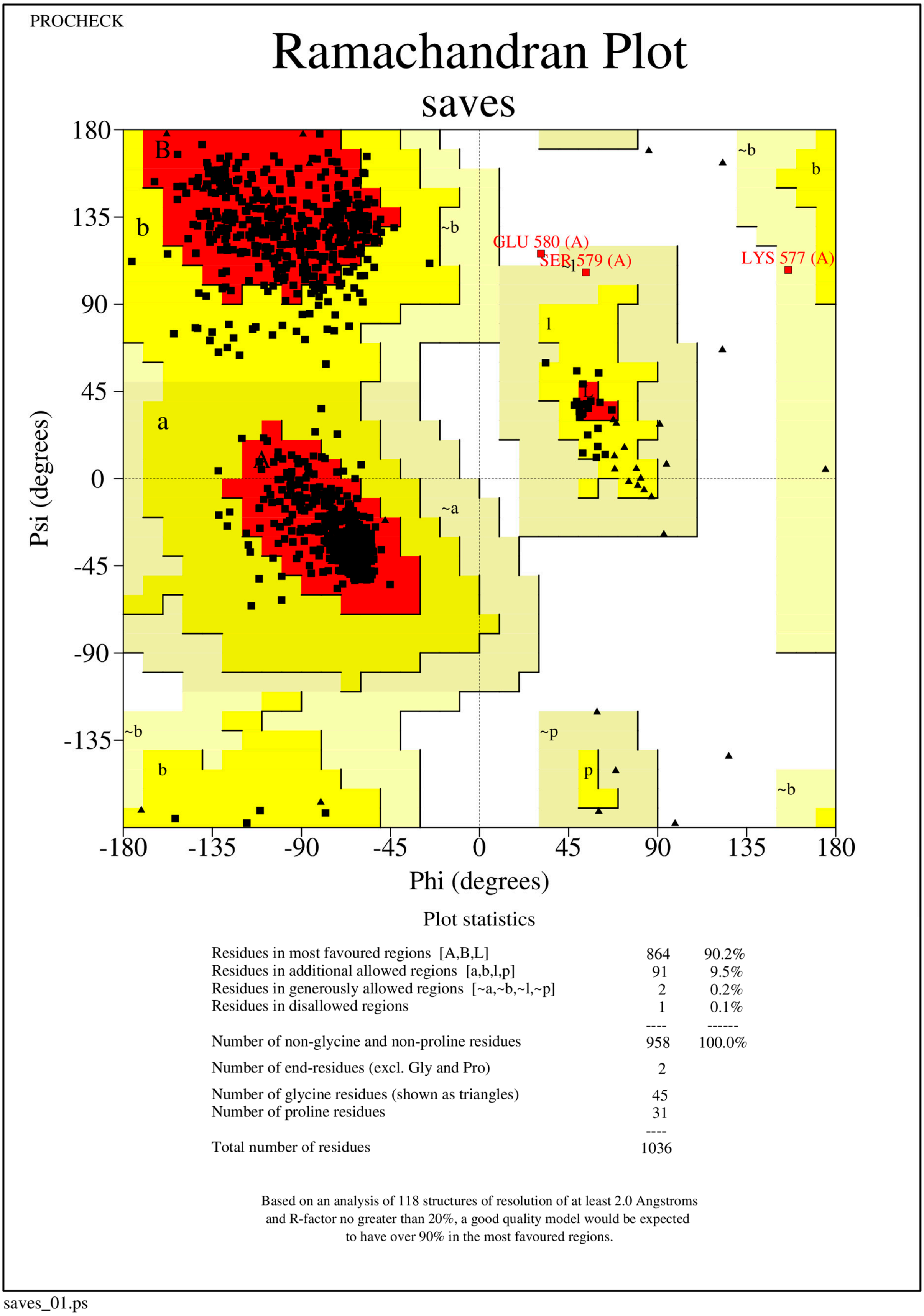

Figure S7. Ramachandran plot analysis (PROCHECK) for the 126 kDa replicase of the ToBRFV_PT2 isolate (PV978368.1).

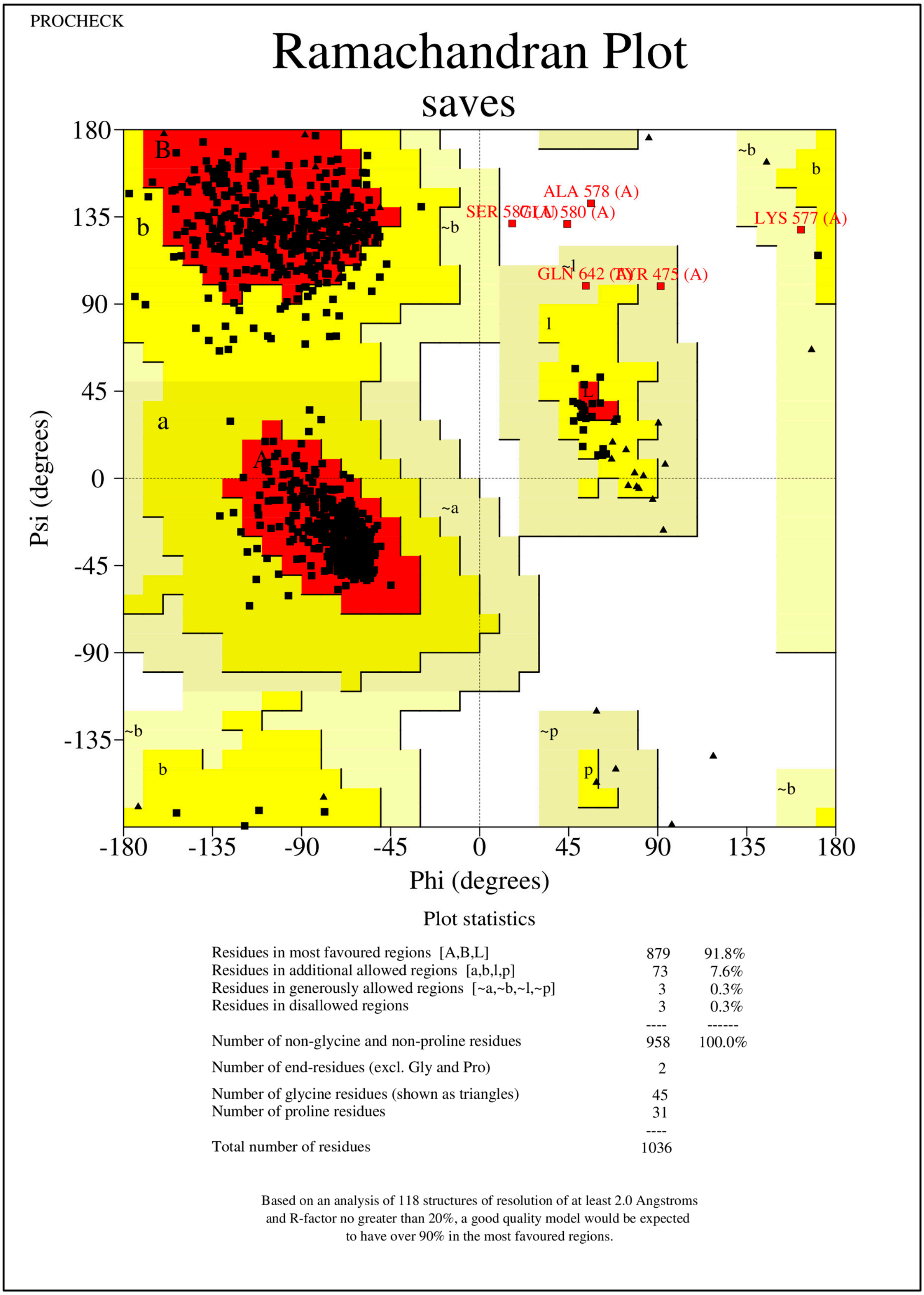

saves_01.ps

Figure S8. Ramachandran plot analysis (PROCHECK) for the 126 kDa replicase of the Israeli isolate (OM_515237.1).

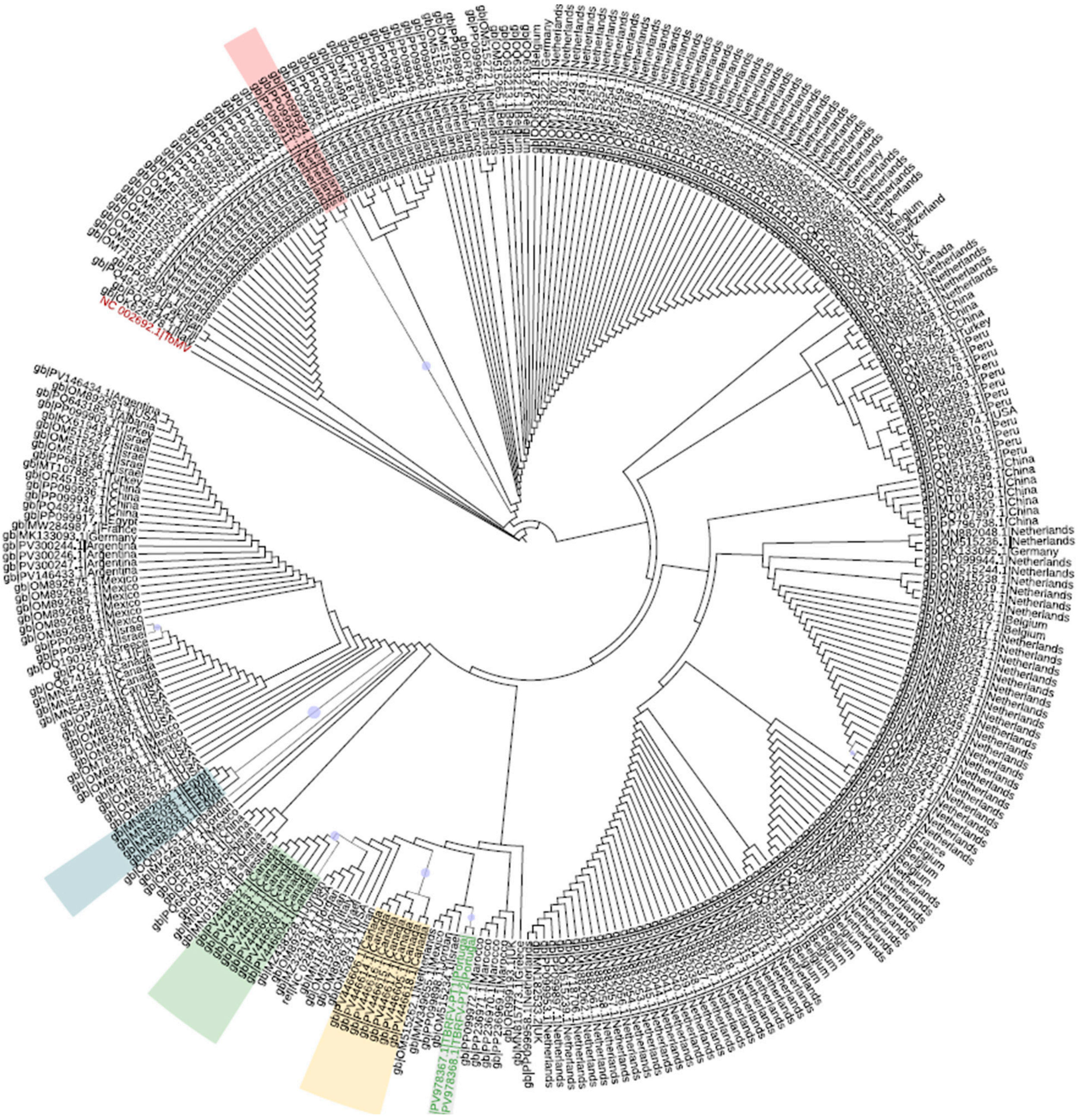

Figure S9. Maximum Likelihood phylogenetic tree inferred from the amino acid sequences of the 183 kDa replicase of ToBRFV isolates. The analysis was performed under the Jones-Taylor-Thornton (1992) model with gamma-distributed rate variation (JTT+G) and 1,000 bootstrap replicates. The tree was rooted using ToMV as an outgroup (red label). Bootstrap support values ≥65% are shown at the nodes, and indicated by purple circles, with larger circles representing higher support. Portuguese isolates are highlighted in green labels. Branch lengths are not scaled to improve visualization. Major clades are highlighted in different colors.

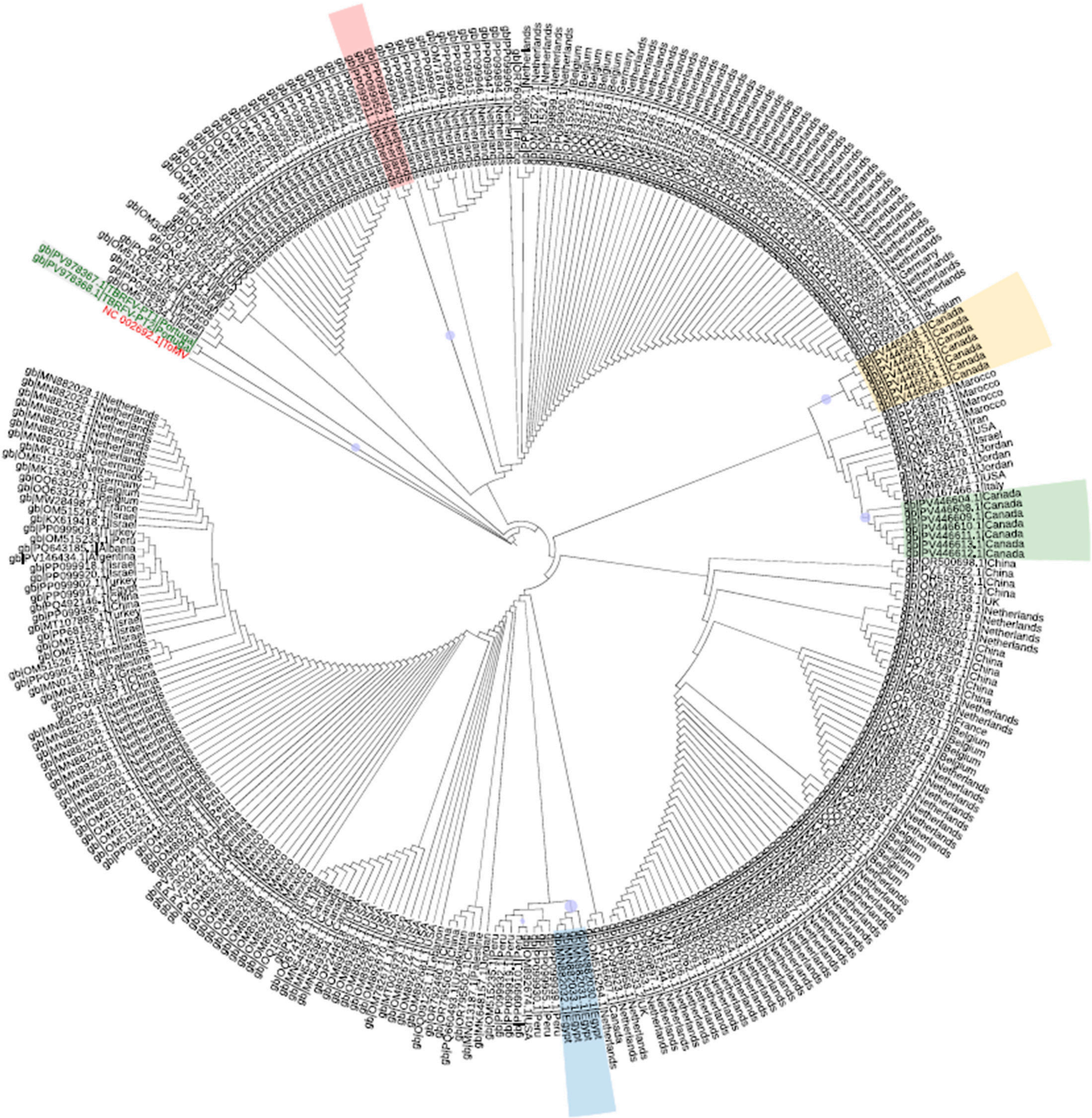

Figure S10. Maximum Likelihood phylogenetic tree inferred from the amino acid sequences of the 126 kDa replicase of ToBRFV isolates. The analysis was performed under the Jones-Taylor-Thornton (1992) model with gamma-distributed rate variation (JTT+G) and 1,000 bootstrap replicates. The tree was rooted using ToMV as an outgroup (red label). Bootstrap support values ≥65% are shown at the nodes, and indicated by purple circles, with larger circles representing higher support. Portuguese isolates are highlighted in green labels. Branch lengths are not scaled to improve visualization. Major clades are highlighted in different colors.

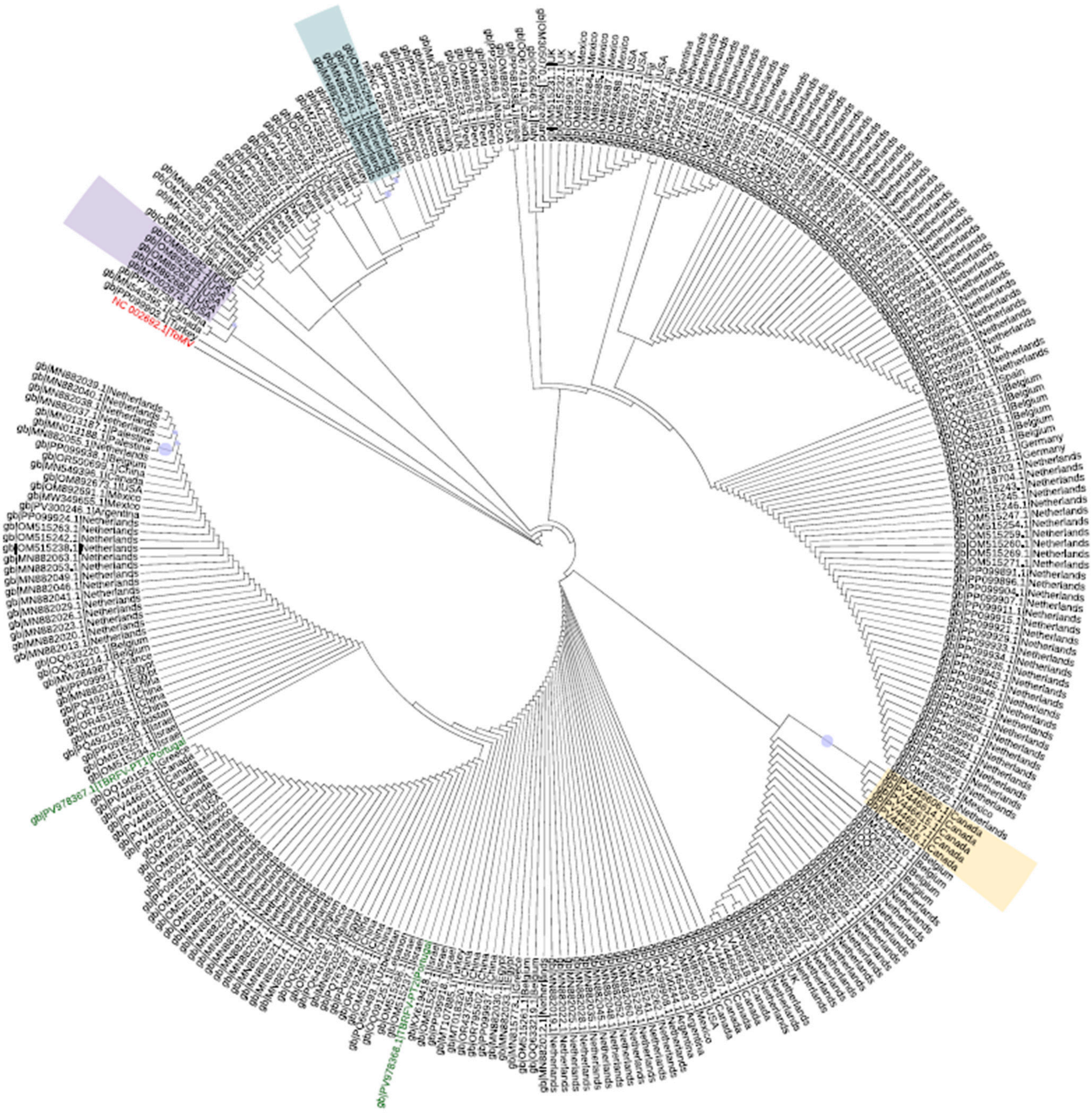

Figure S11. Maximum Likelihood phylogenetic tree inferred from the amino acid sequences of the Movement Protein of ToBRFV isolates. The analysis was performed under the Jones-Taylor-Thornton (1992) model with gamma-distributed rate variation (JTT+G) and 1,000 bootstrap replicates. The tree was rooted using ToMV as an outgroup (red label). Bootstrap support values ≥65% are shown at the nodes, and indicated by purple circles, with larger circles representing higher support. Portuguese isolates are highlighted in green labels. Branch lengths are not scaled to improve visualization. Major clades are highlighted in different colors.

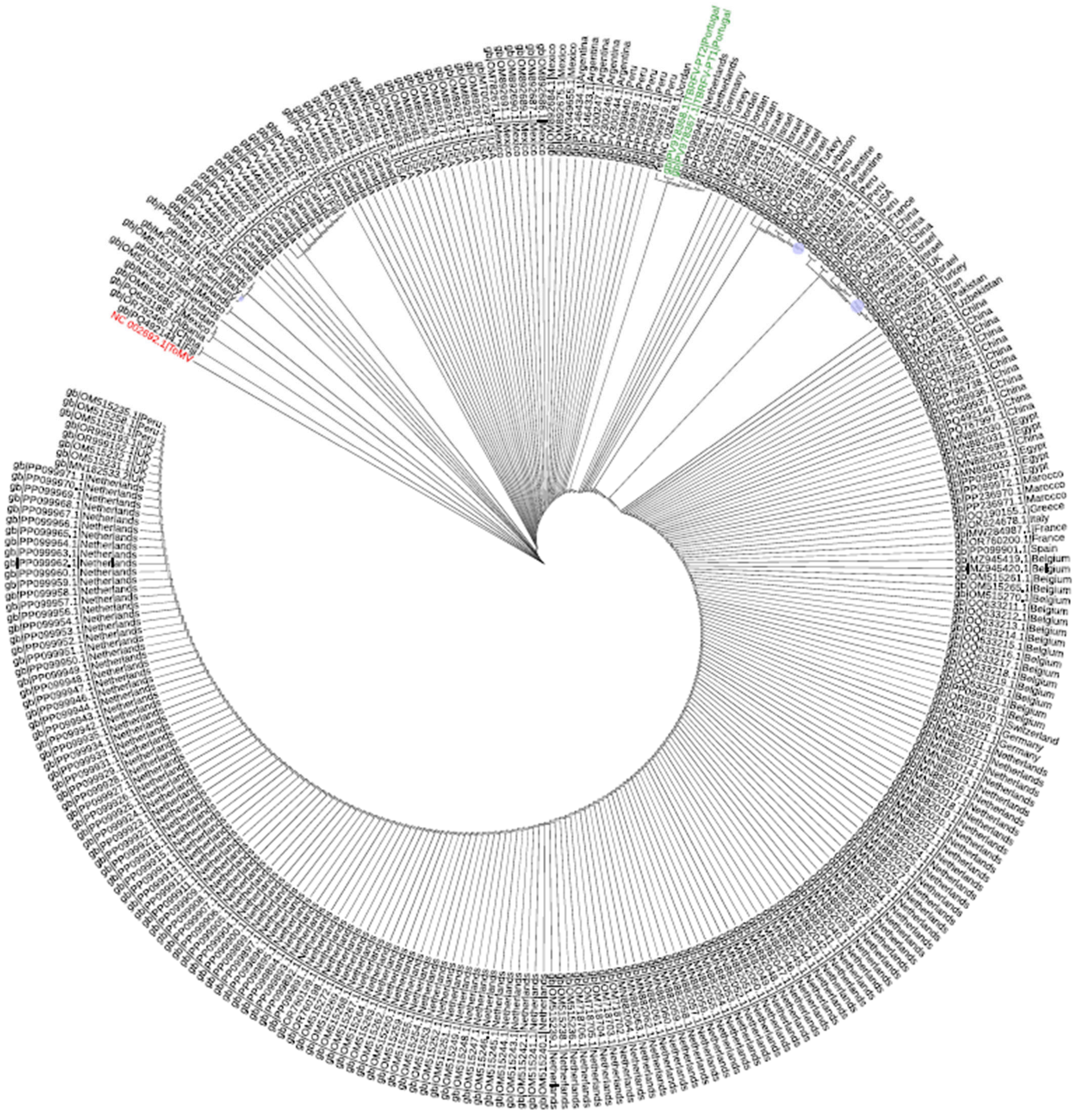

Figure S12. Maximum Likelihood phylogenetic tree inferred from the amino acid sequences of the Coat Protein of ToBRFV isolates. The analysis was performed under the Jones-Taylor-Thornton (1992) model with uniform rates (JTT) and 1,000 bootstrap replicates. The tree was rooted using ToMV as an outgroup (red label). Bootstrap support values ≥65% are shown at the nodes, and indicated by purple circles, with larger circles representing higher support. Portuguese isolates are highlighted in green labels. Branch lengths are not scaled to improve visualization. Major clades are highlighted in different colors.
